# Mitochondria-targeting organoselenium theranostic radioprotectors for simultaneous treatment and imaging of radiation-induced liver injury

**DOI:** 10.7150/thno.133714

**Published:** 2026-05-01

**Authors:** Zifei Wu, Xie Huang, Mingquan Gao, Liting Wang, Zaizhi Du, Ziqian Shang, Xudong Yu, Xiaojiao Wang, Shuyue Deng, Xinrui Yang, Binghui Lu, Jing Liu, Weidong Wang, Rong Li, Shenglin Luo

**Affiliations:** 1 Institute of Combined Injury, State Key Laboratory of Trauma and Chemical Poisoning, Chongqing Engineering Research Center for Nanomedicine, Department of Military Preventive Medicine, Third Military Medical University (Army Medical University), 30 Gaotanyan street, Chongqing 400038, China.; 2 Department of Radiation Oncology, Radiation Oncology Key Laboratory of Sichuan Province, Sichuan Clinical Research Center for Cancer, Sichuan Cancer Hospital & Institute, Affiliated Cancer Hospital of University of Electronic Science and Technology of China, Chengdu 610000, China.; 3 Biomedical Analysis Center, Third Military Medical University (Army Medical University), 30 Gaotanyan street, Chongqing, 400038, China.

**Keywords:** selenium, radiation protection, mitochondria, heptamethine, theranostic

## Abstract

**Rationale:**

Normal liver tissue is vulnerable towards ionizing radiation, which leads to radiation-induced liver injury (RILI) and remains a clinical challenge for treatment. Due to poor tissue targeting, mostly radioprotectors show limited radioprotective effect with a narrow therapeutic window. In this study, we report a series of selenium-substituted heptamethine cyanine derivatives (Se-Cys) as versatile radioprotectors for liver-preferential accumulation, simultaneous RILI imaging and treatment.

**Methods:**

A series of lipophilic cationic Se-Cys were developed with tunable properties for targeted delivery to liver mitochondria. We evaluated their deselenization kinetics in response to H_2_O_2_ and investigated the downstream activation of the Keap1-Nrf2-HO-1 antioxidant axis. Specifically, the derivative Se-Cy4 was assessed for its ROS-responsive fluorescence changes. *In vivo* experiments were conducted to evaluate the dual capacity of Se-Cy4 for dynamic imaging and therapeutic protection against RILI, including systemic toxicity.

**Results:**

The targeting delivery and ROS-responsive selenium release, significantly enhance selenium levels in injured livers and mitochondria induced by ionizing radiation. Deselenization releases bioavailable selenium from Se-Cys, elevates selenoprotein expression and exerts endogenous radioprotective effect by activating the Keap1-Nrf2-HO-1 antioxidant axis. More interestingly, Se-Cys derivatives, especially Se-Cy4, possess a sensitive fluorescent enhancement in H_2_O_2_ concentration-dependent manner, offering a potential application for dynamic monitoring of ROS level in RILI. The *in vivo* results verify that Se-Cy4 has capacities to sensitive fluorescent imaging and efficient treatment of RILI without detectable systemic toxicity.

**Conclusions:**

Se-Cy4 serves as a novel theranostic agent for RILI, by providing ROS monitoring and robust radioprotection without detectable systemic toxicity. Our findings may also promise a potential of selenium-substituted heptamethine cyanines as next-generation radioprotectors for imaging-guided therapy of other radiation-induced diseases.

## Introduction

Ionizing radiation (IR) from natural and artificial sources, including cosmic rays, environmental radionuclides, nuclear activities, accidents, and medical exposure, poses a growing threat to human health 1. In addition to its effects on the hematopoietic and gastrointestinal systems, the liver is particularly vulnerable due to its central role in metabolism, detoxification, blood filtration, and highly vascularized structure 2. Radiation-induced liver injury (RILI) can progress from subtle oxidative and microvascular dysfunction to irreversible fibrosis and liver failure in nuclear-related exposures 3. Early-stage RILI is often clinically silent or masked by pre-existing liver conditions, and conventional biochemical assays and imaging modalities lack sufficient sensitivity to detect these subtle early lesions 4-6. Once liver injury is evident, treatment is limited to supportive care with poor prognosis. Despite extensive efforts, no clinically approved liver-targeted radioprotectors exist that can prevent hepatic damage and detect early radiation-induced oxidative stress, underscoring the need for strategies that integrate effective hepatoprotection with sensitive molecular monitoring of radiation-driven oxidative stress.

Over the past seven decades, significant progress has been made in developing radioprotective agents, yet effective interventions for RILI remain lacking. Various pharmacological approaches, including free radical scavengers, DNA repair enhancers, and immunomodulators, have been explored, with sulfhydryl compounds like amifostine and natural antioxidants such as genistein and resveratrol being the most studied 7-9. Clinically available radioprotective agents remain very limited, and amifostine is among the few approved drugs used in this setting 10. However, in radiotherapy its approved use is restricted to reducing xerostomia in postoperative head-and-neck cancer, and the FDA label advises against routine use in definitive radiotherapy 11. Moreover, amifostine requires intravenous administration shortly before irradiation, together with hydration, antiemetic premedication, and blood pressure monitoring, which complicates routine clinical application. Its adverse effects, especially hypotension, nausea, vomiting, and hypersensitivity, together with heterogeneous efficacy reported across studies 7, underscore the need for safer, more effective, and more broadly applicable next-generation radioprotectors.

Within this context, selenium-based radioprotective agents have emerged as a promising alternative, capitalizing on selenium’s essential role in antioxidant selenoproteins such as glutathione peroxidases (GPXs) and selenoprotein S (SELS), which are crucial for counteracting oxidative stress and radiation-induced damage 12, 13. However, current selenium formulations suffer from poor tissue selectivity, widespread biodistribution, and a narrow therapeutic window, limiting efficacy and increasing the risk of off-target toxicity. Effective doses are close to toxic levels, and dose escalation raises concerns about hepatotoxicity, nephrotoxicity, and immunotoxicity 14, 15. Meanwhile, IR–induced injury is tightly associated with mitochondrial redox imbalance. Radiation provokes an acute burst of ROS via direct ionization and water radiolysis, driving lipid peroxidation, protein oxidation and severe damage to mitochondrial DNA (mtDNA), which lacks robust structural protection and possesses limited self-repair capacity 16, 17. This leads to mitochondrial dysfunction, including loss of mitochondrial membrane potential (MMP), Ca²⁺ dysregulation, and ATP depletion, promoting cell death and organ damage 18, 19. Therefore, targeting delivery of selenium to radiation-induced injured liver tissues and their mitochondria is crucial for developing potent radioprotectors with minimal side effects.

In this study, we design and report a series of selenium-substituted heptamethine cyanine derivatives (Se-Cys) as versatile radioprotectors for liver-specific accumulation, mitochondria targeting, simultaneous RILI imaging and treatment. First, different Se-Cys are designed by tuning their physicochemical properties, including varied lipophilicity for optimal liver accumulation and mitochondrial targeting capacity. Second, Se-Cys as exogenous radioprotectors exhibit high reactive ability to ROS. Their excellent mitochondria accumulation promotes to specifically mitigate radiation-induced mitochondria ROS and guard mitochondria from radiation damage. Third, Se-Cys undergo deselenization process after multi-step reactions with ROS. The targeting delivery and ROS-responsive release ability specifically enhance selenium level in radiated liver cells and mitochondria, elevate selenoprotein expression and exert endogenous radioprotective effect by activating the Keap1–Nrf2–HO-1 antioxidant axis. More interestingly, the resulting cyanine molecules after deselenization process, display an increasing fluorescent intensity in ROS concentration-dependent manner. This fluorescent change may provide a potential application for dynamic monitoring of ROS level in RILI and avoiding overreached antioxidation. The *in vitro* and *in vivo* results testify our design above and successfully screen out the optimal candidate (Se-Cy4) to efficiently alleviate RILI and restore liver function with favorable biocompatibility. This work may not only present a promising radioprotector for RILI, but also offer a paradigm-shift strategy to develop theranostic radioprotectors for simultaneous imaging and therapy of radiation-induced diseases.

## Material and Methods

### General information

Except for specific mentions, chemical reagents were purchased from Sigma-Aldrich (USA) or Titan Scientific (China). Selenium-containing intermediates were purchased from Bide Pharmatech (China). Silica gel (200–300 mesh, Qingdao Marine Chemical, China) was used for column chromatography during the purification of cyanine derivatives. Proton nuclear magnetic resonance (^1^H NMR) spectra were collected on a Bruker AVANCE III 400 MHz spectrometer (Germany). High-resolution mass spectra (HRMS) were acquired using a Bruker ultraflextreme MALDI-TOF mass spectrometer (Germany) or an Orbitrap Exploris™ 120 system (Thermo Fisher Scientific, USA).

### ROS-responsive fluorescence enhancement assay

Aqueous solutions of Se-Cys derivatives (10 μM) were incubated with H_2_O_2_ at various concentrations (0, 10, 50, 100, and 200 μM). After shaking at 37 °C for 10 min, fluorescence spectra were recorded using an F380 fluorescence spectrometer, and near-infrared (NIR) images were acquired with an Aniview 100 imaging system (BLT, China) under 740 nm excitation.

### Evaluation of cytotoxicity and radioprotective effects of Se-Cys

To evaluate the cytotoxicity of Se-Cys, L-02 cells were seeded into 96-well plates at a density of 3,000 cells per well and incubated overnight to allow for adherence. Subsequently, the cells were treated with Se-Cys at various concentrations (0, 1.25, 2.5, 5, 10 μM) for 24 h. Cell viability was then assessed using the Cell Counting Kit-8 (CCK-8) assay and the absorbance at 450 nm was measured using a microplate reader (Thermo Fisher Scientific, USA).

To evaluate the radioprotective effects of Se-Cys, L-02 cells were seeded into 96-well plates at a density of 3,000 cells per well and incubated overnight to allow for adherence. The cells were then treated with Se-Cys at a concentration of 2.5 μM for 6 h, followed by exposure to ^60^Co γ-ray radiation at doses of 2, 4, 6, 8, or 10 Gy. Cell viability was assessed at 24, 48, and 72 h post-radiation.

### Mitochondrial co-localization analysis

L-02 cells were seeded into glass-bottom confocal dishes and incubated for 24 h, followed by treatment with Se-Cy2, Se-Cy4, or Se-Cy5 (2.5 μM) for 6 h. The cells were then stained with MitoTracker™ Green CMXRos (Invitrogen, Thermo Fisher Scientific, USA) for 30 min at 37 °C, washed thoroughly with Phosphate-Buffered Saline (PBS), and resuspended in fresh medium. Fluorescence imaging was conducted using a confocal laser scanning microscope (Leica TCS SP8, Leica Microsystems, Germany). Mitochondrial co-localization was quantified by calculating the Pearson correlation coefficient (PCC) using ImageJ software (version 1.8.0.112, NIH, USA).

### Detection of cytosolic and mitochondrial ROS

To assess cytosolic ROS, L-02 cells were seeded in 6-well plates at 2 × 10^5^ cells per well and incubated for 24 h. Cells were then treated with amifostine (10 μM), Se-Cy2, Se-Cy4, or Se-Cy5 (2.5 μM) for 6 h, followed by exposure to 10 Gy of ^60^Co γ-ray IR. After 48 h, cells were incubated with the ROS-sensitive probe 2′,7′-dichlorofluorescein diacetate (DCFH-DA, Beyotime Biotechnology, China), diluted 1:1000 in serum-free medium to a final concentration of 10 μM for 30 min, followed by three washes with RPMI-1640. Fluorescence was detected using a fluorescence microscope.

For mitochondrial ROS detection, L-02 cells were incubated with 5 μM MitoSOX™ Red Mitochondrial Superoxide Indicator (Yeasen, China) for 10 min, washed with PBS, and counterstained with DAPI for 10 min. Cells were imaged using a confocal laser scanning microscope.

### Mitochondrial membrane potential analysis

L-02 cells were treated with 2.5 μM Se-Cys or 10 μM amifostine for 6 h, followed by exposure to 10 Gy of ^60^Co γ-ray radiation. Cells were then collected and incubated with JC-1 dye (3 μg/mL, Beyotime Biotechnology, China) at 37 °C for 30 min. After staining, cells were washed with PBS, and fluorescence images were captured using a confocal laser scanning microscope. The mitochondrial membrane potential was assessed by calculating the fluorescence intensity ratio of JC-1 aggregates (red) to monomers (green).

### Animal experiment

All animal experiments were conducted in compliance with the ethical guidelines established by the local institutional the Animal Experiment Ethics Committee (approval number: AMUWEC20210301), in accordance with internationally accepted standards for animal research, ensuring adherence to the ethical standards for animal research. The male Balb/c mice, aged 8 weeks, used in this study were sourced from the Animal Breeding Center of Army Medical University. For the whole-body injury model, mice were exposed to a lethal dose of whole-body ^60^Co γ-ray IR (7 Gy). Se-Cy4 was administered intraperitoneally once before irradiation and subsequently every 3 days following the established dosing schedule. Survival was monitored for 30 days post-IR. Mice were randomly allocated into six groups (n = 10 per group): (1) Control, (2) γ-ray only, (3) γ-ray + Amifostine (100 mg/kg), (4) γ-ray + Se-Cy4 (0.5 mg/kg), (5) γ-ray + Se-Cy4 (10 mg/kg), and (6) γ-ray + Se-Cy4 (20 mg/kg). Body weights were measured throughout the 30-day observation period.

For radiation-induced liver injury, mice were randomly assigned to four groups (n = 10 per group): (1) Control, (2) ^60^Co γ-ray, (3) ^60^Co γ-ray + Amifostine, and (4) ^60^Co γ-ray + Se-Cy4. Mice in the treatment groups received intraperitoneal administration of Amifostine or Se-Cy4 (20 mg/kg) 24 hours before irradiation, followed by once every 3 days for a total duration of 7 days. Local abdominal irradiation was performed with a single 15 Gy dose of ^60^Co γ-rays. At the end of the experimental period, mice were euthanized under deep anesthesia, and blood samples were collected for hematological and biochemical analyses. Livers and other major organs were excised for histopathological and molecular evaluations.

### Statistical analysis

Data are expressed as the mean ± standard deviation (SD), unless stated otherwise. Statistical analysis was performed using GraphPad Prism 9.0 (GraphPad Software, USA). For comparisons involving multiple groups, one-way analysis of variance (ANOVA) followed by Tukey's post hoc test was conducted. A *P*-value of less than 0.05 was considered statistically significant.

## Results

### Design, synthesis, and characterization of Se-Cys derivatives

**Figure 1:**
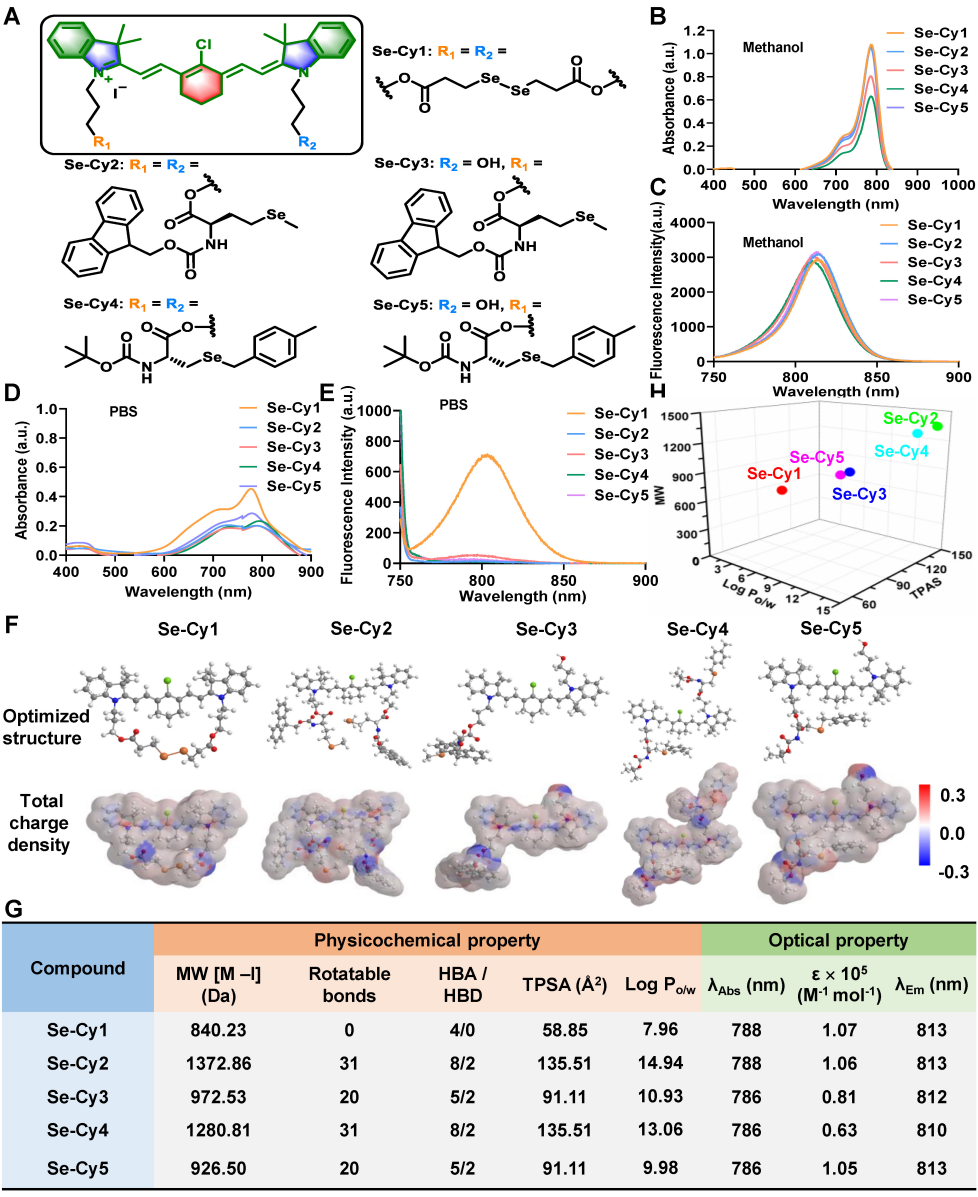
**The structures of Se-Cys derivatives and their corresponding physicochemical properties. (A)** Chemical structures of Se-Cys derivatives with a mitochondria-targeting scaffold (colored). **(B)** UV-vis absorption spectra and **(C)** fluorescence emission spectra of Se-Cys derivatives in methanol solution.** (D)** UV-vis absorption spectra and** (E)** fluorescence emission spectra of Se-Cys derivatives in PBS solution.** (F)** Optimized structures and corresponding total charge densities of Se-Cy1, Se-Cy2, Se-Cy3, Se-Cy4, and Se-Cy5. **(G)** The physicochemical and optical properties of Se-Cys derivatives. The maximum absorption wavelength (λ_Abs_), molar extinction coefficient (ε), and maximum emission wavelength (λ_Em_) were obtained from Figure **1B** and **C**. **(H)** The relationship between MW, TPSA and Log P_o/w_ of Se-Cys derivatives.

In our previous work, heptamethine cyanine dyes with lipophilic cationic properties can preferentially accumulate in mitochondria 20-23. Thus, selenium-substituted heptamethine cyanine derivatives (Se-Cys) were synthesized in this work. By tuning physicochemical properties, including varied lipophilicity and reactive activity towards ROS, mono-substitution and di-substitution of selenium were incorporated into this mitochondria-targeting scaffold to yield Se-Cys derivatives, Se-Cy1, Se-Cy2, Se-Cy3, Se-Cy4, Se-Cy5 (**Figure 1A** and **Figure S1**). The structures of all Se-Cys derivatives were confirmed by ^1^H NMR and HRMS characterization (**Figure S2–S12**). All derivatives exhibited absorption and emission peaks within the NIR range (700 nm - 900 nm) in methanol **Figure 1B**-**C**), which is advantageous for *in vivo* NIR imaging 24. However, except for Se-Cy1, the other four derivatives (Se-Cy2 - Se-Cy5) showed weak absorption and nearly undetectable fluorescence emission in aqueous solutions (**Figure 1D**-**E**). This behavior was likely due to their high hydrophilicity, which induces aggregation in aqueous environments and leads to aggregation-induced fluorescence quenching 25.

Next, SwissADME software was used to calculate the physicochemical properties of the Se-Cys derivatives, including molecular weight (MW), rotatable bonds, numbers of hydrogen bond acceptors (HBA) and donors (HBD), topological polar surface area (TPSA), and oil-water partition coefficient (Log Po/w). The dispersed charge densities of Se-Cys derivatives might also offer significant differences in their lipophilic-hydrophilic properties and eletrophilicity (**Figure 1F**). Additionally, the disubstituted derivatives Se-Cy2 and Se-Cy4 both exhibited the highest theoretical TPSA values (135.51) among these five molecules (**Figure 1G**), suggesting a highly polar surface. Besides, the derivatives Se-Cy2 and Se-Cy4 with larger molecular weight showed higher Log P values (14.94 and 13.06, respectively), while Se-Cy1 with the smallest molecular weight exhibited the lowest Log P value (7.96). The MW, TPSA, and Log P of mono-substituted or di-substituted derivatives exhibited a regular and discrete distribution pattern (**Figure 1H**), indicating their distinctive pharmacokinetic profiles 26. Thus, Se-Cys derivatives were synthesized with varied physicochemical properties, providing candidates for the screening evaluation of ROS scavenging, mitochondrial targeting, and radiopretective abilities.

### ROS reactive ability and ROS-responsive fluorescence change of Se-Cys

**Figure 2:**
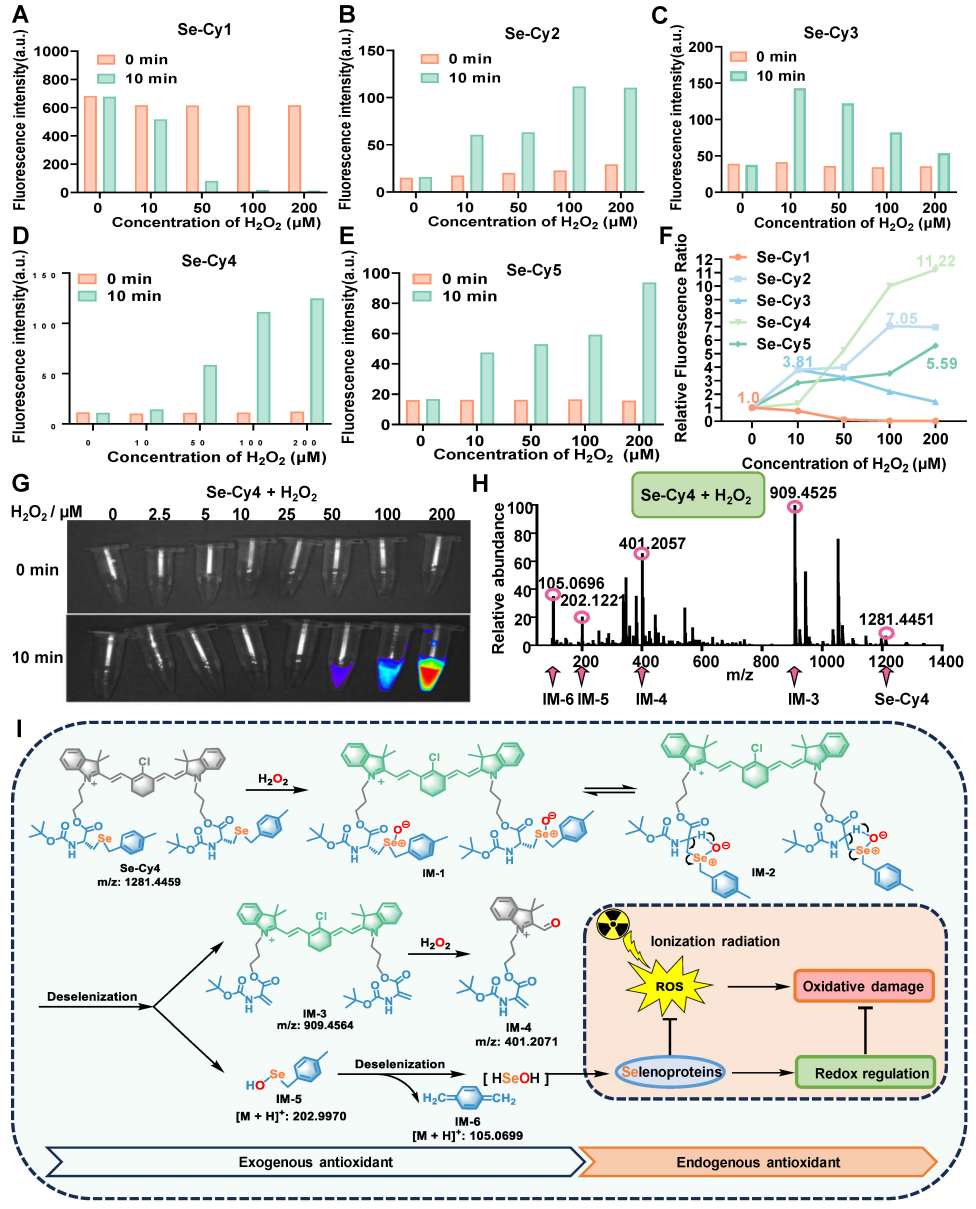
**ROS reactivity and fluorescent changes of Se-Cys derivatives toward H_2_O_2_. (A-E)** Fluorescence intensity changes at maximum emission wavelength (740 nm) before and after reaction with H_2_O_2_. **(F)** The fluorescence enhanced folds of Se-Cys derivatives at 0 min (before) and 10 min (after) incubated with different concentrations of H_2_O_2_ in aqueous solution. **(G)** Near-infrared imaging of Se-Cy4 towards different concentrations (0-200 μM) of H_2_O_2_ in aqueous solutions. **(H)** HRMS analysis of Se-Cy4 and H_2_O_2_ mixture. **(I)** The proposed reaction mechanism of Se-Cy4 towards H_2_O_2_ and process of deselenation.

To assess the reactivity of Se-Cys derivatives towards ROS, their spectral changes were measured at varying hydrogen peroxide (H_2_O_2_) concentrations. The fluorescence intensity of Se-Cy1 decreased with increasing H_2_O_2_ concentrations (**Figure 2A** and **Figure S13A-B**). In contrast, the other four Se-Cys derivatives (Se-Cy2 - Se-Cy5) exhibited increasing fluorescence with rising H_2_O_2_ concentrations in aqueous solution (**Figure 2B-E** and **Figure S13C-J**). Among them, Se-Cy4 exhibited the most significant fluorescence enhancement (11-fold) over a wide range of H_2_O_2_ concentrations (**Figure 2F**), indicating its high ROS reactivity and suitability as a NIR fluorescent probe for ROS detection. In addition to H_2_O_2_, IR can generate multiple ROS species, particularly hydroxyl radicals (**·**OH) and superoxide anions (O_2_**^·^**⁻). Additional electron spin resonance (ESR) experiments were therefore performed to further evaluate the ROS-scavenging capacity of Se-Cy4. Consistent with this, Se-Cy4 markedly attenuated the ESR signal intensities of both O_2_**^·^**⁻ and **·**OH compared with the control group (**Figure S14**), confirming its effective scavenging activity toward multiple ROS species. In addition, the selectivity and anti-interference performance of Se-Cy4 were examined under complex *in vitro* conditions. Se-Cy4 displayed a strong fluorescence response to H_2_O_2_, but negligible responses to various biologically relevant amino acids, thiols, and metal ions (**Figure S15**), demonstrating its high selectivity and good anti-interference capability for ROS detection. The ROS-responsive fluorescence of Se-Cy4 was further visualized using an *in vivo* NIR fluorescence imaging system (**Figure 2G**). Together, these results demonstrate that Se-Cys derivatives, particularly Se-Cy4, possess both ROS-scavenging activity and ROS-responsive fluorescence enhancement.

To investigate the mechanism of ROS-responsive fluorescence enhancement, HRMS analysis was conducted to detect structural changes after Se-Cy4 reacted to H_2_O_2_. The molecular ion peak of Se-Cy4 and several fragment ion peaks (IM-3, IM-4, IM-5, IM-6) were observed in the mixture with H_2_O_2_ (**Figure 2H**), supporting Se-Cy4's reactivity with H_2_O_2_ and subsequent selenium release through multistep reactions (**Figure 2I**). In this process, selenium atoms in Se-Cy4 are first oxidized by H_2_O_2_ to form intermediate IM-127 27. The resonance-stabilized cyclic intermediate IM-2 undergoes an elimination reaction, producing key intermediates IM-3 and IM-5 28. Subsequently, the intermediate IM-3 undergoes further oxidation and cleavage to afford IM-4 29. Finally, intermediate IM-5 undergoes an elimination reaction to yield IM-6, accompanied by the release of HSeOH, which serves as a potential selenium source for selenoprotein synthesis 30. Combining with ROS clearance as exogenous antioxidants, the synthetic selenoproteins may activate endogenous antioxidant pathways, collectively alleviating oxidative damage induced by ionizing radiation.

### DFT calculations and proposed mechanisms for fluorescence enhancement

**Figure 3:**
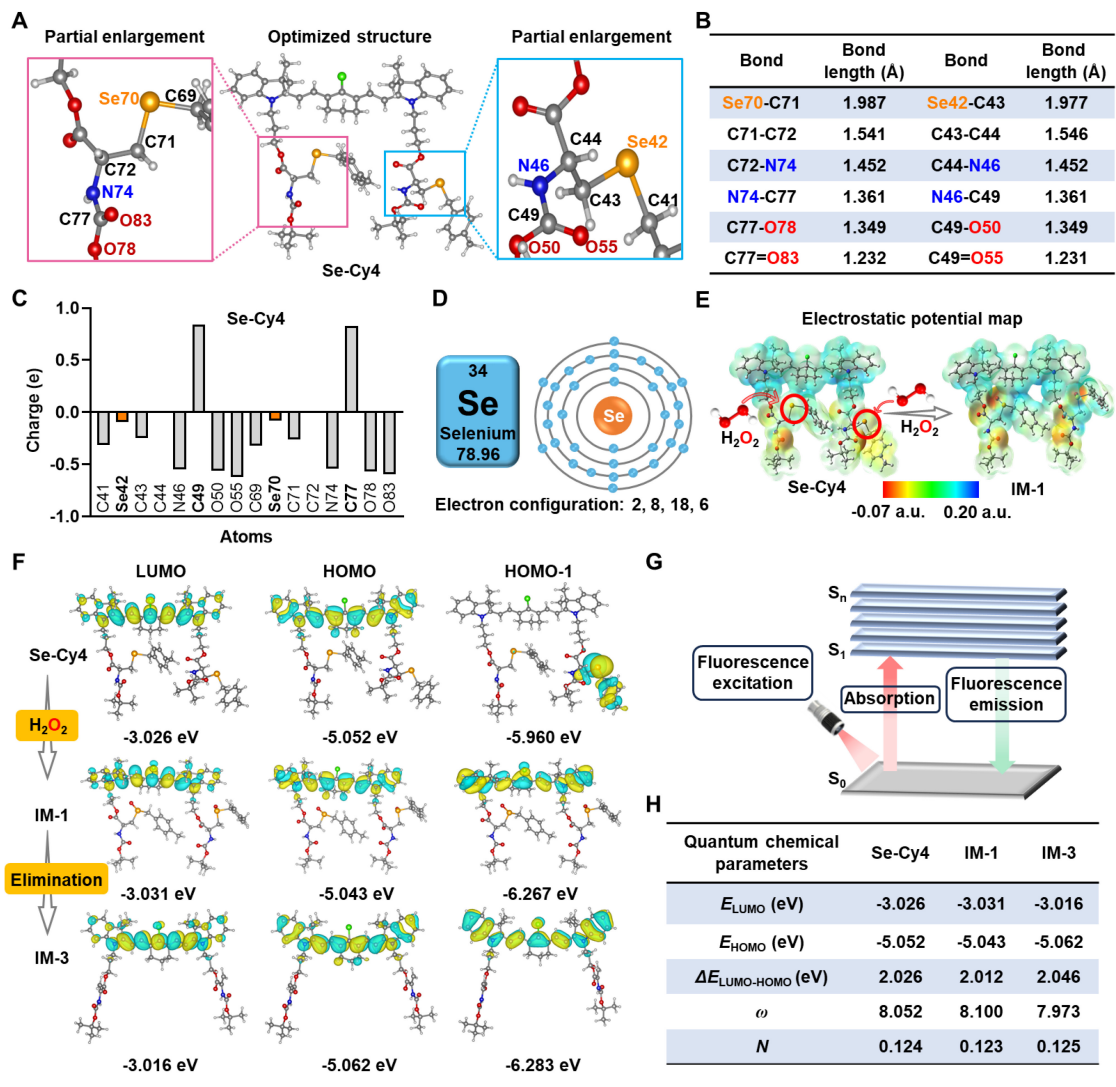
**DFT calculations of Se-Cy4 and its corresponding products after reacted to H_2_O_2_.** Optimized structure **(A)**, bond lengths **(B)**, and Mulliken charges **(C)** of Se-Cy4.** (D)** The schematic diagram of electron configuration of selenium atom. **(E)** The electrostatic potential maps of Se-Cy4 and IM-1. **(F)** Frontier molecular orbitals of Se-Cy4, IM-1 and IM-3. **(G)** Schematic illustrating the principle of fluorescence emission. **(H)** Quantum chemical parameters of Se-Cy4, IM-1 and IM-3. Parameters: lowest unoccupied molecular orbital (*E*_LUMO_), highest occupied molecular orbital (*E*_HOMO_), energy gap between *E*_LUMO_ and *E*_HOMO_ (*ΔE*_LUMO-HOMO_), electrophilicity index (*ω*), nucleophilicity index (*N*).

DFT calculations were performed to evaluate the optimized structures and electrostatic potentials of Se-Cy4 and its fragments (IM-1 to IM-6, **Figure S16**), providing insights into the different reactivity and fluorescence enhancement mechanisms towards ROS. In the selenium-containing ROS reaction center of Se-Cy4, the bond lengths of selenium-carbon bonds (Se70-C71 and Se42-C43) are significantly longer than those of the adjacent carbon-carbon (C-C) bonds, carbon-nitrogen (C-N) bonds, carbon-oxygen (C-O) single bonds, and C-O double (C=O) bonds (**Figure 3A-B**). However, longer bond lengths usually suggest lower bond energy and higher reactivity. The electrostatic potential is generally considered predictive of chemical reactivity, as regions with positive and negative potential are typically associated with electrophilic and nucleophilic attack sites 31, 32. Owing to significant steric hindrance from adjacent atoms, the positively charged C49 and C77 atoms are hardly attacked by nucleophilic H_2_O_2_ (**Figure 3C**). In contrast, due to the negligible steric hindrance and unfilled valence electron shells of selenium atoms (**Figure 3D**), the Se42 and Se70 atoms of Se-Cy4 with weak negative potential are more susceptible to nucleophilic attack by H_2_O_2_, resulting in their oxidation and the formation of intermediate IM-1 (**Figure 3E**). Moreover, for cyanine scaffold compounds (Se-Cy4 and IM-1 - IM-3), the electron clouds of HOMO and LUMO molecular orbitals are both distributed throughout the cyanine skeleton (**Figure 3F** and **Figure S17A**). In contrast to IM-1- IM-3, the electron clouds of Se-Cy4 located on HOMO-1 molecular orbital are completely concentrated on the side chain rather than the cyanine skeleton. Under fluorescence excitation, photonic energy is absorbed by the electrons in the molecule, causing the electrons to transition from the ground electronic state (S_0_) to higher excited electronic states (S_1_ - S_n_), thereby generating fluorescence emission (**Figure 3G**). Therefore, the higher electron cloud density on cyanine fluorophore tends to promote stronger fluorescence emission 33, 34, offering a reasonable explanation for the fluorescence increase upon H_2_O_2_ stimulation. Most importantly, compared to IM-4 – IM-6, the narrower HOMO-LUMO energy gaps of Se-Cy4 and IM-1 – IM-3 may be beneficial to be attacked by nucleophilic species such as ROS (**Figure S17B**) 35. Furthermore, the calculated high electrophilicity index (*ω*) values and low nucleophilicity index (*N*) values of Se-Cy4, IM-1 and IM-3 also offer a theoretical foundation for high ROS reactivity (**Figure 3H** and **Table S1**) 36, 37.

### Se-Cy4 mitigates radiation-induced cellular damage

The cytotoxicity of Se-Cys derivatives was assessed in L-02 liver cells using the CCK-8 assay. As shown in **Figure S18**, Se-Cy1 displayed obvious concentration-dependent cytotoxicity, with cell viability decreasing to 55.1% at 10 μM. By contrast, Se-Cy2, Se-Cy3, Se-Cy4, and Se-Cy5 exhibited minimal cytotoxicity over the tested concentration range and remained well tolerated even at 10 μM. The observed differences in cytotoxicity may be related to variations in physicochemical properties, such as LogP, polarity, molecular size, and structural flexibility, which could influence cellular uptake and intracellular interactions. Therefore, Se-Cy2–Se-Cy5 at 2.5 μM were selected for subsequent biological studies.

**Figure 4:**
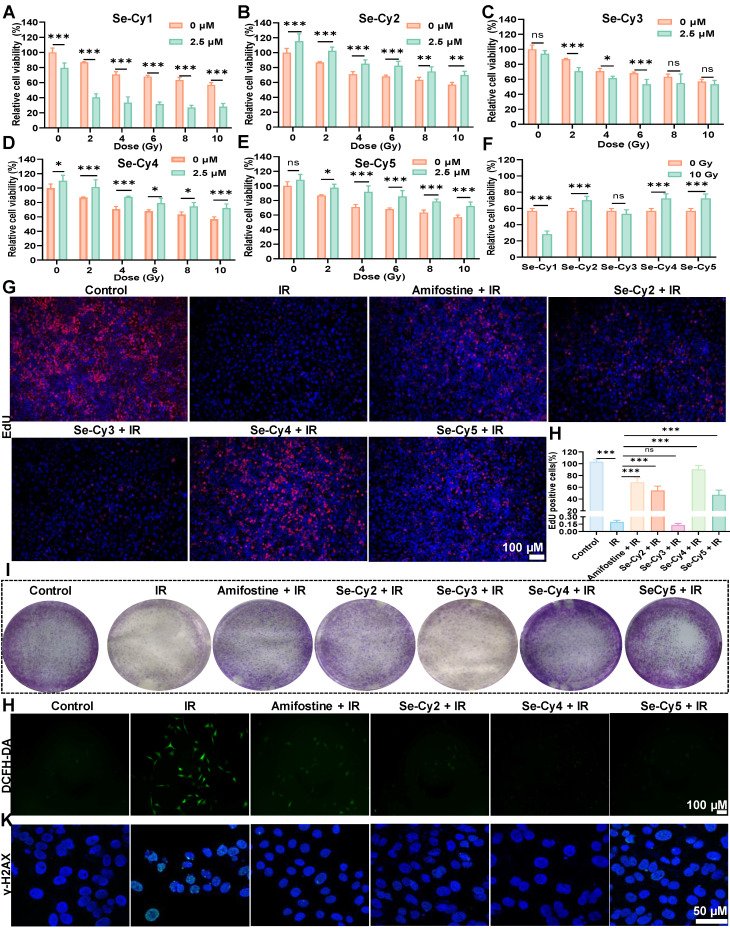
** Se-Cys derivatives guard L-02 cells from IR-induced damage. (A–E)** Cell viability of L-02 cells at 48 h after treated with a series of Se-Cys derivatives (Se-Cy1 to Se-Cy5, 2.5 μM) under different doses of IR (0-10 Gy). **(F)** Comparative radioprotective efficacy of Se-Cys derivatives against 10 Gy IR. Representative images **(G)** and quantification **(H)** of EdU proliferation assays showing the effects of Se-Cys derivatives on cell proliferation after IR. Scale bars, 100 μm.** (I)** Colony formation assay evaluating the clonogenic survival of irradiated cells following treatment with Se-Cys derivatives or amifostine. **(J)** Intracellular ROS levels detected by DCFH-DA staining in each group. Scale bars, 100 μm. **(K)** Immunofluorescence analysis of phosphorylated γ-H2AX foci in each group. Scale bars, 50 μm. Data are presented as mean ± SD. Statistical significance was determined using one-way ANOVA followed by Tukey’s post hoc test. *P < 0.05, **P < 0.01, ***P < 0.001.

To investigate their radioprotective potential, Se-Cys derivatives were tested in L-02 cells exposed to ^60^Co γ-radiation. As illustrated in **Figure 4A–F**, cell viability decreased in a dose-dependent manner within 48 h post-IR, with survival rates of 86.0%, 70.9%, 67.7%, and 59.2% at 4, 6, 8, and 10 Gy, respectively. Pretreatment with Se-Cy2, Se-Cy4, and Se-Cy5 markedly improved cell survival, among which Se-Cy4 provided the most pronounced protection. Specifically, Se-Cy4 increased viability by by 18.7% at 72 h (**Figure S19**) compared with 10 Gy irradiated controls. Additional assays, including EdU incorporation (**Figure 4G-H**), colony formation (**Figure 4I**), and Calcein-AM/PI double staining (**Figure S20**), further confirmed that Se-Cy4 most effectively restored cell proliferation and survival following IR. Consistent with these observations, apoptosis analysis (**Figure S21**) revealed a marked reduction after Se-Cys pretreatment, with Se-Cy4 outperforming even amifostine. Given that IR predominantly induces DNA double-strand breaks (DSBs), either directly or through ROS-mediated damage, thereby activating the DNA damage response (DDR) to initiate cell-cycle arrest and repair, we further evaluated oxidative and genotoxic stress. DCFH-DA staining revealed marked ROS accumulation in irradiated cells, whereas pretreatment with Se-Cys derivatives substantially suppressed ROS generation (**Figure 4J** and **Figure S22**)—comparable to the effect of amifostine, a clinically approved radioprotective agent. Among the derivatives, Se-Cy4 exhibited the strongest antioxidant activity, nearly eliminating the IR-induced ROS signal. Immunofluorescence analysis of γ-H2AX, a marker of DSBs, showed extensive nuclear foci formation following IR, while pretreatment with Se-Cys derivatives significantly reduced γ-H2AX intensity and foci number (**Figure 4K** and **Figure S23**). Notably, Se-Cy4 nearly restored nuclear morphology to that of untreated controls, indicating robust protection against IR-induced DNA damage.

Since IR-induced DNA double-strand breaks can trigger DNA damage–dependent checkpoints, we next examined whether Se-Cys derivatives mitigate the resulting cell-cycle arrest. As indicated in **Figure S24**, 10 Gy IR induced a strong G2/M arrest, increasing the G2/M population from 5.98% in controls to 55.54%. Pretreatment with Se-Cy4 or Se-Cy5 substantially alleviated this arrest, reducing the G2/M fraction to 27.16% and 27.18%, respectively—representing a 1.9-fold decrease relative to IR alone. Collectively, these results demonstrate that Se-Cy4 provides potent radioprotection in L-02 cells by suppressing ROS accumulation, alleviating DNA damage, maintaining normal cell-cycle progression, and enhancing overall cell survival following IR.

### Se-Cys guard mitochondria from radiation-induced damage

**Figure 5:**
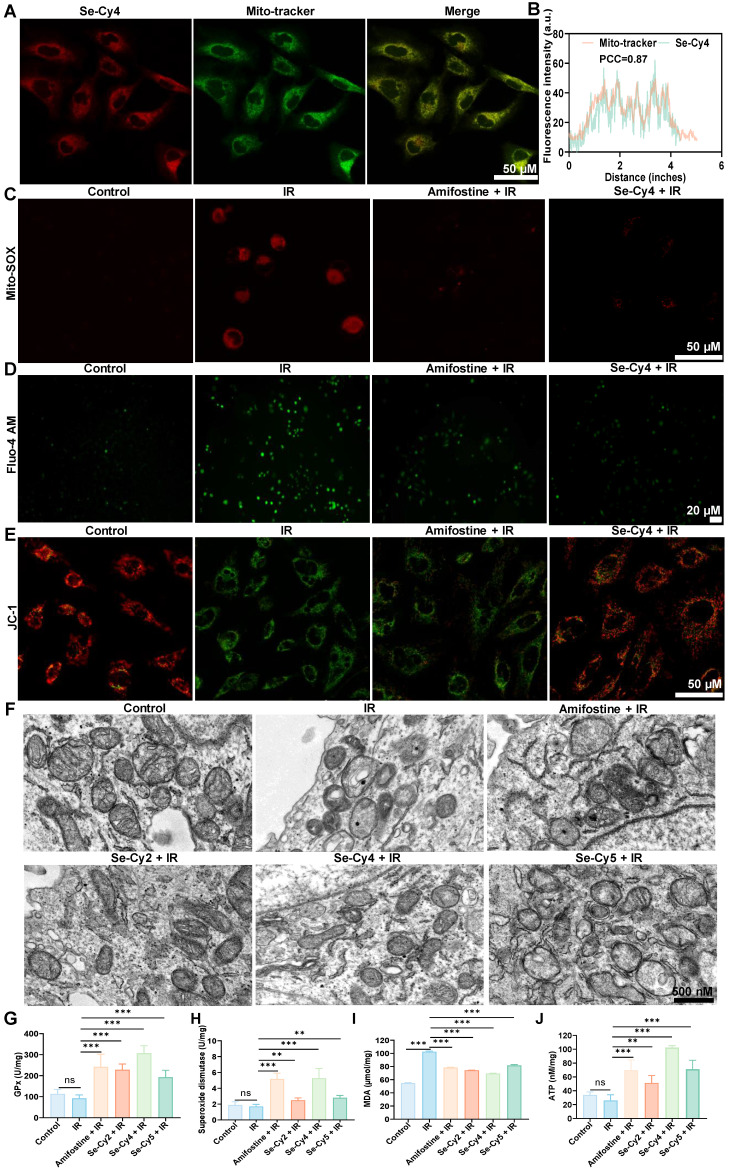
** Se-Cys derivatives protect against radiation-induced mitochondrial dysfunction in L-02 cells. (A)** Confocal fluorescence images showing the co-localization of Se-Cys derivatives (Se-Cy4, red) with MitoTracker (green). Scale bars, 50 μm.** (B)** Line profile analyses and Pearson’s correlation coefficients (PCC) quantifying mitochondrial co-localization of Se-Cy4.** (C)** Mitochondrial reactive oxygen species (mtROS) level detected by MitoSOX™ Red staining in each group.** (D)** Intracellular Ca²⁺ levels measured with Fluo-4 AM probe in each group.** (E)** MMP assessed by JC-1 staining in each group, showing green fluorescence for monomers and red fluorescence for aggregates. **(F)** TEM images showing mitochondrial ultrastructure in each group, boxed areas are enlarged below. Scale bars, 500 nm. **(G–J)** Quantification of cellular antioxidant and metabolic parameters in each group, including GPx activity **(G)**, SOD activity **(H)**, MDA content **(I)**, and intracellular ATP levels** (J)**. Data are presented as mean ± SD. Statistical significance was determined using one-way ANOVA followed by Tukey’s post hoc test. *P < 0.05, **P < 0.01, ***P < 0.001.

Given that these Se-Cys derivatives retain the mitochondria-targeting scaffold, we further investigated their mitochondrial targeting ability. The results revealed that the red fluorescence from Se-Cys and the green fluorescence from Mito-Tracker co-localized to a significant extent, producing an orange signal (**Figure 5A** and **Figure S25A**), indicating specific localization within the mitochondria. Additionally, the Pearson’s correlation coefficients for Se-Cy2, Se-Cy4, and Se-Cy5 were 0.86, 0.87, and 0.82, respectively (**Figure 5B** and **Figure S25 B-D**), further confirming the specific mitochondrial localization of these derivatives. Importantly, Se-Cy4 maintained strong colocalization with Mito-Tracker in irradiated liver cells (**Figure S26**), with a high Pearson’s correlation coefficient further confirming that its mitochondrial targeting and retention ability was not significantly affected by IR.

IR is known to trigger excessive mitochondrial ROS (mtROS) generation, thereby perturbing cellular redox balance and promoting oxidative damage. As expected, exposure to IR resulted in a significant increase in mtROS fluorescence intensity. However, pretreatment with Se-Cys derivatives, especially Se-Cy4, effectively suppressed this increase (**Figure 5C** and **Figure S27**). This indicates that Se-Cys derivatives possess potent ROS-scavenging capacity within mitochondria, comparable to surpassing that of the clinical radioprotector amifostine. Intracellular Ca²⁺ homeostasis is another critical determinant of mitochondrial stability. Following IR, a pronounced calcium overload was observed via Fluo-4 AM staining (**Figure 5D** and **Figure S28**), consistent with previous findings that IR impairs calcium pump function and promotes mitochondrial permeability transition. Notably, Se-Cys pretreatment markedly alleviated Ca²⁺ accumulation, suggesting that these compounds may stabilize calcium transport and prevent IR-induced mitochondrial dysfunction. MMP serves as a key indicator of mitochondrial integrity. In irradiated cells, JC-1 staining revealed a dramatic shift from red to green fluorescence, corresponding to depolarization of MMP (**Figure 5E** and **Figure S29**). However, Se-Cy4 pretreatment largely preserved MMP, maintaining a red-dominant fluorescence pattern similar to that of the control group. This protection implies that Se-Cys derivatives safeguard the electron transport chain and sustain mitochondrial bioenergetic function under oxidative stress. Given the close link between intracellular pH homeostasis and cellular stress responses 38, intracellular pH-related fluorescence was further assessed in irradiated liver cells. IR markedly reduced the pH-sensitive fluorescence signal, indicating intracellular acidification, whereas pretreatment with amifostine or Se-Cys derivatives significantly alleviated this decrease (**Figure S30**), suggesting that these Se-Cys derivatives help preserve intracellular pH homeostasis under IR-induced oxidative stress. Transmission electron microscopy provided ultrastructural confirmation of these findings (**Figure 5F**). IR exposure induced severe mitochondrial swelling, cristae fragmentation, and outer membrane rupture, all of which are hallmarks of mitochondrial injury. Amifostine pretreatment partially mitigated these structural abnormalities, whereas Se-Cy4 offered more pronounced protection, maintaining intact membranes and well-defined cristae. These morphological improvements highlight the superior mitochondrial-preserving efficacy of Se-Cy4.

Biochemical analyses further substantiated these protective effects. Se-Cy4 treatment significantly increased mitochondrial content and enhanced the activities of key antioxidant enzymes, including glutathione peroxidase (GPX) and superoxide dismutase (SOD) (**Figure 5G-H**). To further evaluate the protective effect of Se-Cy4 against IR-induced lipid peroxidation, Liperfluo staining (**Figure S31**) and malondialdehyde (MDA) analysis (**Figure 5I**) were performed in irradiated cells. Both assays showed that IR exposure markedly increased lipid oxidative damage, as evidenced by enhanced Liperfluo fluorescence and elevated MDA levels, whereas pretreatment with either amifostine or Se-Cy4 significantly attenuated these changes. Moreover, Se-Cy4 restored intracellular ATP levels and increased mitochondrial Se content (**Figure 5J** and **Figure S32**). Taken together, these results demonstrate that Se-Cys derivatives, especially Se-Cy4, effectively target mitochondria, increase mitochondrial Se content, and protect against IR-induced mitochondrial oxidative damage and dysfunction.

### Se-Cy4 enables sensitive imaging of RILI

**Figure 6:**
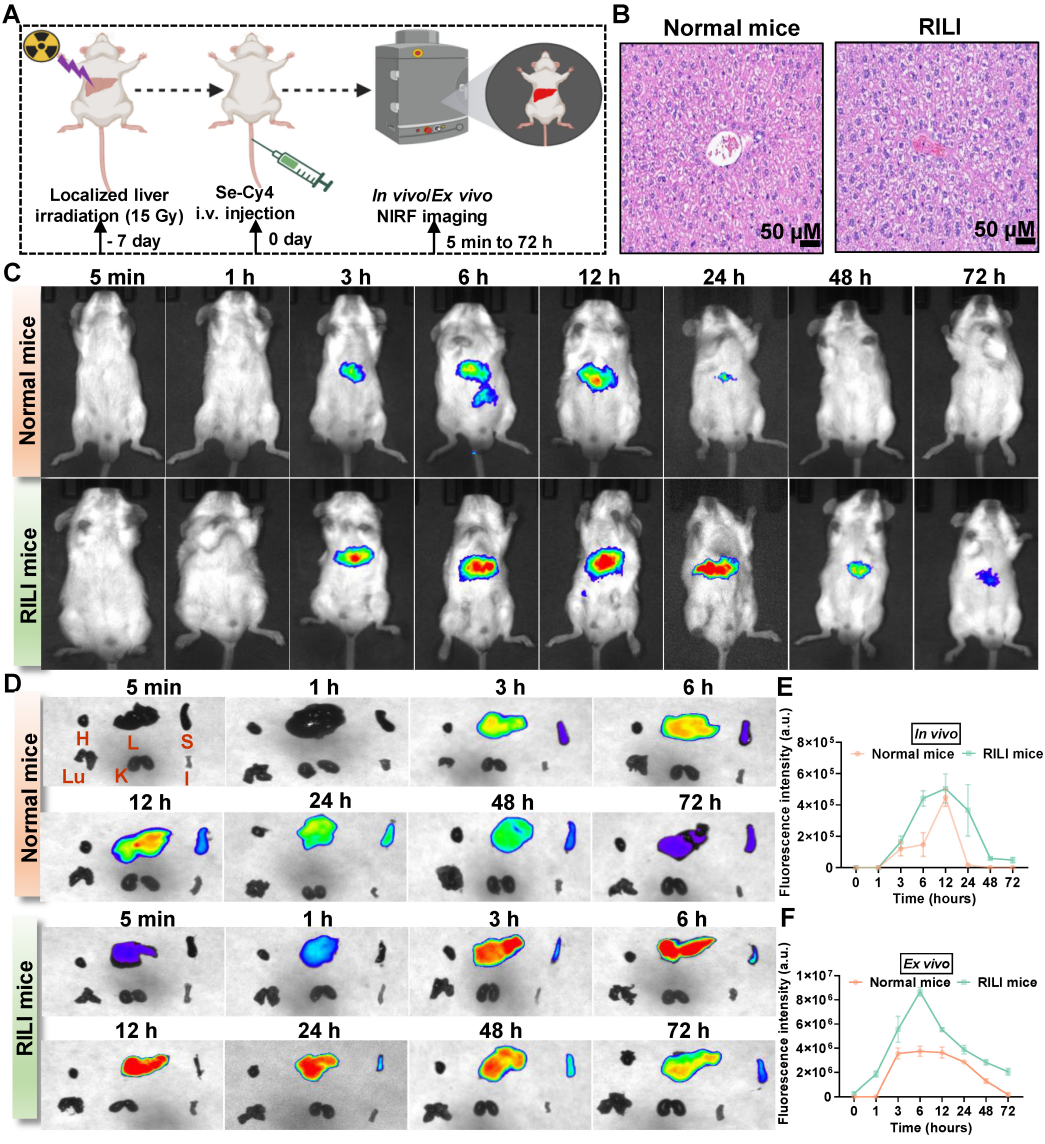
** Se-Cy4 enables near-infrared fluorescent imaging of radiation-induced liver injury *in vivo*. (A)** Schematic illustration of the experimental design showing abdominal IR (15 Gy) administered 7 days before Se-Cy4 injection, followed by *in vivo* and* ex vivo* NIR fluorescent imaging at the indicated time points. **(B)** Representative H&E-stained liver sections from normal and RILI mice showing radiation-induced histopathological alterations. Scale bars, 100 μm. **(C)** *In vivo* NIR fluorescence images of normal and RILI mice at 5 min to 72 h after Se-Cy4 injection. **(D)** *Ex vivo* NIR fluorescence imaging of major organs collected from normal and RILI mice at corresponding time points. H: Heart, L: Liver, S: Spleen, Lu: Lung, K: Kidney, I: Intestine. **(E-F)** Quantitative fluorescence intensity analysis of Se-Cy4 distribution in the whole body and liver, respectively.

Early detection of RILI is crucial for timely treatment, and developing molecular probes to sensitively detect early oxidative stress and metabolic changes is essential, as conventional imaging and biochemical indicators lack the necessary sensitivity and specificity. Se-Cy4 was selected as the optimal candidate for *in vivo* imaging in both healthy and RILI mice (**Figure 6A-B**). As expected, strong NIR fluorescence signals were predominantly localized in the liver, emerging earlier and more intensely in RILI mice compared with normal controls (**Figure 6C-D**). Consistently, quantitative analysis of fluorescence intensity over time further confirmed this trend, with a pronounced and prolonged fluorescence peak in the RILI group (**Figure 6E-F**). The enhanced fluorescence in RILI mice likely results from two mechanisms: increased hepatic ROS levels from IR that activate Se-Cy4, and impaired metabolic clearance due to hepatic dysfunction, causing delayed excretion and prolonged signal retention. This fluorescence enhancement could serve as an early indicator of liver damage, aiding timely RILI diagnosis and intervention. Once RILI is treated and liver function recovers, the fluorescence pattern will return to normal, helping prevent overuse of antioxidants. Thus, Se-Cy4's imaging ability is valuable for both early diagnosis and therapeutic monitoring of RILI.

### Se-Cy4 exhibits potent systemic and hepatic radioprotection via selenium-dependent antioxidant mechanisms

**Figure 7:**
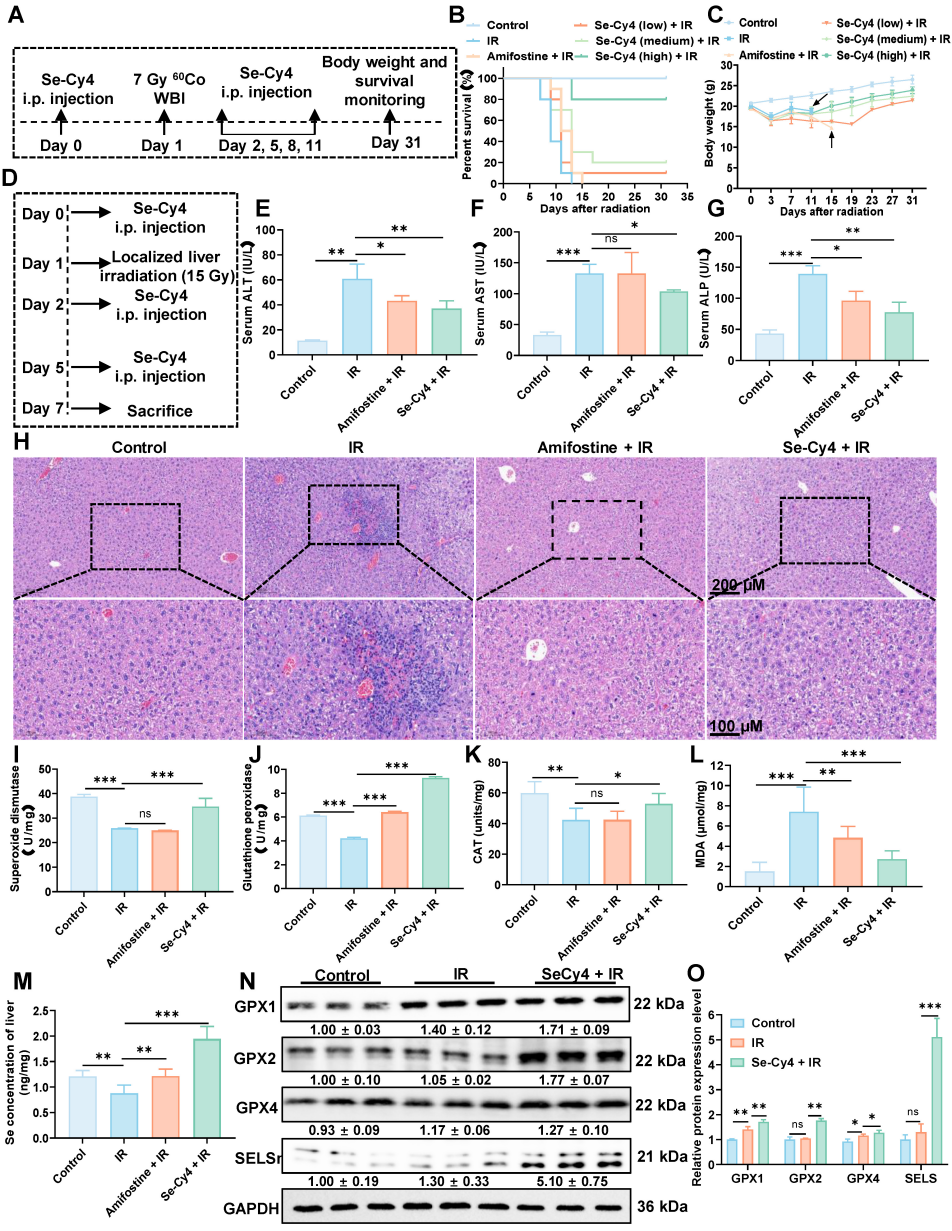
** Se-Cy4 protects against IR–induced systemic injury and liver damage in mice. (A)** Schematic illustration of the experimental design showing Se-Cy4 administration and whole-body IR (7 Gy), followed by survival observation. **(B)** Survival curves of mice after irradiation with or without Se-Cy4 or amifostine treatment (n = 10 per group)**. (C)** Changes in body weight after irradiation with or without Se-Cy4 or amifostine treatment (n = 10 per group)**. (D)** Schematic representation of the experimental design for Se-Cy4 administration and abdominal IR (15 Gy), followed by sample collection and analysis. **(E–G)** Serum levels of ALT, AST, and ALP in each group at 7 days post-radiation (n = 5 per group). **(H)** Representative H&E-stained liver sections showing histopathological alterations in each group; red arrows indicate areas of necrosis and cellular damage. Scale bars, 200 μm (upper) and 100 μm (lower). **(I-M)** Quantification of superoxide dismutase (SOD) activity, glutathione peroxidase (GPx) activity, malondialdehyde (MDA) levels and hepatic selenium content at 7 days after IR (n = 3 per group). **(N)** Western blot analysis of hepatic antioxidant proteins (GPX1, GPX2, GPX4, and SELS) in control, IR, and IR + Se-Cy4 groups. GAPDH served as the loading control.** (O)** Quantification of relative protein expression levels corresponding to panel L. Data are expressed as mean ± SD. Statistical significance was determined by one-way ANOVA followed by Tukey’s post hoc test. *P < 0.05, **P < 0.01, ***P < 0.001.

Next, the *in vivo* radioprotective efficacy of Se-Cy4 was first evaluated in a lethal whole-body IR (7 Gy) mouse model (**Figure 7A**). Survival analysis showed that all mice in the IR group died within nine days, while Se-Cy4 treatment improved survival in a dose-dependent manner. Low- and medium-doses offered limited protection, whereas high-dose Se-Cy4 increased survival to 80% at day 30, comparable to or exceeding amifostine (**Figure 7B**). Additionally, Se-Cy4 effectively reduced IR-induced body weight loss (**Figure 7C**), further confirming its potent radioprotective effects. Encouraged by these systemic protective effects, we investigated organ-level radioprotection using a RILI mouse model with localized abdominal irradiation (**Figure 7D**). Serum biochemical markers of liver injury (ALT, AST, and ALP) were significantly elevated in the IR group, indicating severe hepatic damage. Se-Cy4 treatment markedly reduced these levels, with ALT and ALP returning to near-normal values, and AST levels significantly decreasing (**Figure 7E-G**), demonstrating its strong hepatoprotective effect. Histopathological examination revealed severe hepatic injury in the IR group, including inflammation and necrosis. In contrast, both amifostine and Se-Cy4 treatments alleviated these changes, with Se-Cy4-treated livers showing near-normal architecture similar to controls (**Figure 7H**). Furthermore, immunofluorescence staining of BAX and BCL-2 confirmed that Se-Cy4 effectively protected hepatocytes from IR-induced apoptosis, as evidenced by decreased pro-apoptotic BAX and enhanced anti-apoptotic BCL-2 expression (**Figure S33**). Consistently, TUNEL staining showed that irradiation markedly increased hepatocyte apoptosis in liver tissue, while Se-Cy4 treatment significantly reduced the number of TUNEL-positive cells compared with the IR group. Quantitative analysis demonstrated that the apoptotic rate was significantly reduced by Se-Cy4, to a level comparable with amifostine., supporting the protective effect of Se-Cy4 against RILI (**Figure S34**). Although RILI primarily manifests as hepatic parenchymal and endothelial injury, increasing evidence indicates that RILI can also exert secondary effects on the hematopoietic system through inflammatory signaling, cytokine release, and systemic oxidative stress. To further evaluate whether localized hepatic IR influences hematopoietic homeostasis, peripheral blood parameters were analyzed in the RILI model. Localized abdominal irradiation led to a marked reduction in white blood cell (WBC) and platelet (PLT) counts compared with the control group (**Figure S35**), suggesting that hepatic IR–induced injury and inflammation may contribute to hematopoietic suppression. In contrast, Se-Cy4 treatment significantly alleviated these reductions, restoring WBC and PLT levels toward normal values. Notably, Se-Cy4 exhibited a stronger recovery effect than amifostine, indicating superior protection against irradiation-associated hematopoietic damage. Red blood cell (RBC) counts and hemoglobin (HGB) levels remained largely unchanged among the groups, implying that erythropoiesis was less affected under localized radiation conditions. Collectively, these findings indicate that Se-Cy4 not only protects hepatic tissue from IR injury but also alleviates IR-associated hematopoietic suppression, further supporting its potent systemic radioprotective efficacy in the RILI model.

Since oxidative stress plays a pivotal role in RILI, we next evaluated the hepatic antioxidant defense system by measuring the activities of key antioxidant enzymes, including GPX and SOD. IR exposure resulted in a decrease in the activities of both SOD and GPX compared to the control group, accompanied by a significant elevation in MDA levels (**Figure 7I-L**), indicating significant oxidative damage. In contrast, Se-Cy4 treatment significantly restored the activities of these enzymes, surpassing even those observed in the amifostine-treated group. Notably, Se-Cy4 markedly enhanced GPX activity, even surpassing control levels, suggesting potentiation of the glutathione-dependent antioxidant pathway beyond physiological conditions. Given its stronger effect on GPX than on SOD, we next analyzed the expression of key glutathione peroxidase isoforms (GPX1, GPX2, and GPX4) to clarify the underlying mechanisms. As illustrated in **Figure 7N-O**, Se-Cy4 treatment restored GPX enzymatic activity, with GPX1 and GPX2 showing levels exceeding those in the IR group and approaching or slightly surpassing control levels. GPX4 expression was also moderately increased compared with IR, consistent with the observed enzymatic activity. Considering that selenium is essential for GPX synthesis and that Se-Cy4 was previously shown to undergo de-selenization under oxidative stress, we next investigated its effect on hepatic selenium homeostasis. The IR group exhibited a slight decrease in hepatic Se concentration compared with the control group (**Figure 7M**), suggesting IR-induced selenium depletion. In contrast, Se-Cy4 administration significantly elevated hepatic Se levels—the highest among all groups—indicating efficient selenium delivery and retention in the liver. Because selenoproteins such as SELS require selenium for their synthesis and antioxidant function, we further examined the expression of SELS to assess selenium-dependent biological responses. As shown in **Figure 7N-O**, SELS expression was slightly affected by IR compared with the control group, with no statistically significant decrease. Treatment with Se-Cy4 produced a modest increase in SELS levels, exceeding control levels. Collectively, these findings demonstrate that Se-Cy4 replenishes hepatic selenium, enhances GPX activity, and upregulates SELS expression, jointly reinforcing selenium-dependent antioxidant defenses against RILI.

### Nrf2 activation is required for Se-Cy4–induced antioxidant enhancement and radioprotection

**Figure 8:**
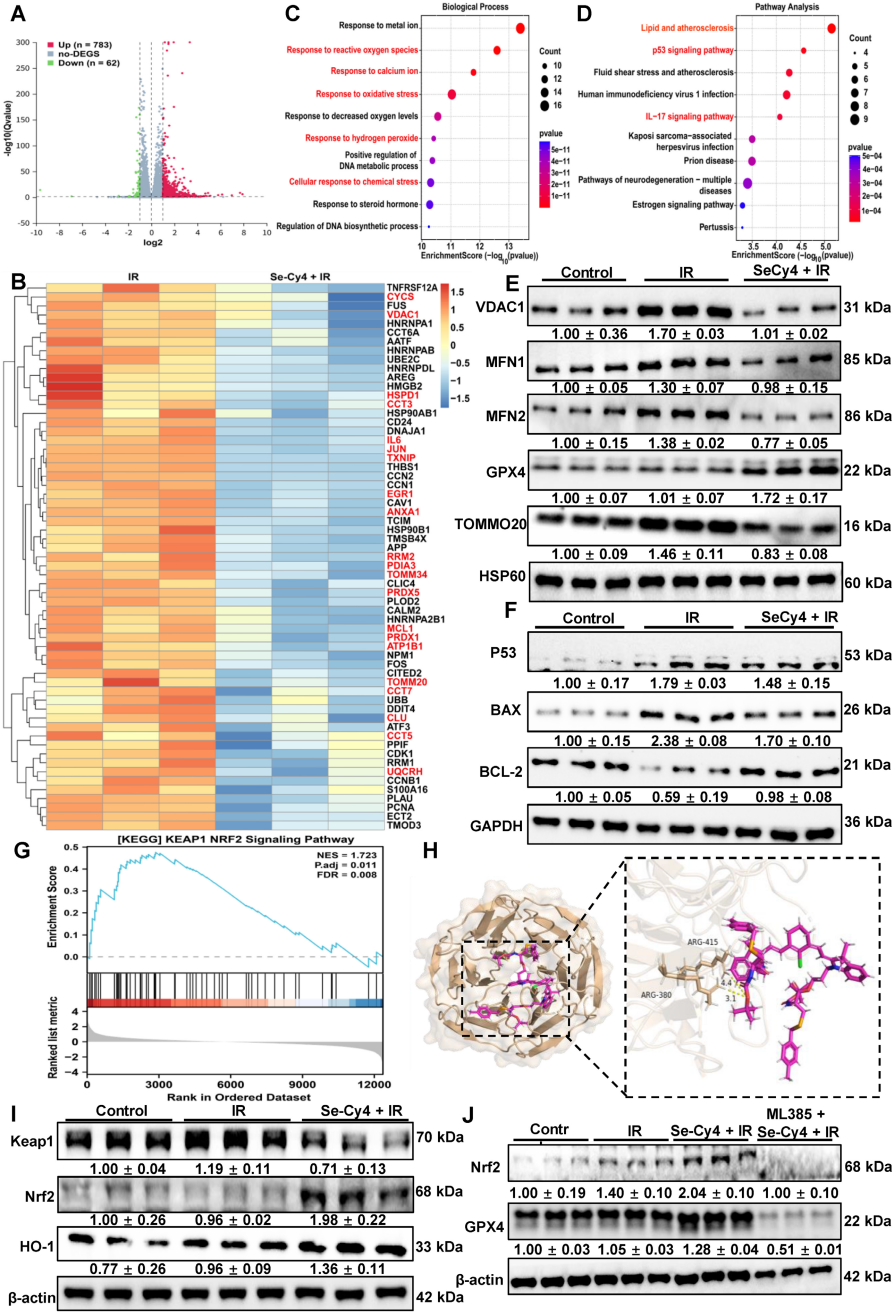
** Se-Cy4 maintains mitochondrial homeostasis and activates Keap1–Nrf2 signaling to mitigate irradiation-induced oxidative injury. (a)** Volcano plot illustrating differentially expressed genes (DEGs) between the IR and Se-Cy4 + IR groups.** (b)** Heatmap of representative DEGs associated with mitochondrial structure, redox regulation, and apoptosis, comparing expression patterns across treatment groups. **(c)** Gene Ontology (GO) biological process enrichment analysis of DEGs, showing major biological pathways affected by IR and Se-Cy4 treatment.** (d)** KEGG pathway enrichment analysis highlighting the primary signaling pathways associated with the identified DEGs.** (e)** Western blot analysis of key mitochondrial homeostasis–related proteins, including VDAC1, MFN1, MFN2, GPX4, TOMM20 in each group. HSP60 served as the loading control.** (f)** Western blot analysis of apoptosis-associated proteins BAX and BCL-2 in the indicated groups.** (g)** Gene set enrichment analysis (GSEA) plot showing enrichment of the KEAP1–NRF2 signaling pathway in Se-Cy4 + IR samples compared with IR samples.** (h)** Molecular docking model illustrating the interaction between Se-Cy4 and the Keap1 protein.** (i)** Western blot analysis of Keap1, Nrf2, and HO-1 expression across treatment groups, with β-actin as the loading control.** (j)** Western blot analysis of Nrf2 and GPX4 expression in control, IR, Se-Cy4 + IR, and ML385 + Se-Cy4 + IR groups.

Building on the *in vivo* findings, transcriptomic analysis was performed to further elucidate the molecular mechanisms underlying the radioprotective effects of Se-Cy4. Differential gene expression analysis revealed pronounced transcriptional changes between the IR and Se-Cy4 + IR groups, with 783 genes upregulated and 62 downregulated following Se-Cy4 treatment (**Figure 8A**). Notably, several key genes highlighted in the heatmap (**Figure 8B**)—such as *CYCS*, *TXNIP*, *HSPD1*, *PRDX1*, *VDAC1*, and *TOMM20*—are closely associated with mitochondrial structure and function. Among them, *CYCS* (cytochrome c) and *VDAC1* (voltage-dependent anion channel 1) are crucial regulators of mitochondrial outer membrane permeability and apoptosis, whereas *HSPD1* (HSP60) and *TOMM20* participate in mitochondrial protein folding and import. In addition, *TXNIP* and *PRDX1* are central modulators of redox balance within the mitochondrial matrix. Se-Cy4 treatment reversed the radiation-induced dysregulation of these mitochondrial genes, restoring expression patterns toward normal levels. This transcriptional reprogramming suggests that Se-Cy4 preserves mitochondrial integrity, mitigates oxidative stress, and stabilizes energy metabolism in irradiated hepatic tissue. GO enrichment analysis further indicated that the differentially expressed genes were mainly involved in oxidative stress–related biological processes, including *response to reactive oxygen species*, *response to oxidative stress*, and *response to decreased oxygen levels* (**Figure 8C**). Consistently, pathway enrichment analysis revealed several signaling pathways modulated by Se-Cy4, such as the *p53 signaling pathway*, *IL-17 signaling pathway*, and pathways related to *lipid metabolism* and *atherosclerosis*, all of which are tightly linked to oxidative stress regulation, DNA repair, and inflammatory responses (**Figure 8D**). Overall, these results indicate that Se-Cy4 protects against RILI by maintaining mitochondrial homeostasis and regulating oxidative stress–associated signaling cascades.

To validate the transcriptomic findings and further elucidate the protective effects of Se-Cy4 on mitochondrial homeostasis, we examined the expression of several key mitochondrial proteins, including VDAC1, MFN1, MFN2, GPX4, and TOMM20 (**Figure 8E and Figure S36A-E**). Compared with the control group, IR exposure modestly increased VDAC1 levels and led to a slight reduction in MFN1, MFN2, and TOMM20, suggesting mild perturbation of mitochondrial membrane integrity, fusion dynamics, and protein import. GPX4 expression was notably upregulated in the IR group, potentially reflecting a compensatory response to oxidative stress. In contrast, Se-Cy4 treatment largely reversed these IR-induced alterations. In the Se-Cy4 + IR group, VDAC1 levels returned toward control, MFN1 and MFN2 expression was restored, GPX4 levels increased, and TOMM20 expression recovered. These results indicate that Se-Cy4 contributes to the maintenance of mitochondrial structure and function under IR-induced stress, supporting its role in preserving mitochondrial homeostasis.

Given that IR-induced mitochondrial dysfunction activates intrinsic apoptosis, we examined key apoptosis-related proteins to see if Se-Cy4 modulates this pathway. IR increased pro-apoptotic BAX and decreased anti-apoptotic BCL-2 compared to controls. Se-Cy4 treatment reversed these changes, reducing BAX expression and restoring BCL-2 levels, shifting the BAX/BCL-2 balance toward cell survival (**Figure 8F and Figure S36F-H**). Collectively, these protein expression changes are consistent with the transcriptomic results and further demonstrate that Se-Cy4 mitigates RILI by maintaining mitochondrial homeostasis and preventing the activation of mitochondrial-dependent apoptotic pathways.

Building on the observation that Se-Cy4 predominantly affects oxidative stress–related pathways, we further explored whether key antioxidant regulatory mechanisms were also involved. GSEA analysis revealed a significant enrichment of the Keap1–Nrf2 signaling pathway (NES = 1.723, FDR = 0.008), with Nrf2-responsive genes clustered toward the upregulated end of the ranked list (**Figure 8G**), indicating that Se-Cy4 activates the Keap1–Nrf2 axis, a master regulator of cytoprotective and antioxidant responses. Under physiological conditions, Nrf2 remains sequestered in the cytoplasm through its interaction with Keap1. To investigate whether Se-Cy4 directly interferes with this interaction, molecular docking analysis was performed (**Figure 8H**). The results revealed that Se-Cy4 specifically binds to the critical Keap1 residues Arg380 and Arg415, which are essential for maintaining the structural stability of the Keap1–Nrf2 complex. Se-Cy4 engages key binding sites on Keap1, thereby weakening the Keap1–Nrf2 interaction and promoting Nrf2 activation. Consistently, immunofluorescence revealed that Se-Cy4 restored IR-impaired nuclear Nrf2 accumulation, indicative of reactivated Nrf2-dependent antioxidant signaling. Compared with the IR group, cells treated with Se-Cy4 displayed a pronounced increase in nuclear Nrf2 fluorescence intensity, accompanied by a reduction in cytoplasmic retention, further supporting the relief of Keap1-mediated repression (**Figure S37**). Similarly, Western blot analysis both *in vitro* and *in vivo* demonstrated a substantial decrease in Keap1 protein levels, accompanied by enhanced stabilization of Nrf2 and markedly elevated expression of its downstream effector HO-1 in the Se-Cy4 + IR group relative to IR alone (**Figure 8I**, **Figure S38 and Figure S39**). The coordinated upregulation of Nrf2 and HO-1 indicates that Se-Cy4 not only disrupts the Keap1–Nrf2 complex but also robustly amplifies the downstream transcriptional antioxidant program. These findings further support the conclusion that Se-Cy4 reinforces cellular redox homeostasis by promoting NRF2 activation across multiple biological contexts. Notably, the selenium-related antioxidant enzymes GPX1, GPX2, GPX4, and SelS have been reported as direct or indirect transcriptional targets of Nrf2. Therefore, we further examined their expression at the cellular level. Consistent with the *in vivo* findings, the Se-Cy4 + IR group exhibited substantially increased GPX1 and GPX2 protein levels, while GPX4 expression remained stable and showed a further upward trend, and SELS levels were notably restored (**Figure S40**). This expression pattern closely parallels the activation of the Keap1–Nrf2 pathway. To further investigate the involvement of the Keap1–Nrf2 axis in the radioprotective effects of Se-Cy4, we assessed the impact of the Nrf2-specific inhibitor ML385 on Se-Cy4–mediated cytoprotection. CCK-8 analysis revealed that the enhanced cell viability conferred by Se-Cy4 following IR exposure was markedly diminished upon ML385 treatment, indicating that inhibition of Nrf2 largely abolishes the protective effect of Se-Cy4 (**Figure S41A**). Consistently, dead staining further demonstrated a pronounced increase in PI-positive (dead) cells in the ML385 + Se-Cy4 + IR group compared with the Se-Cy4 + IR group, whereas Se-Cy4 alone substantially reduced IR-induced cell death to near-baseline levels (**Figure S41B-C**). The reappearance of extensive cell death upon Nrf2 blockade strongly suggests that Nrf2 activation is indispensable for the radioprotective function of Se-Cy4. We next evaluated whether ML385 also affected the Se-Cy4–induced upregulation of Nrf2 and its downstream antioxidant enzyme GPX4. Western blot analysis showed that Se-Cy4 significantly increased Nrf2 protein stabilization following IR, whereas ML385 treatment effectively suppressed this upregulation (**Figure 8J**). A similar pattern was observed for GPX4: Se-Cy4 restored and further enhanced GPX4 expression after IR, while ML385 markedly attenuated this increase (**Figure 8J and Figure S42**). Immunofluorescence staining corroborated the biochemical results. Se-Cy4 promoted robust nuclear accumulation of Nrf2 and restored GPX4 expression at the cellular level, however, ML385 substantially impeded Nrf2 nuclear translocation and diminished GPX4 fluorescence intensity, thereby reversing the Se-Cy4–mediated antioxidant response (**Figure S43-S44**). Together, these results demonstrate that Se-Cy4 relies on Nrf2 activation to elevate downstream antioxidant defenses, including GPX4, and that pharmacological inhibition of Nrf2 effectively negates both the signaling activation and the cytoprotective effects of Se-Cy4.

### Biosafety evaluation of Se-Cy4

Given the potent radioprotective efficacy of Se-Cy4 demonstrated above, we next systematically evaluated its biosafety to ensure suitability for potential therapeutic applications. As shown in **Figure S45**, hemolysis assays confirmed excellent blood compatibility, with hemolysis rates remaining below 2% across all tested concentrations—well within the ISO 10993-5 safety threshold (<5%). The *in vivo* biocompatibility of Se-Cy4 was further examined in healthy BALB/c mice. Following repeated intravenous administrations (20 mg·kg⁻¹, once every three days for a total of five doses), histological analysis of major organs on day 31 revealed no detectable pathological abnormalities in the heart, liver, spleen, lung, or kidney compared with the control group (**Figure S46**). These findings collectively demonstrate the excellent systemic safety and biocompatibility of Se-Cy4, supporting its feasibility for further* in vivo* radioprotective and translational investigations.

## Discussion

RILI remains a major challenge in nuclear-related exposure and radiotherapy because of the lack of organ-targeted radioprotectants, reliable early detection tools, and effective therapeutic strategies 39. Existing radioprotectors suffer from several limitations, including poor selectivity for irradiated tissues, a reliance on nonspecific ROS scavenging with narrow therapeutic windows, and the absence of sensitive modalities for early diagnosis 40-42. These shortcomings hinder timely intervention and can lead to excessive antioxidation and drug-related toxicity. The development of liver-selective contrast agents that enable noninvasive, real-time monitoring of RILI progression and therapeutic response would therefore be highly advantageous, enabling more precise treatment while minimizing adverse effects 43, 44.

In this context, we present Se-Cy4, a mitochondria-targeted, selenium-releasing heptamethine cyanine platform that integrates liver-selective accumulation, ROS-responsive NIR imaging, and radioprotection. Upon exposure to ROS, Se-Cy4 undergoes deselenization, releasing bioavailable selenium and generating an amplifying NIR fluorescence signal that reports oxidative stress in real time. Functionally, Se-Cy4 mitigates radiation-induced oxidative and mitochondrial damage, preserves mitochondrial bioenergetics, and improves survival in RILI models. Mechanistically, Se-Cy4 activates the Keap1–Nrf2 pathway, promotes the expression of key selenoproteins, and consequently strengthens intrinsic antioxidant defense systems. Unlike conventional radioprotectants, Se-Cy4 specifically targets liver mitochondria and simultaneously provides imaging and therapeutic functions, offering a dynamic monitoring approach during RILI management.

Early diagnosis is crucial for effective intervention and management of RILI, however, the lack of reliable early biomarkers continues to impede timely detection 45. Clinically, the diagnosis of RILI still relies mainly on its temporal association with radiotherapy, deterioration of liver function, biochemical abnormalities, and radiologic findings. Classic RILI is typically characterized by anicteric hepatomegaly, ascites, and elevated alkaline phosphatase, whereas non-classic RILI is more often defined by marked transaminase elevation and/or worsening of Child-Pugh score 46. However, these criteria largely reflect downstream functional impairment rather than early molecular events, and in clinical practice they may be confounded by tumor progression, portal vein tumor thrombus, viral reactivation, or pre-existing cirrhosis 47. Recent advances include the use of more objective liver reserve indicators, such as changes in the albumin-bilirubin score, and functional imaging approaches based on gadoxetic acid-enhanced MRI for assessing radiation-related liver injury 48, 49. Nevertheless, clinically applicable early and specific biomarkers for RILI remain lacking.

In this context, the theranostic strategy developed in our work may offer several advantages. First, it responds to oxidative stress, a key upstream event in RILI pathogenesis, enabling molecular-level visualization rather than relying solely on delayed structural or biochemical changes. Second, it integrates imaging and intervention into a single platform, allowing diagnosis and treatment to be more closely coupled. Similar integrated strategies have been reported in other liver injury settings, including hepatic ischemia-reperfusion injury and drug-induced liver injury 50, 51. Third, compared with these previously reported systems, our design is tailored to the irradiation-associated oxidative microenvironment and may therefore be more suitable for studying and potentially managing RILI. Collectively, these findings suggest that ROS-responsive theranostic agents may complement current clinical assessment and provide a promising strategy for earlier detection and intervention in radiation-related liver injury.

Mitochondria, as central regulators of cellular energy metabolism and signal transduction, are key targets in the pathogenesis of RILI 52. Owing to their high metabolic activity and substantial energy demands, hepatocytes are densely populated with mitochondria, rendering them particularly vulnerable to IR 53-55. IR exposure can directly or indirectly damage mtDNA and induce excessive generation of ROS, leading to lipid peroxidation, dissipation of MMP, respiratory chain dysfunction, ATP depletion, and aberrant opening of the mitochondrial permeability transition pore (mPTP) 6. These pathological alterations compromise cellular bioenergetics, activate mitochondria-dependent apoptotic or necrotic pathways, and elicit inflammatory responses, ultimately exacerbating hepatic injury and fibrosis 56, 57. Consequently, the preservation of mitochondrial structure and function has emerged as a promising therapeutic strategy for the prevention and treatment of RILI. Se, as an essential micronutrient, is uniquely positioned at the intersection of redox control and mitochondrial health through its incorporation into selenoproteins such as GPXs and other stress-responsive regulators 58, 59. However, the clinical utility of Se supplementation has been largely constrained by limited tissue selectivity and insufficient subcellular precision. Here, we introduce Se-Cy4 as a liver- and mitochondria-enriched Se carrier that couples targeted biodistribution with organelle-resolved cytoprotection, thereby overcoming a key translational bottleneck of selenium-based interventions. Mechanistically, this design is poised to disrupt the pathogenic feed-forward cycle linking mitochondrial oxidative injury to impaired antioxidant capacity by concurrently preserving mitochondrial integrity and reinforcing Se-dependent redox defenses. Collectively, these findings position mitochondria-targeted Se delivery as a conceptually distinct and underexplored therapeutic paradigm for RILI, enabling redox reprogramming and bioenergetic preservation while attenuating downstream inflammatory and fibrotic progression.

Nrf2 is a critical transcription factor that regulates the expression of downstream antioxidant genes by activating antioxidant response elements (ARE), orchestrating the cellular defense against oxidative stress 60. Under normal conditions, Nrf2 is sequestered in the cytoplasm through its interaction with Keap1, maintaining low intracellular levels of Nrf2 61. Recent studies underscore the importance of Nrf2 activation as a key adaptive response to IR-induced oxidative damage. For instance, in pulmonary and hepatic radiation models, Nrf2 activation has been shown to protect against cell death and inflammation, while Nrf2 deficiency or inhibition exacerbates tissue damage 62, 63. In this study, we identify Se-Cy4 as a potent organoselenium-based activator of the Keap1–Nrf2 signaling pathway. Molecular docking analysis revealed that Se-Cy4 specifically binds to critical residues in Keap1 (Arg380 and Arg415), disrupting the Keap1–Nrf2 interaction and facilitating Nrf2 release. Importantly, inhibition of Nrf2 with ML385 significantly abolished Se-Cy4–mediated cytoprotection. Nrf2 enhances the expression of downstream target genes, including HO-1, GPX4, GCLC, and NQO1, which play key roles in antioxidant defense and detoxification. This upregulation helps to scavenge intracellular ROS and reduce lipid peroxidation, thereby mitigating cellular damage 64, 65. Consistent with these findings, our results demonstrated that Se-Cy4 enhanced Nrf2 nuclear accumulation and upregulated the expression of downstream antioxidant genes, including HO-1 and selenium-related enzymes like GPX1, GPX2, GPX4, and SELS.

Although Se-Cy4 demonstrates strong radioprotective and imaging performance, several limitations need addressing. First, the study focused on acute RILI, with long-term safety, pharmacokinetics, and therapeutic efficacy under chronic or fractionated radiation exposure, as well as repeated dosing, yet to be evaluated. Second, its protective effects in other radiation-sensitive organs, such as bone marrow, intestine, and lymphatic system, remain unexplored. Finally, while docking and inhibition data suggest Keap1–Nrf2 modulation, further structural and biochemical characterization of Se-Cy4–Keap1 interactions is needed to clarify the molecular mechanism. Despite these limitations, our findings present Se-Cy4 as a promising liver-directed theranostic candidate for RILI, warranting further preclinical optimization and translational investigation.

## Conclusion

In this work, we designed and developed several new selenium-substituted heptamethine cyanine derivatives as theranostic radioprotectors for targeted imaging and therapy in RILI. The Se-Cys derivatives were synthesized by tuning their physicochemical properties, such as lipophilicity, ROS reactivity, and fluorescence performance. Among these derivatives, Se-Cy4 was identified for its excellent mitochondria-targeting accumulation and radioprotective properties. Notably, Se-Cy4 exhibits ROS-responsive bond cleavage, deselenization, and fluorescence enhancement in irradiated liver mitochondria. These features make Se-Cy4 an ideal candidate for RILI-targeted imaging and treatment, as a high-ROS microenvironment is characteristic of injured hepatic tissues and mitochondria. The intrinsic NIR fluorescence of Se-Cy4 enables noninvasive, real-time monitoring of RILI progression and therapeutic response. Additionally, its selenium release activates the Keap1–Nrf2 pathway, enhances antioxidant defense, stabilizes mitochondria, and upregulates key selenoproteins, collectively reducing oxidative stress, inflammation, and apoptosis. Overall, Se-Cy4 represents a first-in-class mitochondria-targeted selenium theranostic that combines both diagnostic and therapeutic functions in a single molecular platform. This work establishes a generalizable strategy for redox-responsive imaging and radioprotection, with strong translational potential for clinical applications in radioprotection, radiation oncology, and oxidative stress–related diseases.

## Supplementary Material

Supplementary methods, figures and table.

## Figures and Tables

**Scheme 1:**
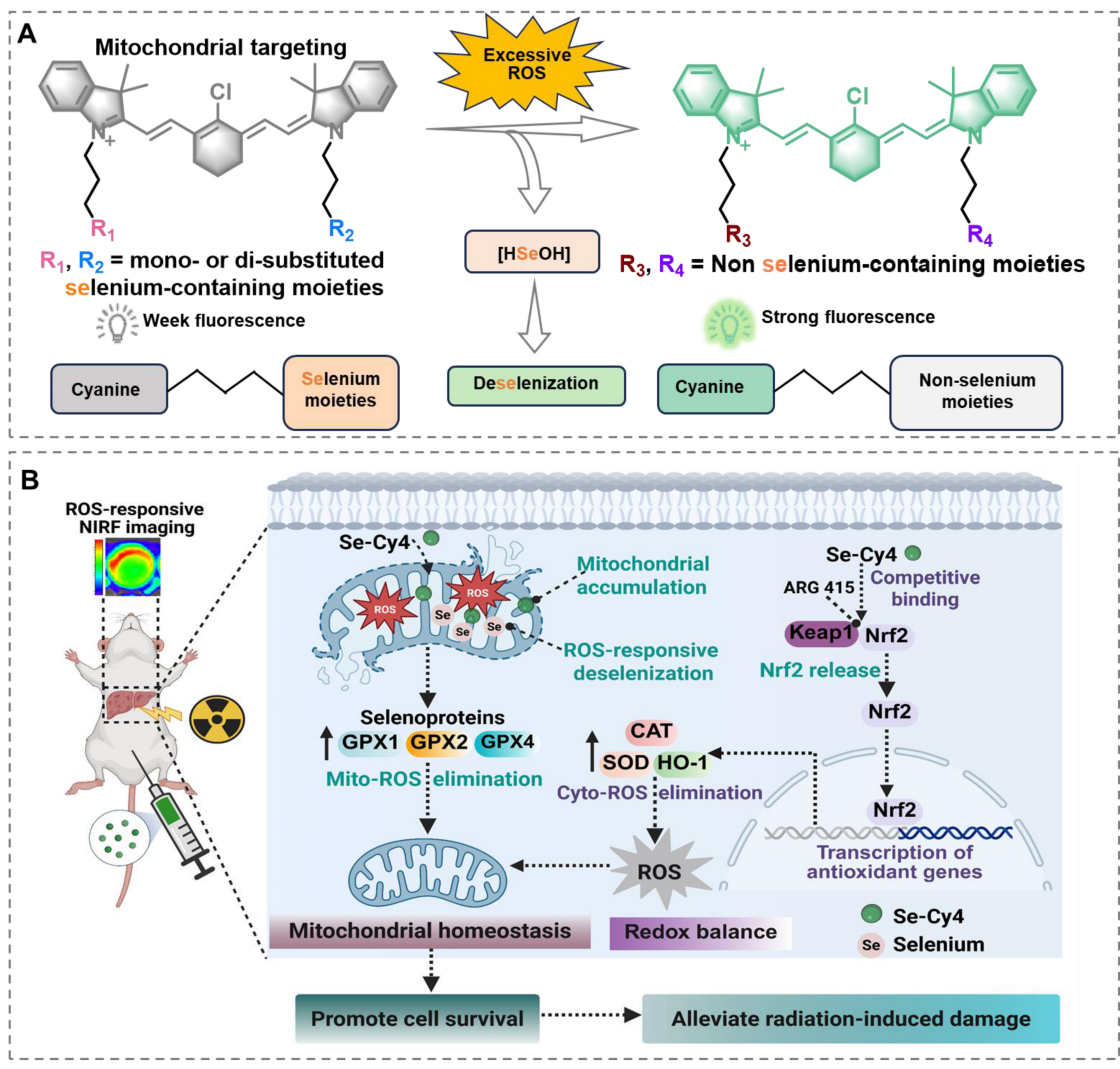
** Schematic illustration of Se-Cys derivatives as mitochondria-targeted theranostic radioprotectors.** Excessive ROS triggers deselenization of selenium-substituted heptamethine cyanine derivatives (Se-Cys), converting weakly fluorescent selenium-containing structures into strongly fluorescent non-selenium cyanines and releasing bioavailable selenium, while Se-Cy4 accumulates in mitochondria, enhances selenoprotein-mediated antioxidant defense, preserves mitochondrial redox homeostasis and activates the Keap1–Nrf2 pathway to mitigate radiation-induced liver injury.

## Data Availability

The data that support the findings of this study are available on request from the corresponding author.
