# Supplementary material for: Mitochondria-targeting organoselenium theranostic radioprotectors for simultaneous treatment and imaging of radiation-induced liver injury
Source: Theranostics. 2026 May 1;16(12):6554–75. doi: 10.7150/thno.133714 (PMC13231995; doi:10.7150/thno.133714)
Supplement: Supplementary file 1 — Supplementary methods, figures and table. [file thnov16p6554s1.pdf]

Supporting Information

Mitochondria-targeting organoselenium theranostic radioprotectors for simultaneous treatment and imaging of radiation-induced liver injury

Zifei Wu [a,1], Xie Huang [a,1], Mingquan Gao [a,1], Liting Wang [c], Zaizhi Du [a], Ziqian Shang [a], Xudong Yu [a], Xiaojiao Wang [a], Shuyue Deng [a], Xinrui Yang [a], Binghui Lu [a], Jing Liu [a], Weidong Wang [b,*], Rong Li [a,*], Shenglin Luo [a,*]

[a] Institute of Combined Injury, State Key Laboratory of Trauma and Chemical Poisoning, Chongqing Engineering Research Center for Nanomedicine, Department of Military Preventive Medicine, Third Military Medical University (Army Medical University), 30 Gaotanyan street, Chongqing 400038, China.

[b] Department of Radiation Oncology, Radiation Oncology Key Laboratory of Sichuan Province, Sichuan Clinical Research Center for Cancer, Sichuan Cancer Hospital & Institute, Affiliated Cancer Hospital of University of Electronic Science and Technology of China, Chengdu 610000, China.

[c] Biomedical Analysis Center, Third Military Medical University (Army Medical University), 30 Gaotanyan street, Chongqing, 400038, China.

[1] These authors contribute equally to this work: Zifei Wu, Xie Huang, Mingquan Gao.

[*] Corresponding authors: Shenglin Luo, Rong Li, Weidong Wang.

E-mail: luosl@tmmu.edu.cn; lirongyl@tmmu.edu.cn; wwdwyl@uestc.edu.cn.

Materials and Methods

Synthesis of intermediate 1a

2,3,3-Trimethylindolenine (40.32 mmol) and 3-iodopropanol (26.88 mmol) were dissolved in MeCN (20 mL) in a 100 mL round-bottom flask. The mixture was heated to 110 °C and stirred for 16 h under a nitrogen atmosphere. The solvent was then removed under reduced pressure, and the residue was washed with 20 mL of a DCM/EA mixture (1:5, v/v), giving a solid. The solid was collected by filtration and dried under vacuum to afford crude **1a**, an indolium quaternary ammonium salt, which was used directly in the subsequent reaction without further purification.

Synthesis of cyanine dye Se-Cy0

Crude **1a** (5.2 mmol) and compound **2a** (2.08 mmol) were dissolved in a mixture of toluene and 1-butanol (7:3, v/v; 20 mL) in a 100 mL round-bottom flask. The reaction mixture was heated at 110 °C and stirred for 12 h under a nitrogen atmosphere. The solvent was then removed under reduced pressure, and the residue was purified by column chromatography on silica gel to afford **Se-Cy0** as a green solid powder (56.7% yield).

Se-Cy0: ^{1}H NMR (400 MHz, $CDCl_3$) δ 8.33 (d, J = 14.0 Hz, 2H), 7.40 – 7.35 (m, 4H), 7.24 – 7.20 (m, 4H), 6.37 (d, J = 14.0 Hz, 2H), 4.29 (t, J = 6.8 Hz, 4H), 3.79 (t, J = 5.2 Hz, 4H), 2.77 – 2.71 (m, 4H), 2.11 – 2.08 (m, 4H), 2.00 – 1.93 (m, 4H), 1.70 (s, 12H) ppm.

Synthesis of Se-Cys derivatives

Cyanine dye **Se-Cy0** (0.269 mmol) and a selenium-containing compound (0.269 mmol, compound **3a**, compound **4a**, or compound **5a**) were dissolved in 10 mL DCM. The mixture was stirred at 0 °C for 30 min under a nitrogen atmosphere. Then DCC (0.807 mmol) and DMAP (0.807 mmol) were added to above reaction mixture. After an additional 30 min, the reaction mixture was returned to room temperature and stirred

overnight. Then the reaction solvent was evaporated in vacuum and the obtained residue was purified by column chromatography on silica gel (DCM : MeOH = 15:1, v/v) to afford the desired Se-Cys derivatives.

Se-Cy1 (green solid, 39.2% yield): ^{1}H NMR (400 MHz, $CDCl_3$) δ 8.38 – 8.27 (m, 2H), 7.43 – 7.38 (m, 4H), 7.29 – 7.27 (m, 4H), 6.40 – 6.32 (m, 2H), 4.44 – 4.38 (m, 2H), 4.28 – 4.18 (m, 4H), 3.48 – 3.38 (m, 2H), 3.20 – 3.06 (m, 4H), 2.93 (t, *J* = 7.2 Hz, 2H), 2.81 – 2.76 (m, 4H), 2.24 – 2.20 (m, 4H), 2.10 – 1.91 (m, 4H), 1.73 (s, 12H) ppm. HRMS $[M - I]^+$ calcd for $C_{42}H_{50}ClN_2O_4Se_2^+$, 841.1784; found, 841.1772.

Se-Cy2 (green solid, 14.0% yield): ^{1}H NMR (400 MHz, $CDCl_3$) δ 8.33 (d, *J* = 13.6 Hz, 2H), 7.74 (d, *J* = 8.4 Hz, 4H), 7.61 (d, *J* = 7.2 Hz, 4H), 7.40 – 7.28 (m, 13H), 7.21 – 7.18 (m, 4H), 6.31 (d, *J* = 14.0 Hz, 1H), 4.50 – 4.29 (m, 14H), 4.23 (t, *J* = 6.8 Hz, 2H), 2.84 – 2.77 (m, 4H), 2.62 – 2.55 (m, 4H), 2.26 – 2.21 (m, 6H), 2.02 – 1.92 (m, 12H), 1.71 (m, 12H) ppm. HRMS $[M - I]^+$ calcd for $C_{76}H_{82}ClN_4O_8Se_2^+$, 1373.4146; found, 1373.4122.

Se-Cy3 (green solid, 28.4% yield): ^{1}H NMR (400 MHz, $CDCl_3$) δ 8.46 (d, *J* = 14.4 Hz, 1H), 8.18 (d, *J* = 13.6 Hz, 1H), 7.75 (d, *J* = 7.2 Hz, 2H), 7.60 (d, *J* = 7.6 Hz, 2H), 7.46 – 7.29 (m, 10H), 7.13 (t, *J* = 7.2 Hz, 1H), 7.02 (d, *J* = 8.0 Hz, 1H), 6.73 (d, *J* = 14.8 Hz, 1H), 5.97 (d, *J* = 13.2 Hz, 1H), 4.52 – 4.33 (m, 6H), 4.27 – 4.21 (m, 2H), 4.12 – 4.07 (m, 2H), 3.83 – 3.80 (m, 2H), 2.84 – 2.81 (m, 2H), 2.68 (t, *J* = 6.8 Hz, 2H), 2.61 – 2.56 (m, 2H), 2.25 – 2.09 (m, 6H), 2.02 (s, 3H), 1.98 – 1.94 (m, 3H), 1.78 – 1.63 (m, 15H) ppm. HRMS $[M - I]^+$ calcd for $C_{56}H_{63}ClN_3O_5Se^+$, 972.3616; found, 972.3604.

Se-Cy4 (green solid, 29.0% yield): ^{1}H NMR (400 MHz, $CDCl_3$) δ 8.34 (d, *J* = 14.0 Hz, 2H), 7.40 – 7.36 (m, 4H), 7.24 – 7.16 (m, 8H), 7.08 (d, *J* = 8.0 Hz, 4H), 6.34 (d, *J* = 14.0 Hz, 2H), 4.39 – 4.31 (m, 8H), 3.80 (s, 4H), 2.90 – 2.88 (m, 3H), 2.81 – 2.78 (m, 3H), 2.29 (s, 6H), 2.26 – 2.20 (m, 4H), 2.04 – 1.96 (m, 4H), 1.72 (s, 12H), 1.65 – 1.60 (m, 6H), 1.46 (s, 18H) ppm. HRMS $[M - I]^+$ calcd for $C_{68}H_{86}ClN_4O_8Se_2^+$, 1281.4459; found, 1281.4464.

Se-Cy5 (green solid, 28.6% yield): ^{1}H NMR (400 MHz, $CDCl_3$) δ 8.48 (d, *J* = 14.4 Hz,

1H), 8.18 (d, J = 13.2 Hz, 1H), 7.48 – 7.30 (m, 6H), 7.18 – 7.07 (m, 5H), 7.01 (d, J = 8.0 Hz, 1H), 6.77 (d, J = 14.8 Hz, 1H), 5.93 (d, J = 13.6 Hz, 1H), 4.54 (t, J = 7.2 Hz, 3H), 4.29 – 4.25 (m, 2H), 4.05 (t, J = 6.4 Hz, 2H), 3.84 – 3.80 (m, 5H), 2.90 (d, J = 5.6 Hz, 2H), 2.84 (t, J = 6.0 Hz, 2H), 2.66 (t, J = 6.0 Hz, 2H), 2.29 (s, 3H), 2.20 – 2.14 (m, 4H), 2.00 – 1.95 (m, 2H), 1.74 (s, 6H), 1.70 (s, 6H), 1.46 (s, 9H) ppm. HRMS $[M - I]^+$ calcd for $C_{52}H_{65}ClN_3O_5Se^+$, 926.3772; found, 926.3768.

Optical properties characterization

Se-Cys derivatives (Se-Cy1, Se-Cy2, Se-Cy3, Se-Cy4, and Se-Cy5) were first dissolved in dimethyl sulfoxide (DMSO) to prepare 10 mM stock solutions. For spectral measurements, 3 μL of each stock solution was diluted in 3 mL of methanol or PBS to a final concentration of 10 μM. UV–vis absorption spectra and fluorescence emission spectra were recorded using an L9 double-beam UV–vis spectrophotometer (Analytical Instrument, Shanghai, China) and an F380 fluorescence spectrometer (Gangdong Sci & Tech, Tianjin, China), respectively.

For anti-interference fluorescence spectrometry detection, various amino acids and metal ions were prepared as 10 mM stock solutions in ultrapure water. A 10 mM Se-Cy4 stock solution was diluted with PBS buffer (pH 7.4, 3 mL per tube) in 17 centrifuge tubes to obtain a final concentration of 10 μM. Subsequently, cystine (Cyss), arginine (Arg), kynurenine (Kyn), N-acetylcysteine (NAC), glycine (Gly), Mg^{2+}, Mn^{2+}, Na^+, Zn^{2+}, Al^{3+}, Ca^{2+}, Cu^{2+}, Fe^{2+}, Fe^{3+}, K^+, and H_2O_2 were individually added to 16 tubes to achieve a final concentration of 200 μM, while one tube without any addition served as the control. After incubation at 37 °C for 10 min under shaking, the fluorescence emission spectra were recorded using a fluorescence spectrometer.

DFT and quantum chemical parameters calculation

Density functional theory (DFT) calculations were performed using the B3LYP functional combined with the D3BJ dispersion correction [1]. Geometry optimizations were conducted with the 6-31G(d,p) basis set. All structures were fully optimized in a mixed solvent system (water : ethanol = 8 : 2) using the SMD solvation model, without

applying any structural constraints. Harmonic frequency analyses were carried out at the same level of theory to confirm that no imaginary frequencies were present. Single-point energies were calculated at the B3LYP-D3BJ/6-311+G(d,p) level. Frontier molecular orbitals were visualized using the Vesta program[2] with the assistance of the Multiwfn package[3]. All computations were performed using the Gaussian 16 software.

Based on DFT calculation results, a series of quantum chemical parameters of Se-Cy4 and IM1 – IM6 were calculated by following equations [4, 5] :

Energy gap between E_{LUMO} and E_{HOMO} ($\Delta E_{\text{LUMO-HOMO}}$):

$$\Delta E_{\text{LUMO-HOMO}} = E_{\text{LUMO}} - E_{\text{HOMO}}$$

Electronegativity (χ):

$$\chi = \frac{-(E_{\text{LUMO}} + E_{\text{HOMO}})}{2}$$

Absolute hardness (η):

$$\eta = \frac{E_{\text{LUMO}} - E_{\text{HOMO}}}{2}$$

Chemical potential (CP):

$$CP = -\chi$$

Electrophilicity index (ω):

$$\omega = \frac{(CP)^2}{2\eta}$$

Nucleophilicity index (N):

$$N = \frac{1}{\omega}$$

Cell culture

The human normal hepatocyte cell line L-02 was obtained from Beijing Liweining Biotechnology Co., Ltd. (China) and cultured in Roswell Park Memorial Institute 1640 medium (RPMI-1640) supplemented with 10% fetal bovine serum (FBS; Gibco, USA) and 1% penicillin–streptomycin solution (HyClone, USA). Cells were maintained at 37 °C in a humidified incubator with 5% CO_2. Cells in the logarithmic growth phase with healthy morphology were used for subsequent experiments.

EdU cell proliferation assay

Cell proliferation was assessed using the EdU Cell Proliferation Kit (Beyotime Biotechnology, China). L-02 cells treated with 2.5 μM Se-Cy2, Se-Cy3, Se-Cy4 and Se-Cy5 or 10 μM Amifostine were cultured in 6-well plates in the presence of EdU reagent for the 24h. After incubation, cells were fixed with 4% paraformaldehyde and permeabilized with 0.5% Triton X-100 (Sigma-Aldrich, USA). Nuclei were counterstained with 4′,6-diamidino-2-phenylindole (DAPI, Beyotime Biotechnology, China). EdU-positive cells were visualized using a fluorescence microscope (DM3000, Leica, Germany).

Cell apoptosis and cell cycle distribution assay

L-02 cells were seeded into 6-well plates at a density of 1×10^5 cells per well and incubated overnight. Cells were then pretreated with Amifostine (10 μM) or Se-Cy2, Se-Cy3, Se-Cy4, and Se-Cy5 (each at 2.5 μM) for 6 h, followed by exposure to 10 Gy of ^{60}Co γ-ray radiation. For the apoptosis assay, cells were harvested 48 h post-radiation and stained with 5 μL Annexin V-FITC (BD, USA) and 5 μL propidium iodide (PI, BD, USA) at room temperature in the dark for 20 min. The percentage of apoptotic cells was quantified using flow cytometry (Accuric6, BD, USA).

For cell cycle analysis, cells were collected 48 h post-radiation and fixed in 75% cold ethanol at 4 °C for 24 h. After washing twice with PBS, the cells were stained with 300 μL of PI in the dark for 30 min and analyzed by flow cytometry.

Cell cloning assay

L-02 cells were pre-treated with Amifostine (10 μM) or Se-Cy2, Se-Cy3, Se-Cy4, and Se-Cy5 (each at 2.5 μM) for 6 h, followed by exposure to 10 Gy of ^{60}Co γ-ray radiation. After 24 h, 1,000 viable cells from each group were seeded into 6-well plates and allowed to adhere. Cultures were maintained under standard conditions and monitored until visible colonies formed. Colonies were fixed with 4% paraformaldehyde for 15 min, washed twice with PBS, and stained with crystal violet

for 15 min. Excess dye was removed by rinsing under running water, and the plates were air-dried at room temperature.

ATP assay

L-02 cells were seeded into 96-well plates at a density of 3,000 cells per well. Cells were treated according to the experimental design, with appropriate control groups included. The cells were then incubated with 2.5 μM Se-Cys for 6 h, followed by exposure to 10 Gy of ^{60}Co γ-ray irradiation. After 48 h, the plates were removed from the incubator and equilibrated at room temperature for 10 min. ATP levels were measured using an ATP Assay Kit (Beyotime Biotechnology, China) according to the manufacturer's instructions. The assay reagent was added to each well, mixed thoroughly, and the plates were gently shaken at room temperature to ensure complete cell lysis. The samples were then incubated for an additional 10 min to allow the luminescence signal to stabilize.

Calcein-AM/PI assay

L-02 cells were seeded in 6-well plates at 1×10^5 cells per well and incubated overnight. Cells were then treated with Amifostine (10 μM) or Se-Cy2, Se-Cy3, Se-Cy4, and Se-Cy5 (each at 2.5 μM) for 6 h, followed by exposure to 10 Gy of ^{60}Co γ-ray radiation. After radiation, the medium was aspirated, and cells were washed twice with PBS. Then, cellular viability was determined via the Calcein-AM/PI Double Staining Kit (DOJINDO, Japan), followed by image capture using a fluorescence microscopy.

Detection of intracellular Ca^{2+} concentration

L-02 cells were incubated with Amifostine (10 μM) or Se-Cy2, Se-Cy3, Se-Cy4, and Se-Cy5 (each at 2.5 μM) for 6 h. After being exposed to 10 Gy ^{60}Co γ-ray radiation for 48 h, cells were incubated with Fluo-4 AM (3 μg/mL, Thermo Fisher Scientific, USA) for 30 min at 37 °C and washed with PBS. The fluorescence images were captured using a fluorescence microscope to assess intracellular Ca^{2+} accumulation.

RNA sequencing

Total RNA was extracted from L-02 cells using TRIzol reagent (Invitrogen, Thermo Fisher Scientific, USA) according to the manufacturer's instructions. Total RNA sequencing was outsourced to BGI (Shenzhen, China) and performed using the DNBSEQ platform according to the service provider's standard protocol.

Molecular docking

Molecular docking was performed to predict the interactions between key active molecule and target protein. Active molecule Se-Cy4 was sketched in ChemDraw (PerkinElmer, USA), and converted into 3D conformations in ChemBio3D, followed by MM2 energy minimization. The optimized structures were saved in SDF format and converted to PDB format using PyMOL (Schrödinger, USA). Crystal structures of target proteins (Kelch-like ECH-associated protein 1, Keap1, PDB ID: 1ZGK) were retrieved from the Protein Data Bank (PDB, http://www.rcsb.org/). Water molecules were removed, and hydrogen atoms and charges were added using AutoDock Vina. Docking was then carried out and the conformation with the lowest binding energy was selected for further analysis. The binding interactions between ligands and protein targets were visualized using PyMOL.

Biochemical indexes analysis

The biochemical parameters, including malondialdehyde (MDA), superoxide dismutase (SOD), glutathione peroxidase (GPx), and catalase (CAT), were measured using commercial assay kits (Beyotime Biotechnology, China), and absorbance was detected with a microplate reader (Thermo Fisher Scientific, USA).

Western blotting analysis

Proteins from liver tissues, treated cells, or isolated mitochondria (using a mitochondrial isolation kit; Beyotime Biotechnology, China) were lysed on ice with RIPA lysis buffer (Beyotime Biotechnology, China). Protein concentrations were determined using a BCA protein quantification kit (Beyotime Biotechnology, China). Equal amounts of protein (40 μg per sample) were separated by 12% SDS-PAGE and

transferred onto PVDF membranes (BIO-RAD, USA). After blocking with 5% skim milk for 1 h at room temperature, the membranes were incubated overnight at 4 °C with the following primary antibodies: Nrf2 (1:2000, Proteintech, USA), Keap1 (1:2000, Proteintech, USA), HO-1 (1:2000, Proteintech, USA), MFN1 (1:1000, ImmunoWay, USA), MFN2 (1:1000, Cell Signaling Technology, USA), TOM20 (1:2000, Cell Signaling Technology, USA), HSP60 (1:2000, Proteintech, USA), VDAC (1:1000, Aifang, China), SelS (1:2000, Proteintech, USA), GPX1 (1:2000, Proteintech, USA), GPX4 (1:1000, ZEN-Bio, China), GPX2 (1:1000, ZEN-Bio, China), BCL-2 (1:5000, Proteintech, USA), BAX (1:5000, Proteintech, USA), GAPDH (1:5000, Proteintech, USA), and β-actin (1:20000, Proteintech, USA). The membranes were then incubated with horseradish peroxidase (HRP)-conjugated secondary antibodies, including goat anti-rabbit (1:2000, BIO-RAD, USA) and goat anti-mouse (1:5000, BIO-RAD, USA), for 1 h at room temperature. Protein bands were visualized using an enhanced chemiluminescence (ECL) detection kit (BIO-RAD, USA) and semi-quantified with ImageJ software (NIH, USA).

Analysis of Selenium Content

For the cell level, the selenium concentration in the mitochondria of L-02 cells was measured using inductively coupled plasma mass spectrometry (ICP-MS, Thermo Scientific™ iCAP™ TQ). Mitochondria were isolated with a mitochondrial isolation kit and digested with concentrated nitric acid (HNO_3). Selenium content was quantified by comparing with the Se reference material, using a calibration curve prepared from standard selenium solutions.

For the liver tissue, the selenium concentration in the liver of mice was measured using the same ICP-MS method. Mice were euthanized, and their livers were harvested and homogenized in an appropriate buffer. The liver samples were then digested with concentrated nitric acid (HNO_3) using a microwave digestion system (CEM Corporation, USA). After digestion, the samples were cooled and prepared for ICP-MS analysis.

Hemocompatibility assessment

Fresh blood was collected from BALB/c mice and centrifuged at 1000 × g for 10 min to remove the plasma. The red blood cells (RBCs) were washed at least three times with phosphate-buffered saline (PBS) and resuspended in PBS. A 10 μL aliquot of RBC suspension was then mixed with 90 μL of PBS (negative control), deionized water (positive control), or Se-Cy4 solutions at varying concentrations (0 – 20 μM). After incubation for 2 h at 37 °C, the samples were centrifuged at 1000 × g for 10 min to pellet the intact RBCs. Photographs of the samples were taken, and the supernatants were collected for absorbance measurements at 540 nm using a microplate reader.

Biosafety evaluation

To evaluate long-term biosafety, male Balb/c mice were intraperitoneally injected with Se-Cy4 at a dose of 20 mg/kg once every three days for a total of five doses with tissues collected and analyzed after 31 days of observation. At the end of the study, the mice were euthanized, and samples of blood, heart, liver, spleen, lung, and kidney were collected for comprehensive hematological and histopathological analyses.

***In vivo* biodistribution and metabolism**

For biodistribution studies, mice were administered Se-Cy4 via intravenous injection at a dose of 0.5 mg/kg. At various time points, mice were anesthetized for *in vivo* near-infrared (NIR) fluorescence imaging. Following imaging, the mice were humanely euthanized by cervical dislocation, and major organs (including heart, liver, spleen, lungs, kidneys, and intestine) were collected for ex vivo NIR imaging and photographic documentation. Serum samples were also collected for routine blood biochemical analysis.

References

1. Grimme S, Antony J, Ehrlich S, Krieg H. **A consistent and accurate ab initio parametrization of density functional dispersion correction (DFT-D) for the 94 elements H-Pu**. *J Chem Phys*. 2010; **132**: 154104.

2. Momma K, Izumi FJJoAC. **VESTA 3 for three-dimensional visualization of crystal, volumetric and morphology data**. *J Appl Crystallogr*. 2011; **44**: 1272-6.
3. Lu T, Chen F. **Multiwfn: a multifunctional wavefunction analyzer**. *J Comput Chem*. 2012; **33**: 580-92.
4. Riaz R, Parveen S, Rashid M, Shafiq N. **Combined Experimental and Theoretical Insights: Spectroscopic and Molecular Investigation of Polyphenols from Fagonia indica via DFT, UV-vis, and FT-IR Approaches**. *ACS Omega*. 2024; **9**: 730-40.
5. Shalaby MA, Fahim AM, Rizk SA. **Antioxidant activity of novel nitrogen scaffold with docking investigation and correlation of DFT stimulation**. *RSC Adv*. 2023; **13**: 14580-93.

Supplementary Figures

Supplementary Figure 1

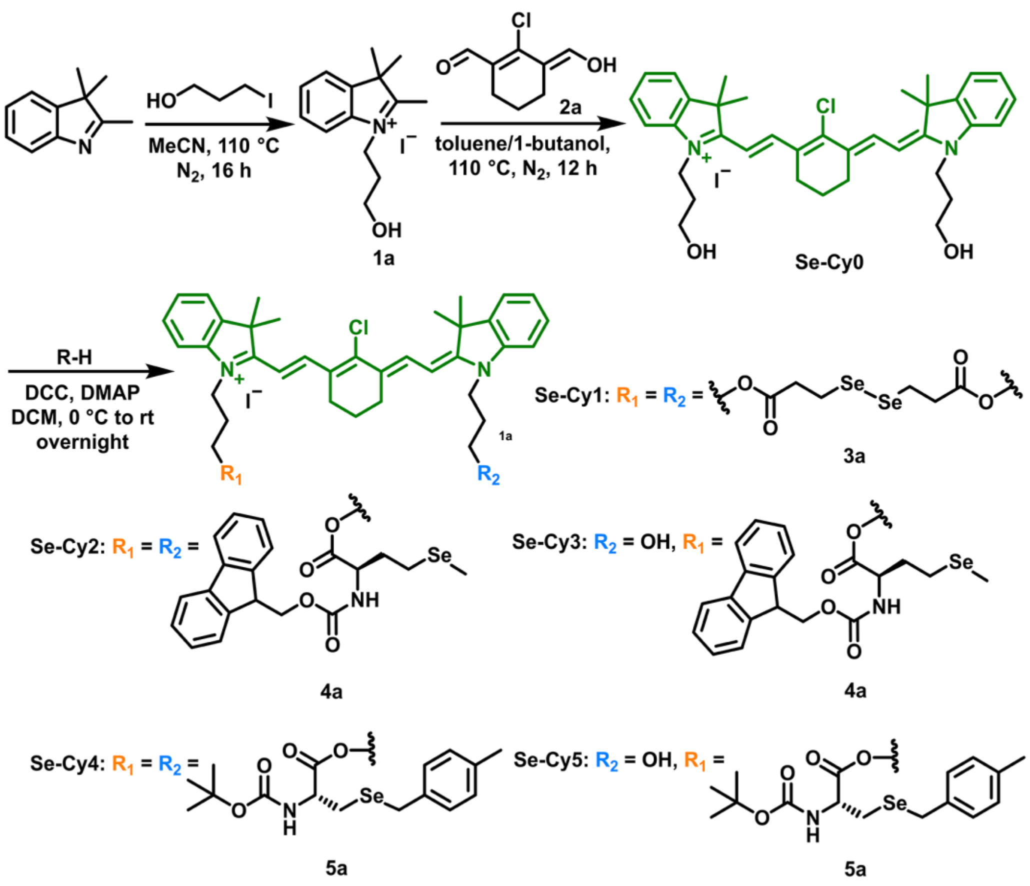

Figure S1. Synthetic routes of different selenium-substituted heptamethine cyanine derivatives (Se-Cys).

Supplementary Figure 2

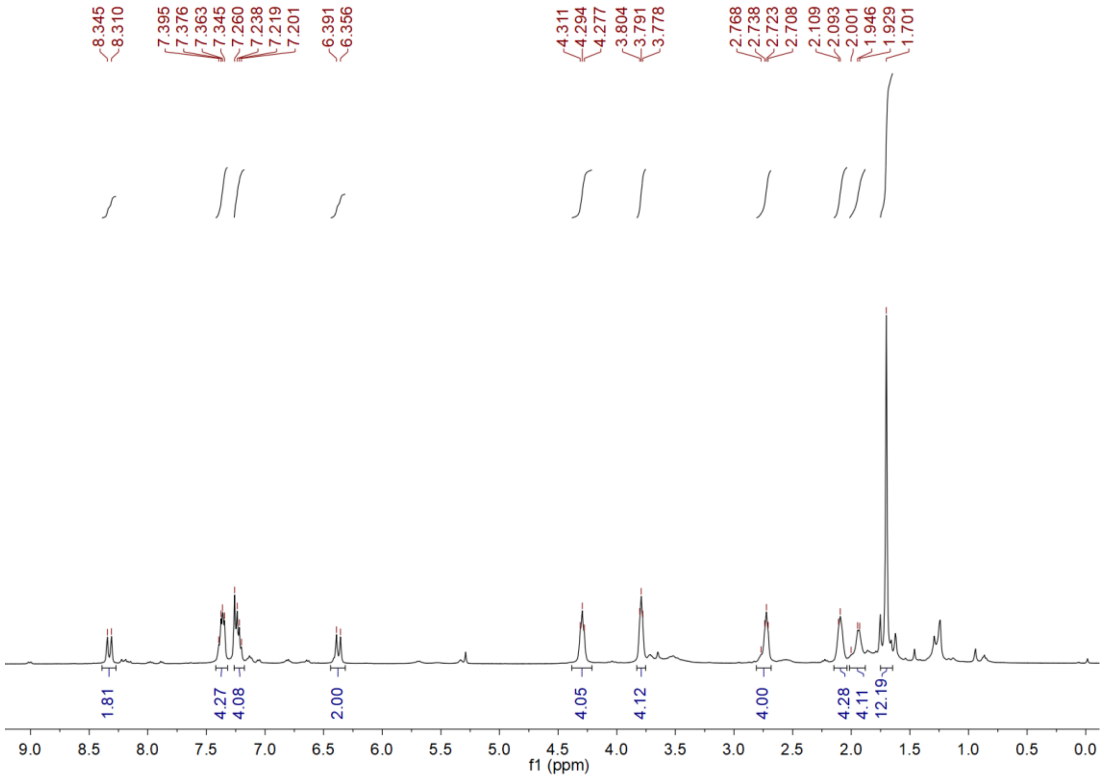

Figure S2. ^{1}H NMR Spectra of Se-Cy0.

Supplementary Figure 3

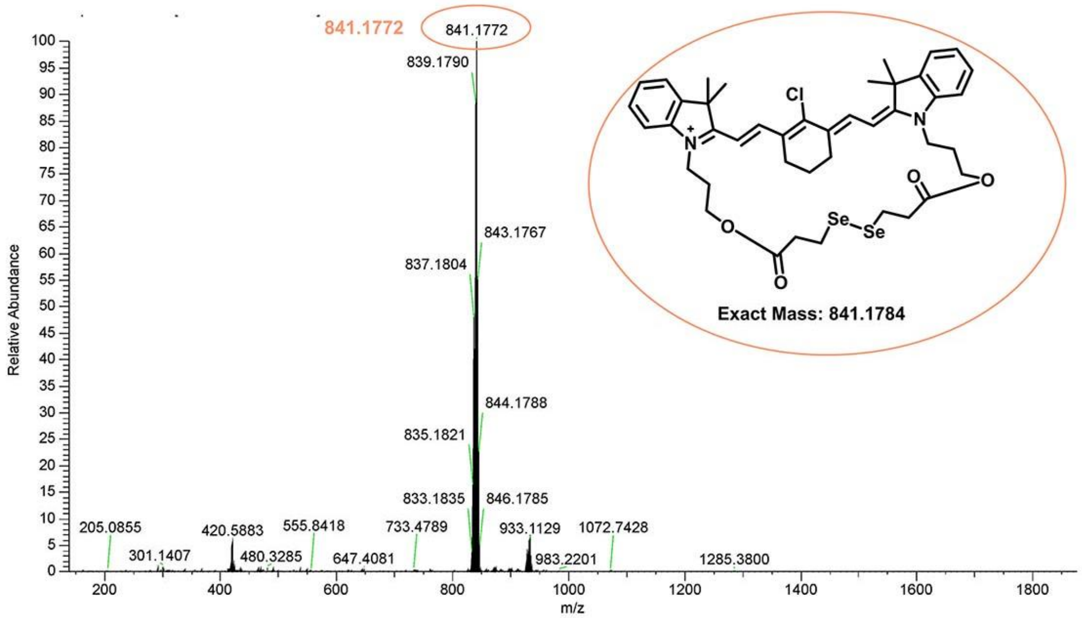

Figure S3. Mass Spectra of Se-Cy1. The molecular weight of Se-Cy1 was determined to be 841.1784, with the primary peak observed at 841.1772 in the mass spectrum.

Supplementary Figure 4

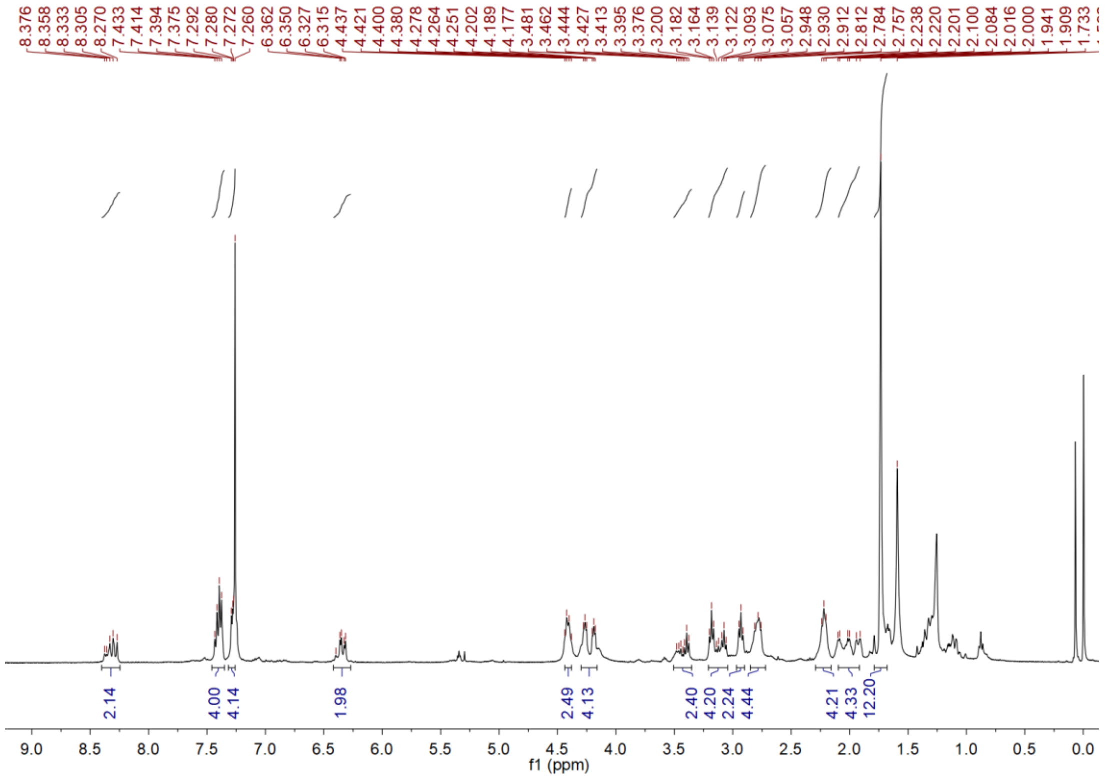

Figure S4. ^{1}H NMR Spectra of Se-Cy1.

Supplementary Figure 5

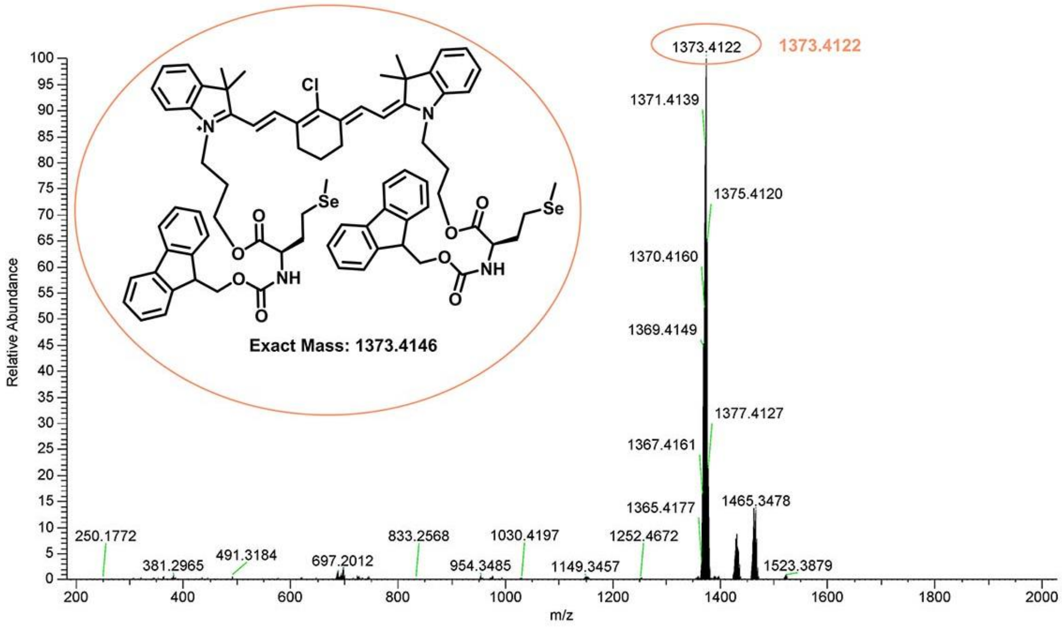

Figure S5. Mass spectra of Se-Cy2. The molecular weight of Se-Cy2 was determined to be 1373.4146, with the primary peak observed at 1373.4122 in the mass spectrum.

Supplementary Figure 6

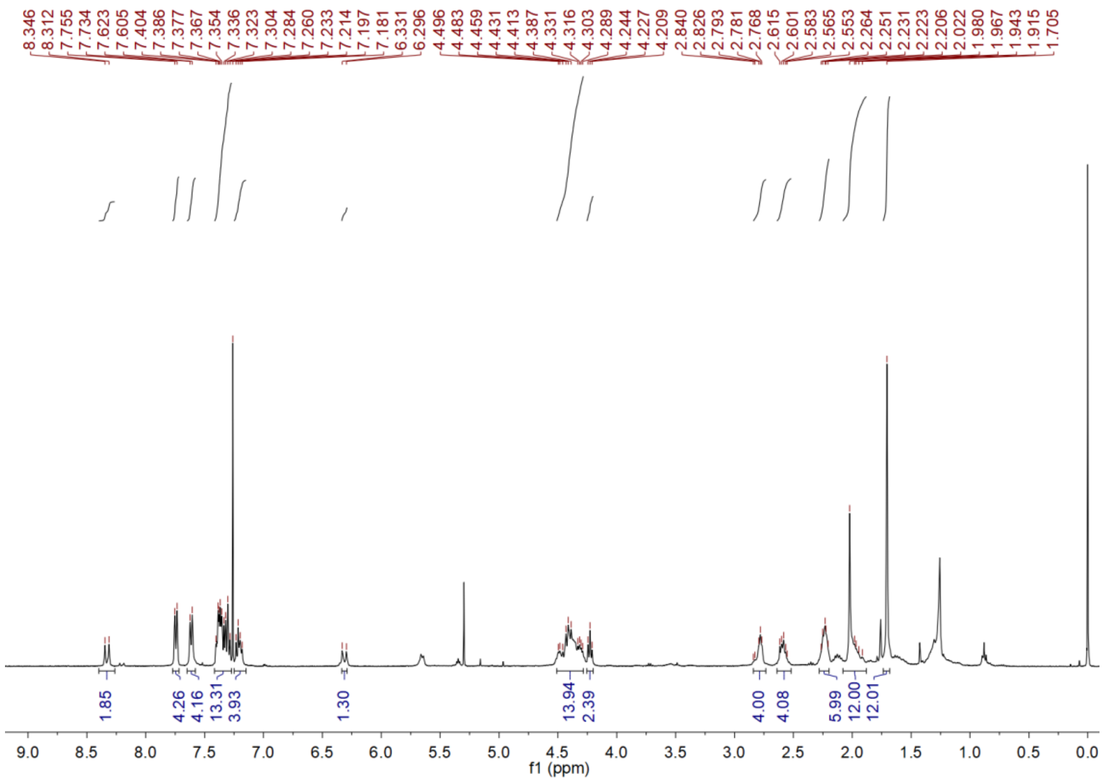

Figure S6. ^{1}H NMR Spectra of Se-Cy2.

Supplementary Figure 7

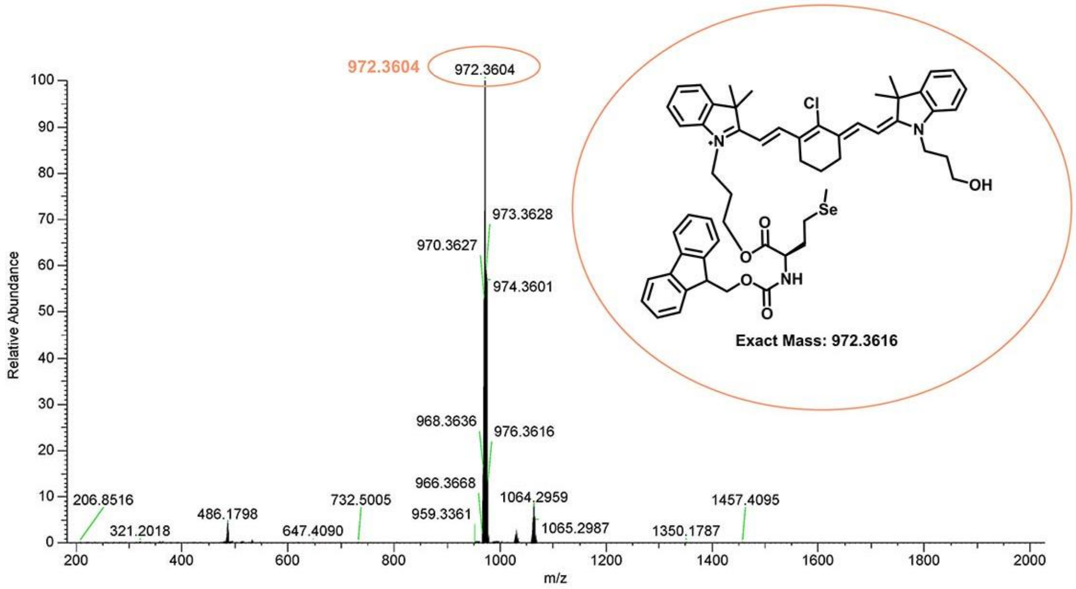

Figure S7. Mass Spectra of Se-Cy3. The molecular weight of Se-Cy3 was determined to be 972.3616, with the primary peak observed at 972.3604 in the mass spectrum.

310 **Supplementary Figure 8**

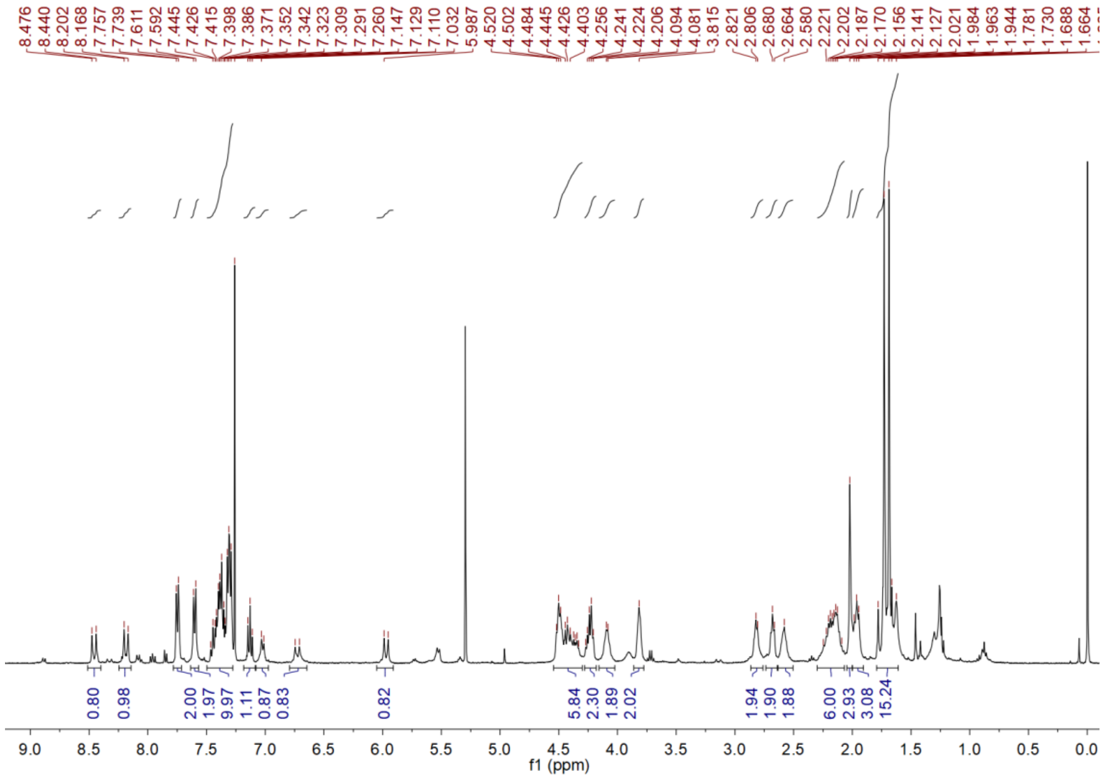

311 **Figure S8. ¹H NMR Spectra of Se-Cy3.**

312

Supplementary Figure 9

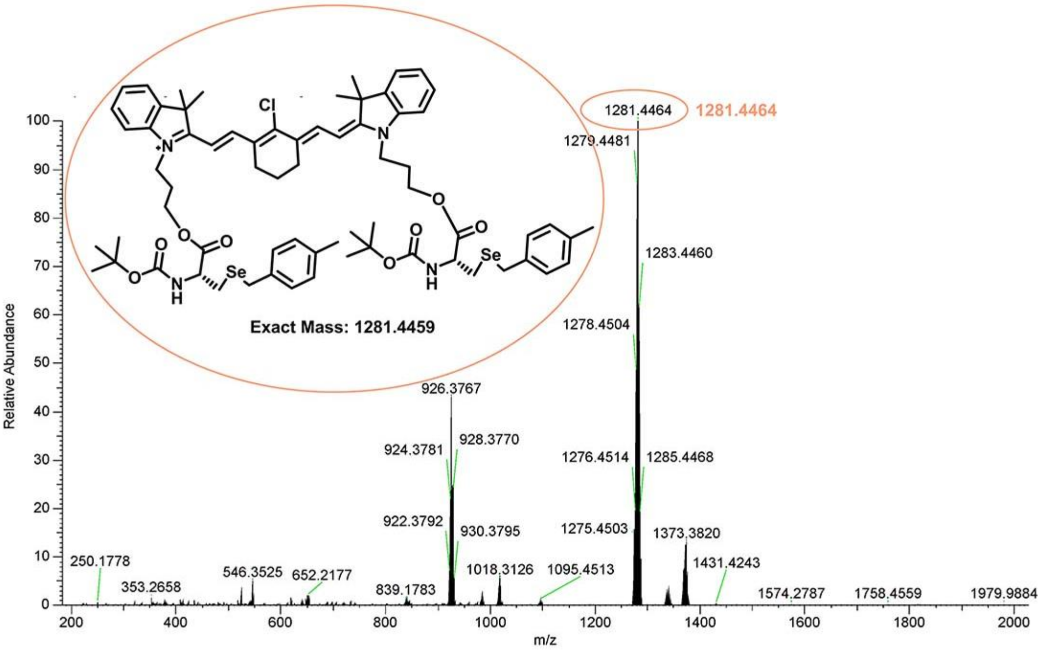

Figure S9. Mass spectra of Se-Cy4. The molecular weight of Se-Cy4 was determined to be 1281.4459, with the primary peak observed at 1281.4464 in the mass spectrum.

Supplementary Figure 10

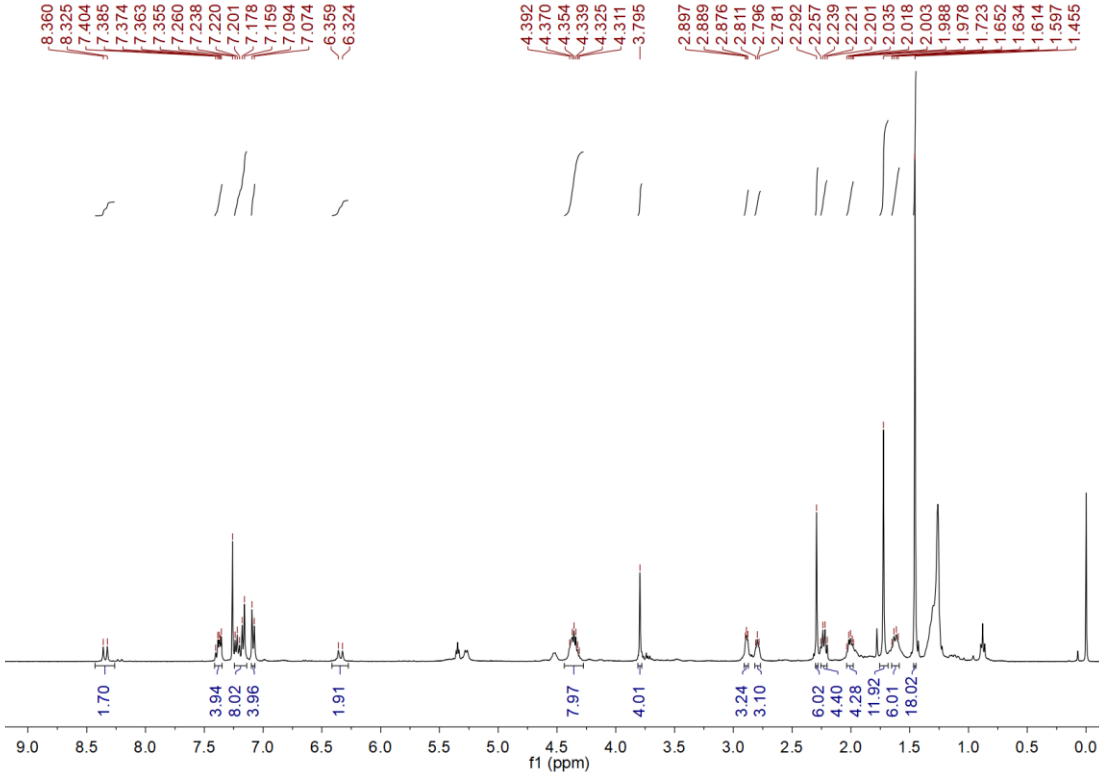

Figure 10. ^{1}H NMR Spectra of Se-Cy4.

Supplementary Figure 11

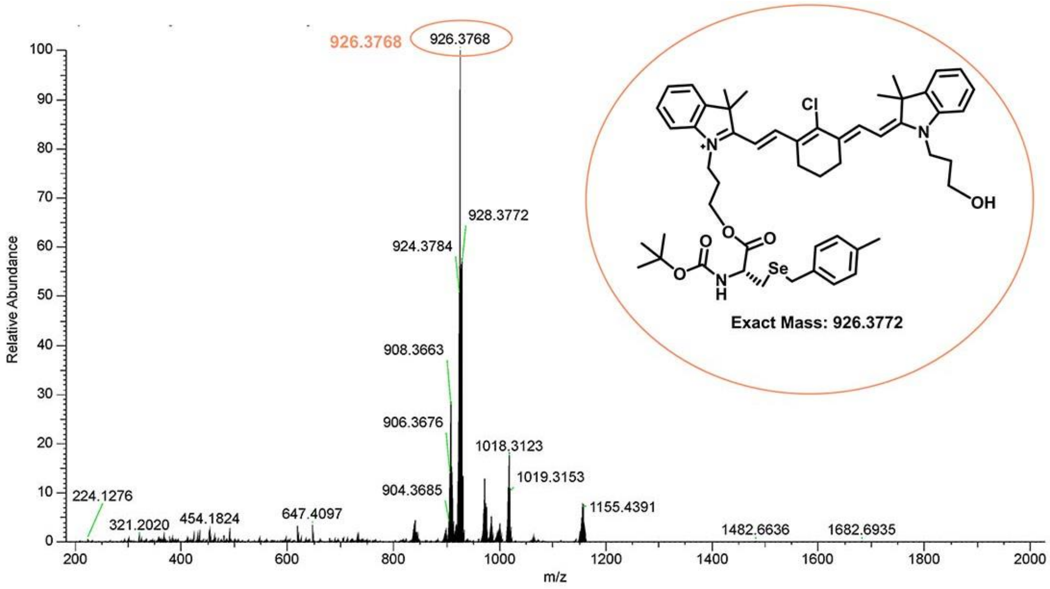

Figure S11. Mass spectra of Se-Cy5. The molecular weight of Se-Cy5 was determined to be 926.3772, with the primary peak observed at 926.3768 in the mass spectrum.

Supplementary Figure 12

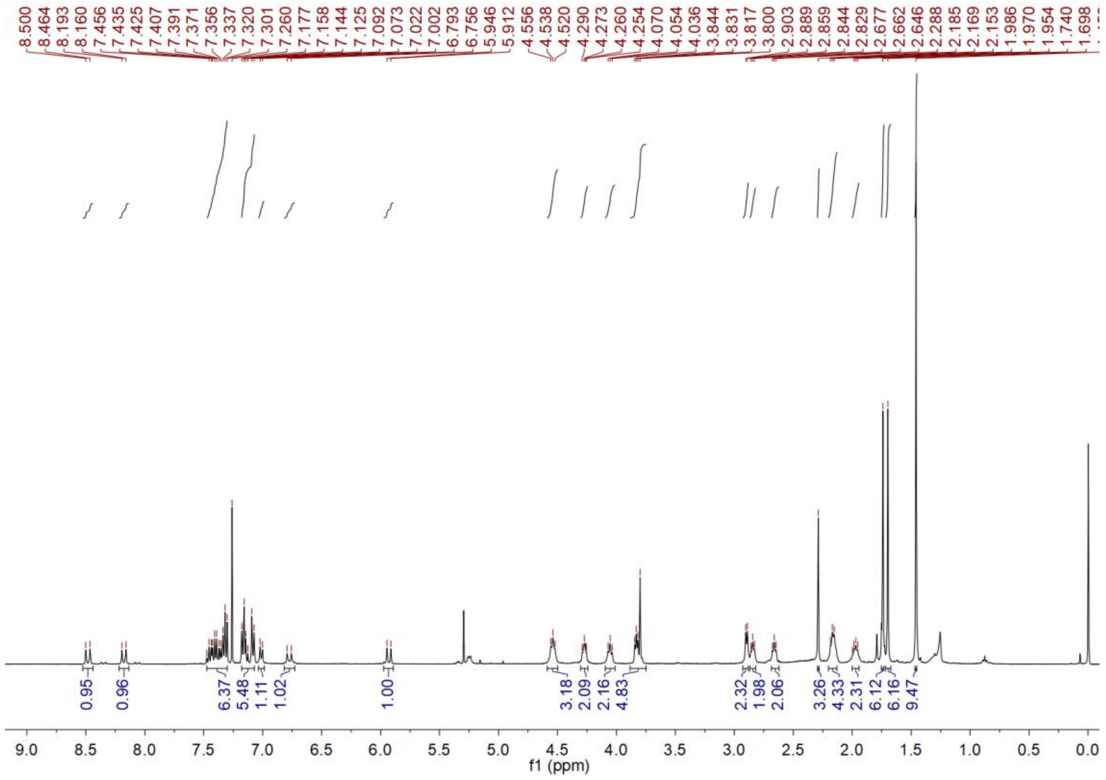

Figure S12. ^{1}H NMR Spectra of Se-Cy5.

Supplementary Figure 13

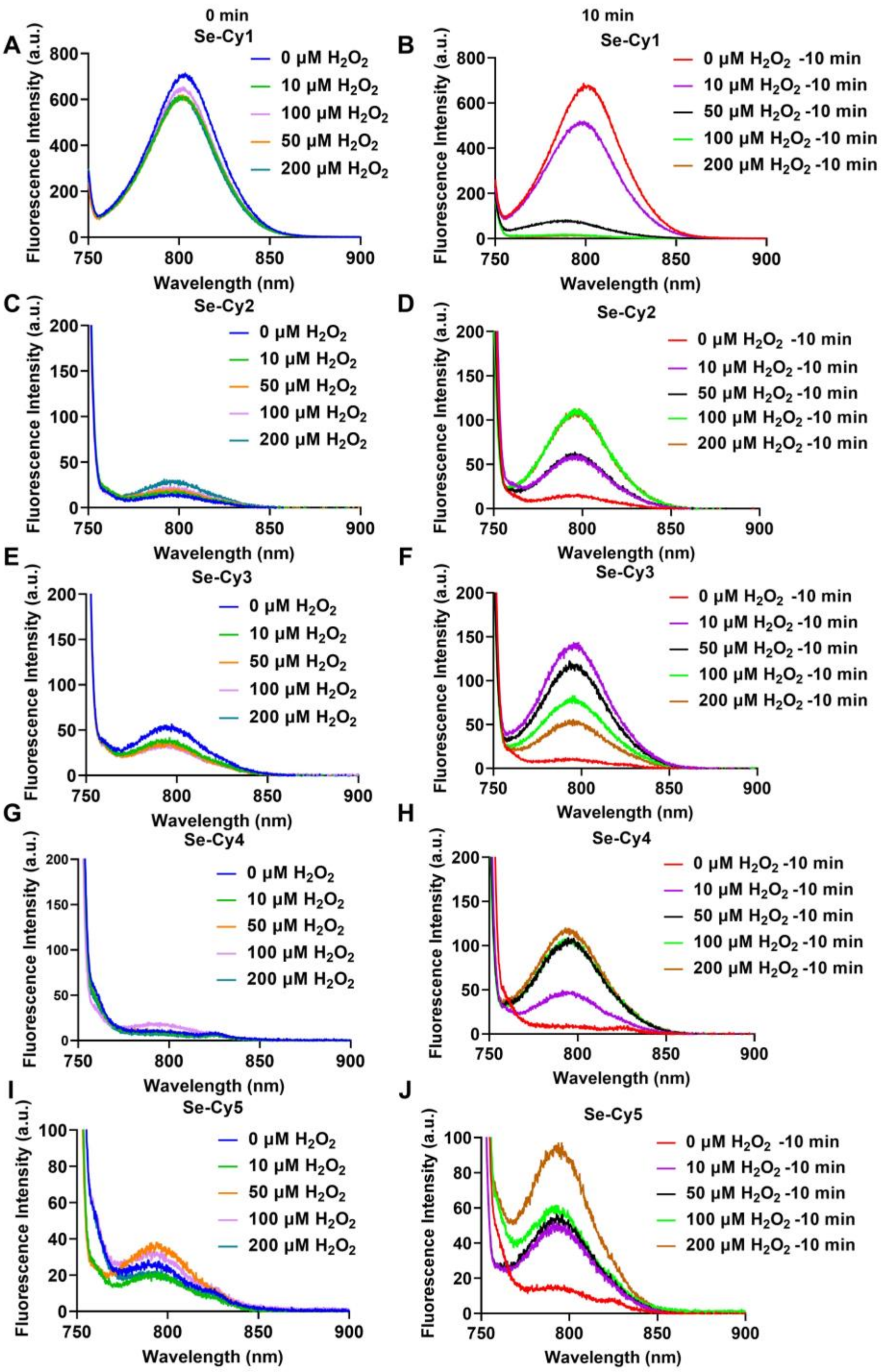

Figure S13. Fluorescence spectra of Se-Cys derivatives in response to H_2O_2 exposure. Fluorescence emission spectra of Se-Cy1 (**A, B**), Se-Cy2 (**C, D**), Se-Cy3 (**E, F**), Se-Cy4 (**G, H**), and Se-Cy5 (**I, J**) in response to increasing concentrations of hydrogen peroxide (H_2O_2) (0, 10, 50, 100, and 200 μM). Panels **A**, **C**, **E**, **G**, **I**: Emission spectra of Se-Cys derivatives were recorded immediately after exposure to varying

concentrations of H_2O_2. Panels **B**, **D**, **F**, **H**, **J**: Emission spectra of Se-Cys derivatives were recorded after a 10-minute exposure to H_2O_2.

Supplementary Figure 14

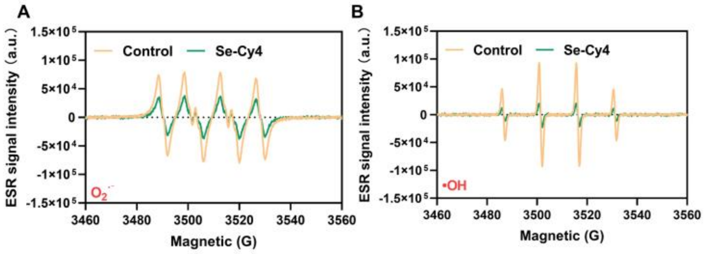

Figure S14. Electron spin resonance (ESR) spectra for evaluating the scavenging activity of Se-Cy4 toward different reactive oxygen species, including (A) superoxide anion ($O_2^{\cdot-}$) and (B) hydroxyl radical (·OH).

Supplementary Figure 15

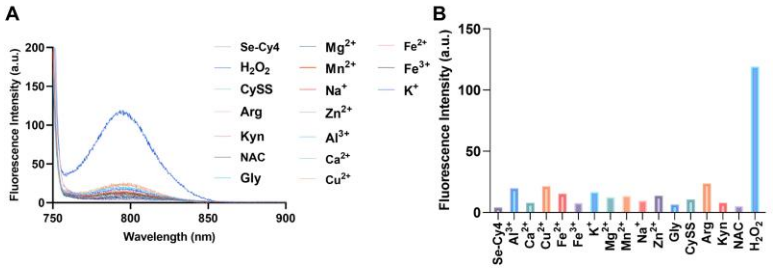

Figure S15. Selective fluorescence response of Se-Cy4 to H_2O_2 against biological interferents. Abbreviations: Cyss: Cystine; Arg: Arginine; Kyn: Kynurenine; NAC: N-acetylcysteine; Gly: Glycine.

Supplementary Figure 16

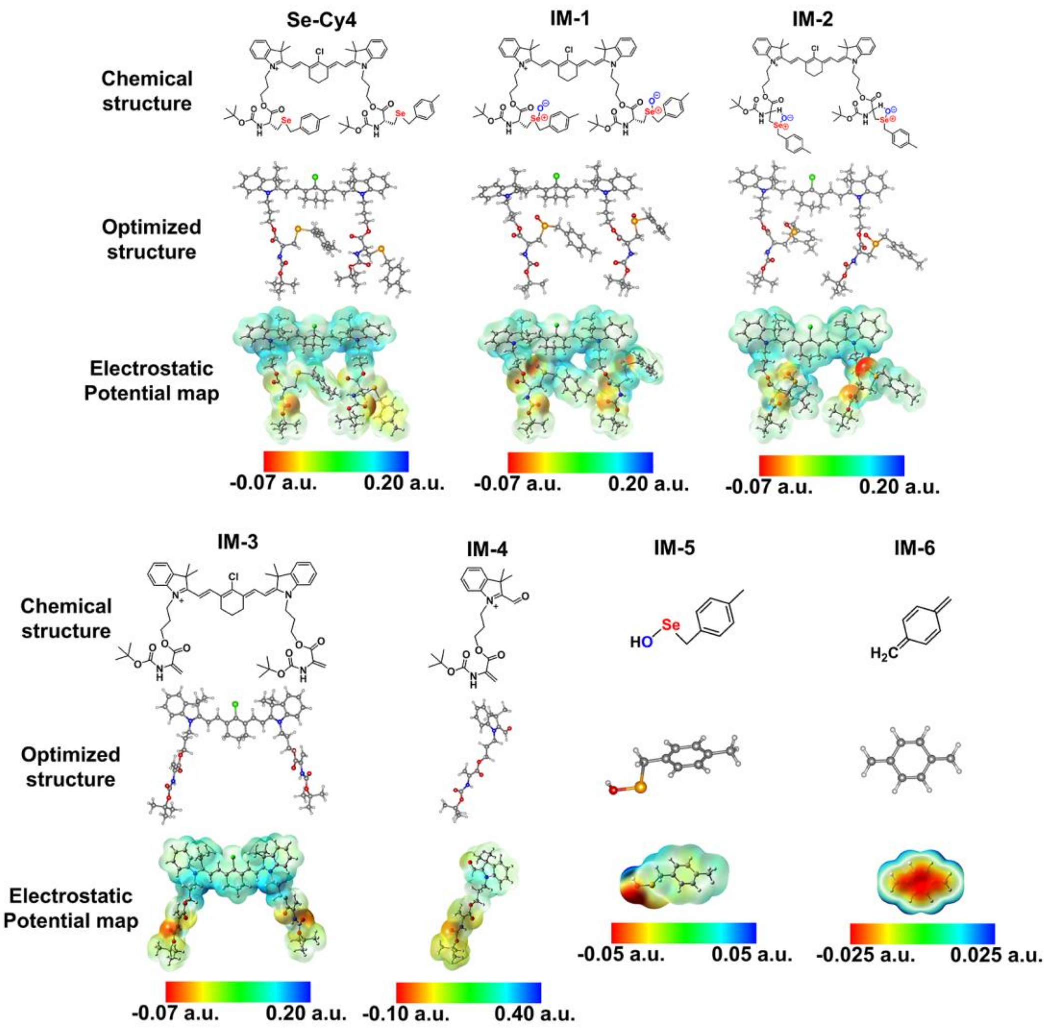

Figure S16. Chemical structures, optimized structures, and electrostatic potential maps of Se-Cy4 and IM-1 – IM-6 by DFT calculation.

Supplementary Figure 17

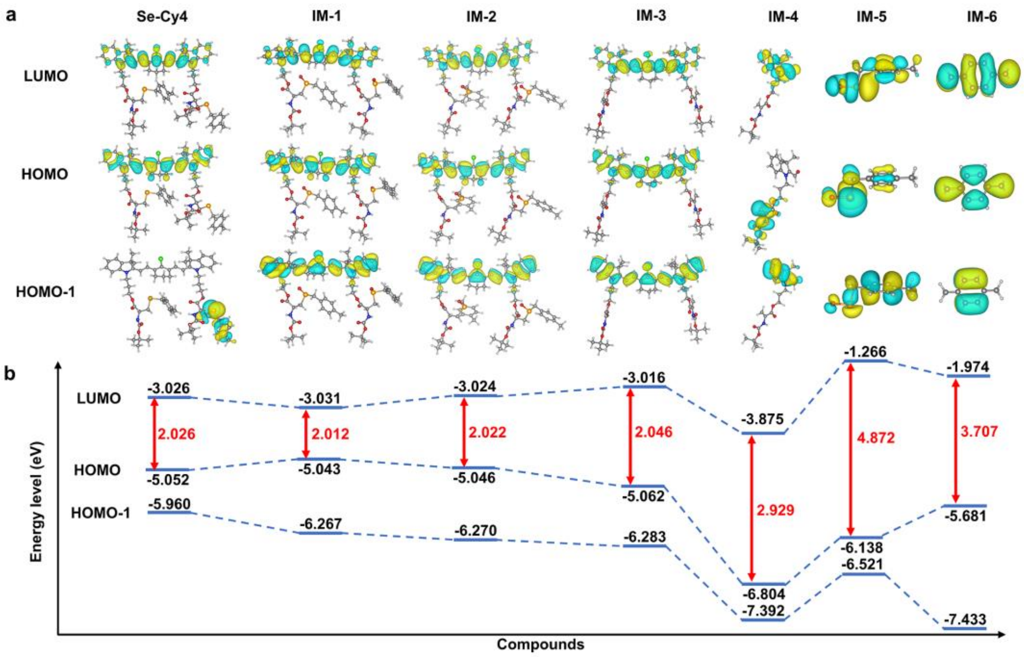

Figure S17. Frontier molecular orbitals (A) and corresponding energy levels (B) of Se-Cy4 and IM-1 – IM-6.

Supplementary Figure 18

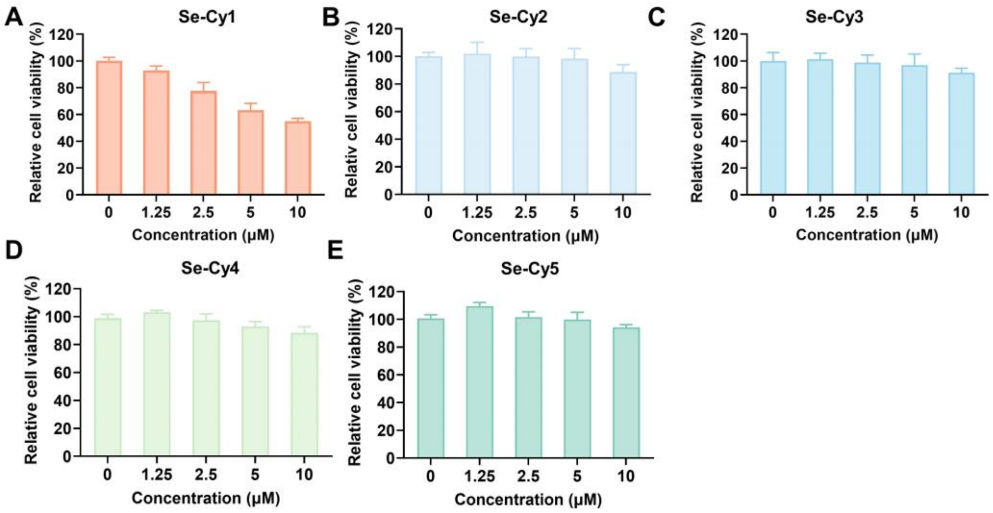

Figure S18. The viability of cells treated with Se-Cys at different concentrations. Cell viability was assessed in L-02 cells treated with various concentrations of Se-Cy1 (**A**), Se-Cy2 (**B**), Se-Cy3 (**C**), Se-Cy4 (**D**), and Se-Cy5 (**E**).

Supplementary Figure 19

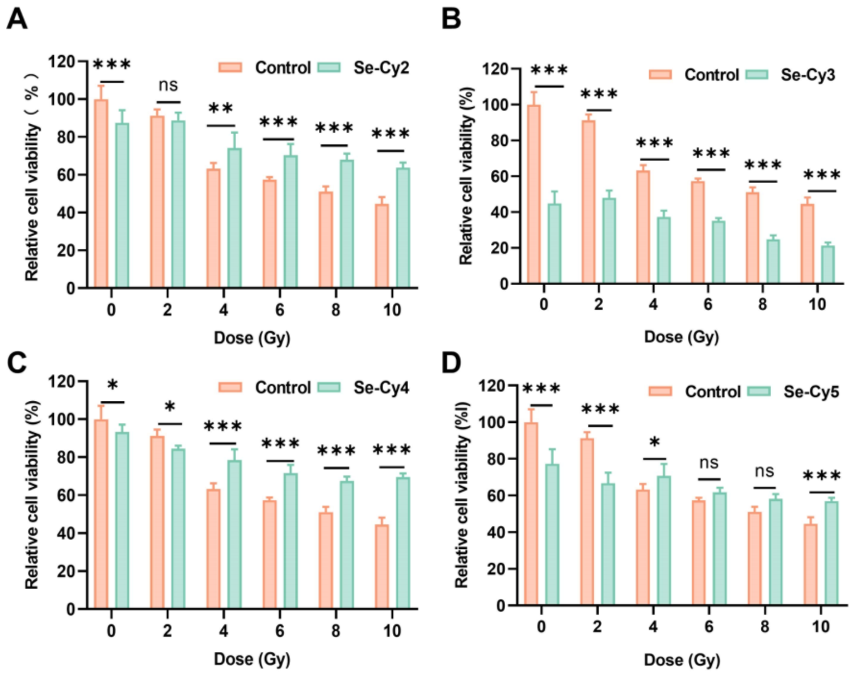

Figure S19. The viability of cells treated with Se-Cys following different doses of radiation, 72 h after radiation exposure. Cell viability was assessed using an CCK-8 assay in L-02 cells 72 h after treated with Se-Cy2, Se-Cy3, Se-Cy4, or Se-Cy5, followed by exposure to varying doses of IR (0, 2, 4, 6, 8, and 10 Gy). Data are presented as mean ± SD (n = 5). **P* < 0.05, ***P* < 0.01, ****P* < 0.001, ns: No significant difference. Statistical significance was determined by one-way ANOVA followed by post hoc tests.

Supplementary Figure 20

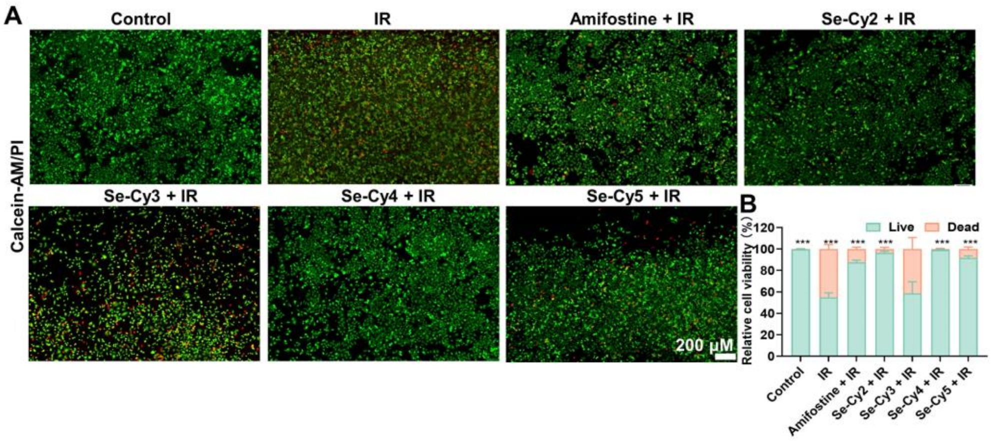

Figure S20. Calcein-AM/PI staining and quantification. (a) Representative fluorescence images of L-02 cells stained with calcein-AM (live cells, green) and propidium iodide (PI; dead cells, red) under the indicated treatments, scale bar = 200 µM. **(b)** Quantification of relative cell viability based on calcein-AM/PI staining in each group, expressed as the percentage of live and dead cells. Data are presented as mean ± SD (n = 3). ***$P < 0.001$. Statistical significance was determined by one-way ANOVA followed by post hoc tests.

Supplementary Figure 21

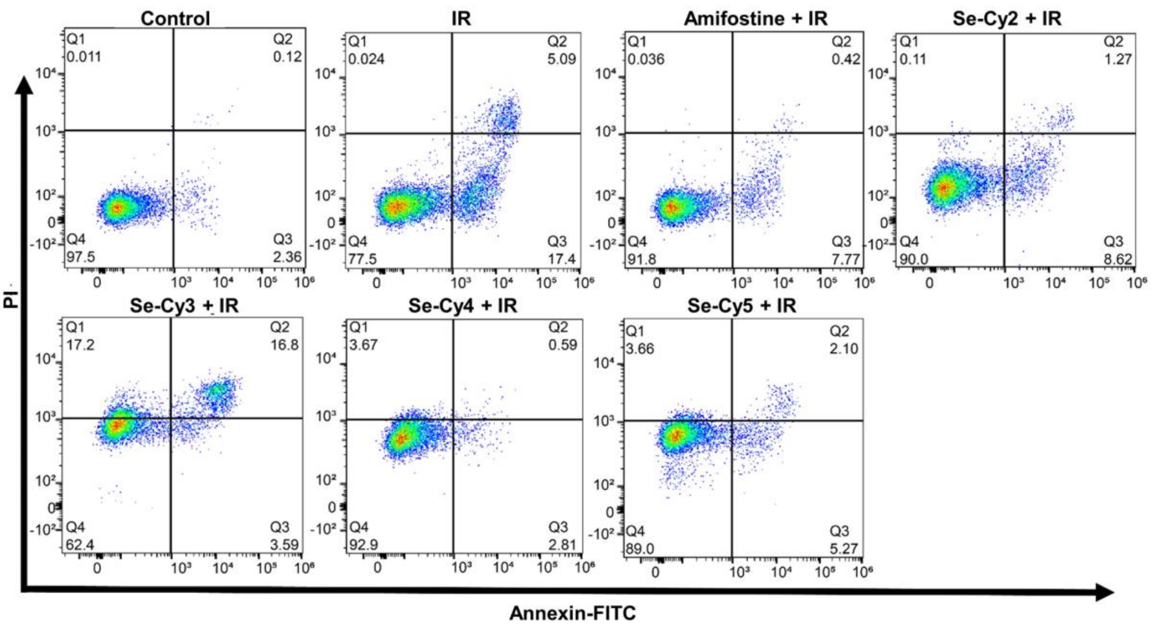

Figure S21. Effect of Se-Cys on apoptosis. Apoptosis analysis revealed that Se-Cy4 pretreatment markedly reduced IR-induced apoptosis.

Supplementary Figure 22

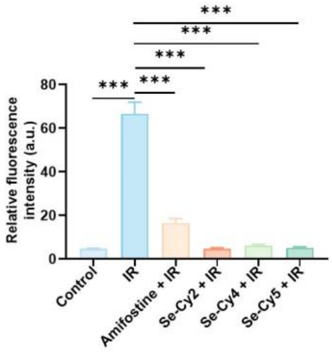

Figure S22. Quantification of relative ROS fluorescence intensity. Data are presented as mean ± SD (n = 3). ***P < 0.001. Statistical significance was determined by one-way ANOVA followed by post hoc tests.

Supplementary Figure 23

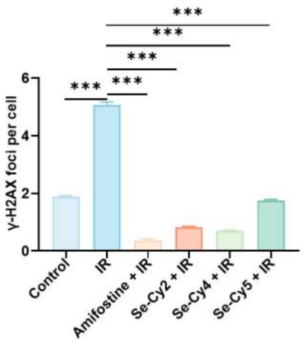

Figure S23. Quantification of γ-H2AX foci. Data are presented as mean ± SD (n = 3). ****P* < 0.001. Statistical significance was determined by one-way ANOVA followed by post hoc tests.

Supplementary Figure 24

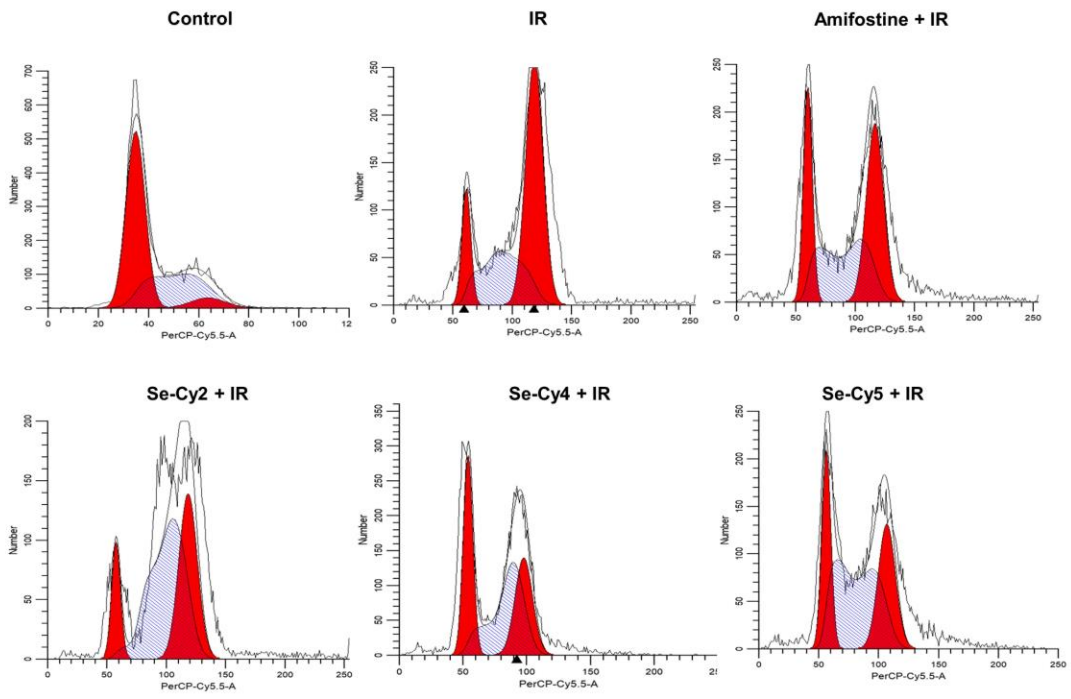

Figure S24. Se-Cy4 reduced IR-induced G2/M arrest in L-02 cells. Se-Cy4 and Se-Cy5 alleviate IR-induced G2/M arrest. IR (10 Gy) increased the G2/M population from 5.98% to 55.54%, which was reduced to 27.16% and 27.18% by Se-Cy4 and Se-Cy5, respectively.

Supplementary Figure 25

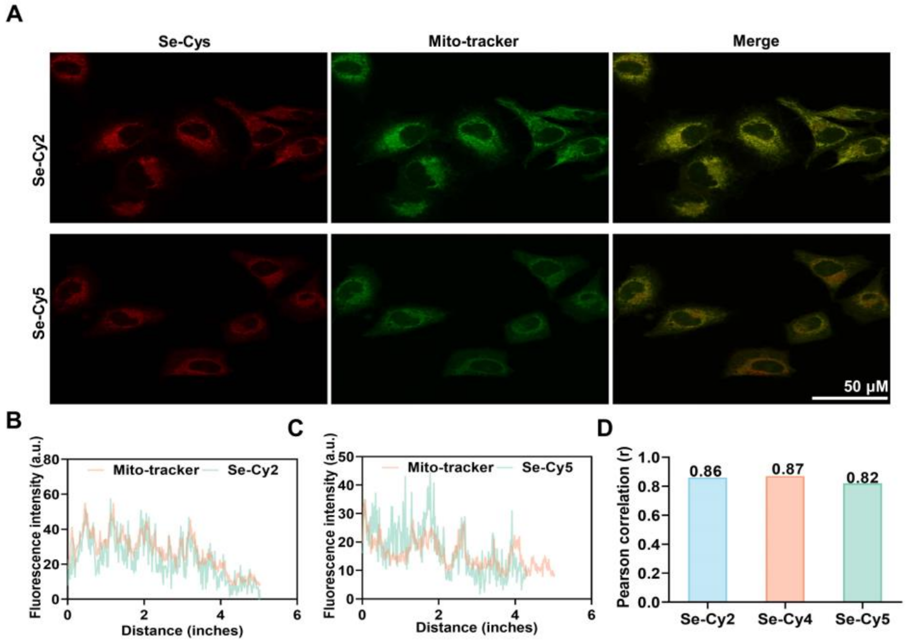

Figure S25. Se-Cys co-localized in mitochondria of L-02 cells. (A) Confocal images showing the localization of Se-Cy2 (red) or Se-Cy5 (red) with mitochondria in L-02 cells, as detected by Mito-tracker (green), scale bar = 50 μM. **(B)** Fluorescence intensity profile of Mito-tracker (green) and Se-Cy2 (red) along a distance in the cells. **(C)** Fluorescence intensity profile of Mito-tracker (green) and Se-Cy5 (red) along a distance in the cells. **(D)** Pearson correlation coefficient analysis of the co-localization between Se-Cy2, Se-Cy4, and Se-Cy5 with Mito-tracker.

Supplementary Figure 26

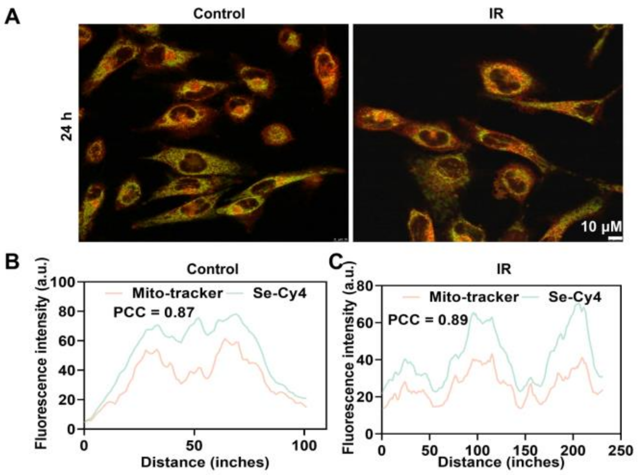

Figure S26. Fluorescence enhancement and mitochondrial retention of Se-Cy4 in liver cells under irradiation. (A) Representative fluorescence images showing colocalization of Se-Cy4 with mitochondria in control and IR groups at 24 h. **(B)** Quantitative analysis of colocalization in the control and IR groups.

Supplementary Figure 27

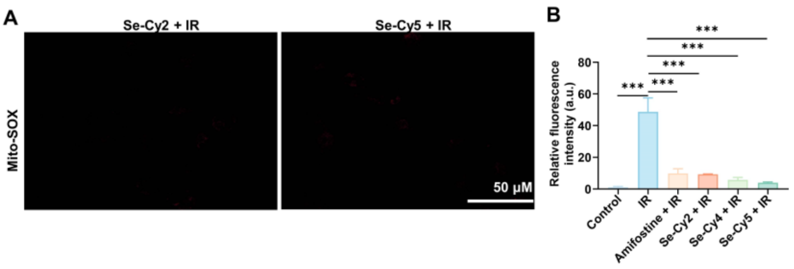

Figure S27. Se-Cys (Se-Cy2 and Se-Cy5) reduced mitochondrial ROS production in irradiated L-02 cells (A) and quantitative analysis of fluorescence intensity (B). Scale bar = 50 μM. Data are presented as mean ± SD (n = 3). ***$P < 0.001$. Statistical significance was determined by one-way ANOVA followed by post hoc tests.

Supplementary Figure 28

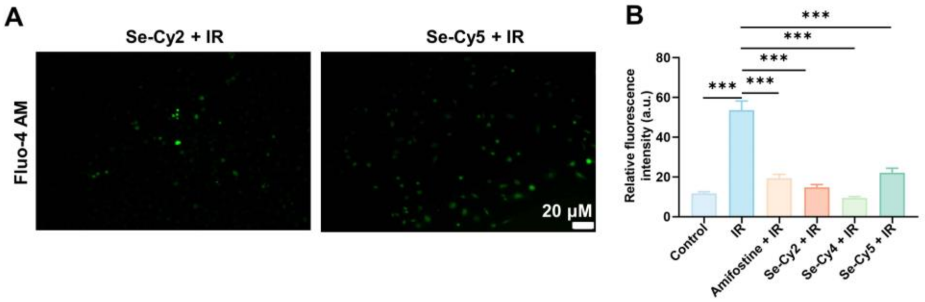

Figure S28. Se-Cys (Se-Cy2 and Se-Cy5) maintained cellular Ca^{2+} levels in irradiated L-02 cells (A) and quantitative analysis of fluorescence intensity (B). Scale bar = 20 μM. Data are presented as mean ± SD (n = 3). ****P* < 0.001. Statistical significance was determined by one-way ANOVA followed by post hoc tests.

Supplementary Figure 29

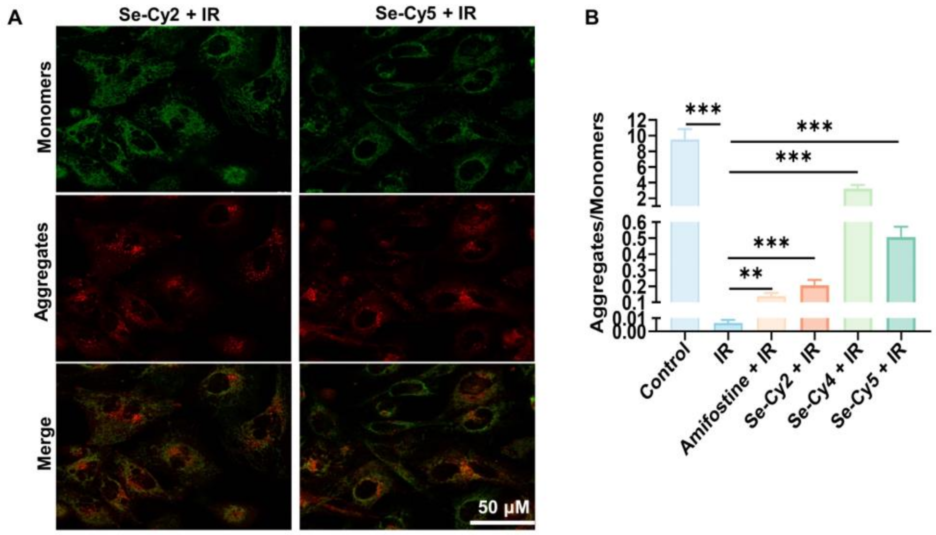

Figure S29. Se-Cys preserved mitochondrial membrane potential in irradiated L-02 cells. (A) Confocal images showing the distribution of monomers (green) and aggregates (red) in L-02 cells after different treatment. Scale bar = 50 μM. **(B)** Quantification of the ratio of aggregates to monomers in cells from different experimental groups. Data are presented as mean ± SD (n = 3). $^{**}P < 0.01$, $^{***}P < 0.001$. Statistical significance was determined by one-way ANOVA followed by post hoc tests.

Supplementary Figure 30

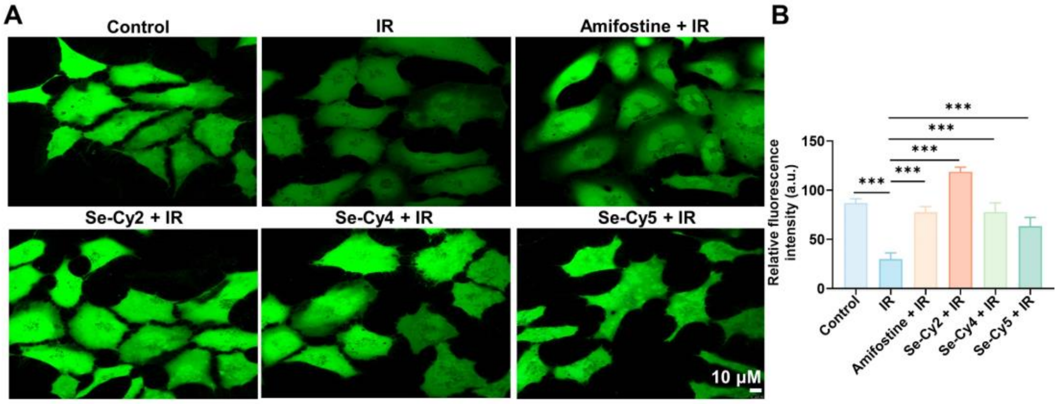

Figure S30. Effects of Se-Cys derivatives on irradiation-induced intracellular acidification. (A) Representative fluorescence images. Scale bar = 10 μM. **(B)** Quantitative analysis of relative fluorescence intensity in cells from different experimental groups. Data are presented as mean ± SD (n = 3). ****P* < 0.001. Statistical significance was determined by one-way ANOVA followed by post hoc tests.

Supplementary Figure 31

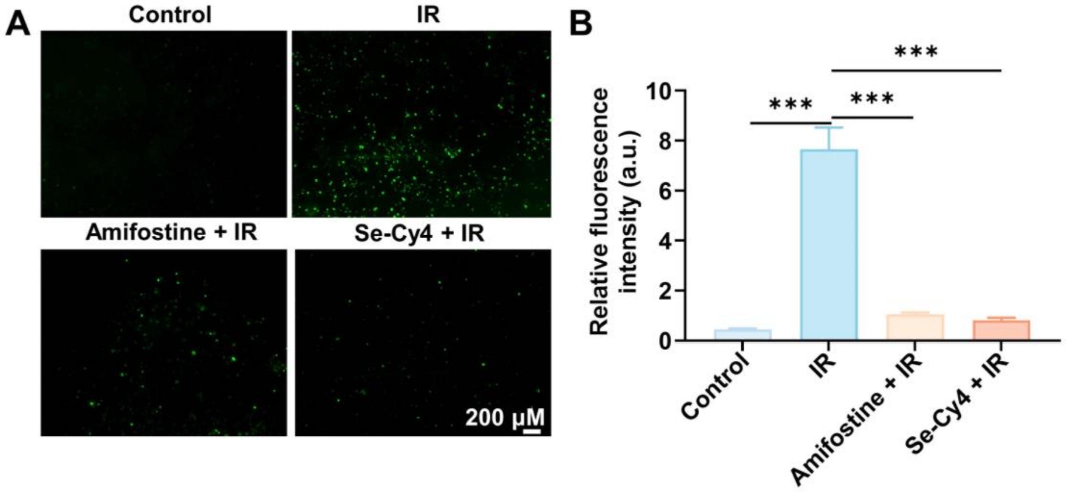

Figure S31. Assessment of lipid peroxidation by Liperfluo staining. (A) Representative fluorescence images of Liperfluo from different groups. Scale bar = 200 μM. **(B)** Quantitative analysis of relative fluorescence intensity in cells from different experimental groups. Data are presented as mean ± SD (n = 3). ***$P < 0.001$. Statistical significance was determined by one-way ANOVA followed by post hoc tests.

Supplementary Figure 32

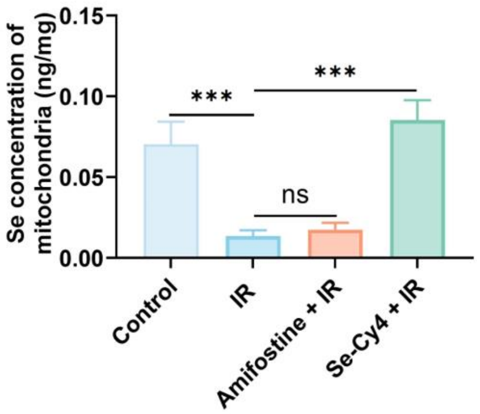

Figure S32. Se-Cy4 increased mitochondrial selenium levels in irradiated L-02 cells. The selenium concentration in mitochondria of L-02 cells with or without Se-Cy4 treatment followed by IR exposure, was determined using ICP-MS. Data are presented as the mean ± SD (n = 3). $^{***}P < 0.001$, ns: No significant difference. Statistical significance was determined by one-way ANOVA followed by post hoc tests.

Supplementary Figure 33

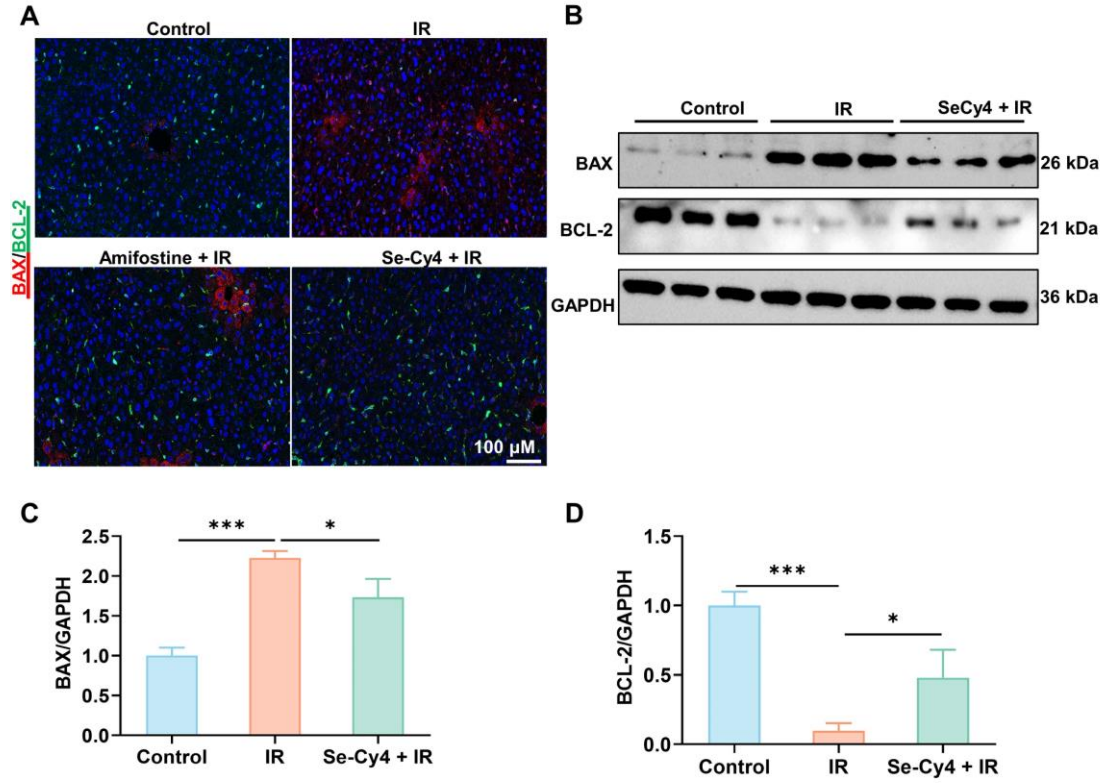

Figure 33. Se-Cy4 inhibited cell apoptosis in irradiated liver. (A) Immunofluorescence staining of BAX (green) and BCL-2 (red) proteins in liver tissues from different experimental groups. DAPI staining (blue) was used to visualize the nuclei. Scale bar = 100 μm. **(B)** Western blot analysis of BAX and BCL-2 in liver tissues from different experimental groups. GAPDH was used as a loading control. **(C-D)** Quantitative densitometric analysis of BAX **(C)** and BCL-2 **(D)** protein levels in liver tissues. Data are presented as the mean ± SD. (n = 3). **P* < 0.05, ****P* < 0.001. Statistical significance was determined by one-way ANOVA followed by post hoc tests.

Supplementary Figure 34

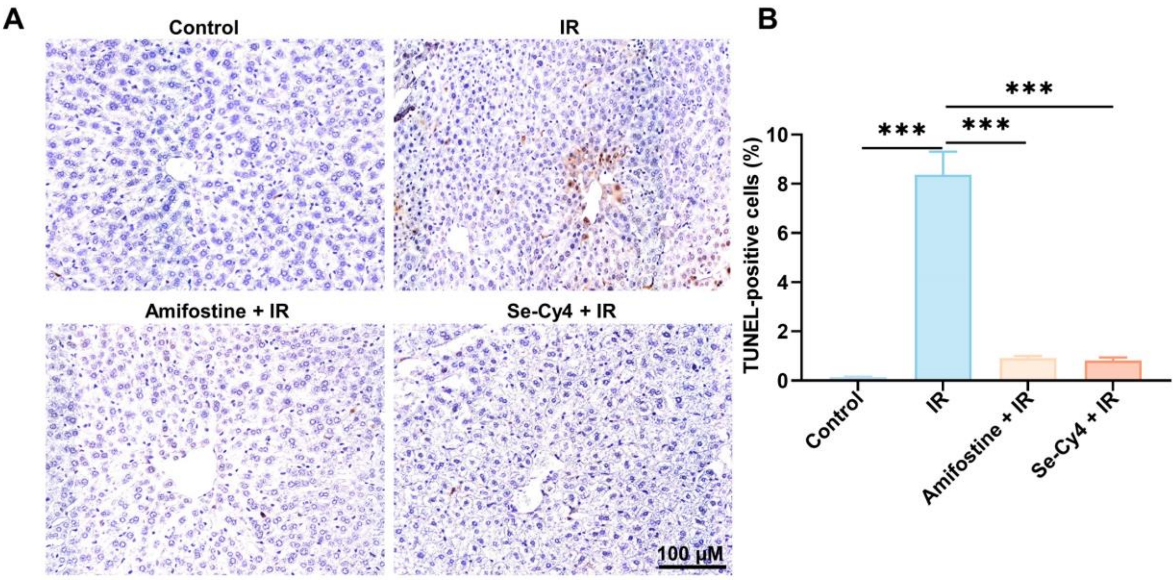

Figure 34. TUNEL staining of different groups. (A) Staining images of TUNEL. Scale bar = 100 μm. **(B)** Quantitative analysis from different experimental groups. Data are presented as the mean ± SD. (n = 3). ****P* < 0.001. Statistical significance was determined by one-way ANOVA followed by post hoc tests.

Supplementary Figure 35

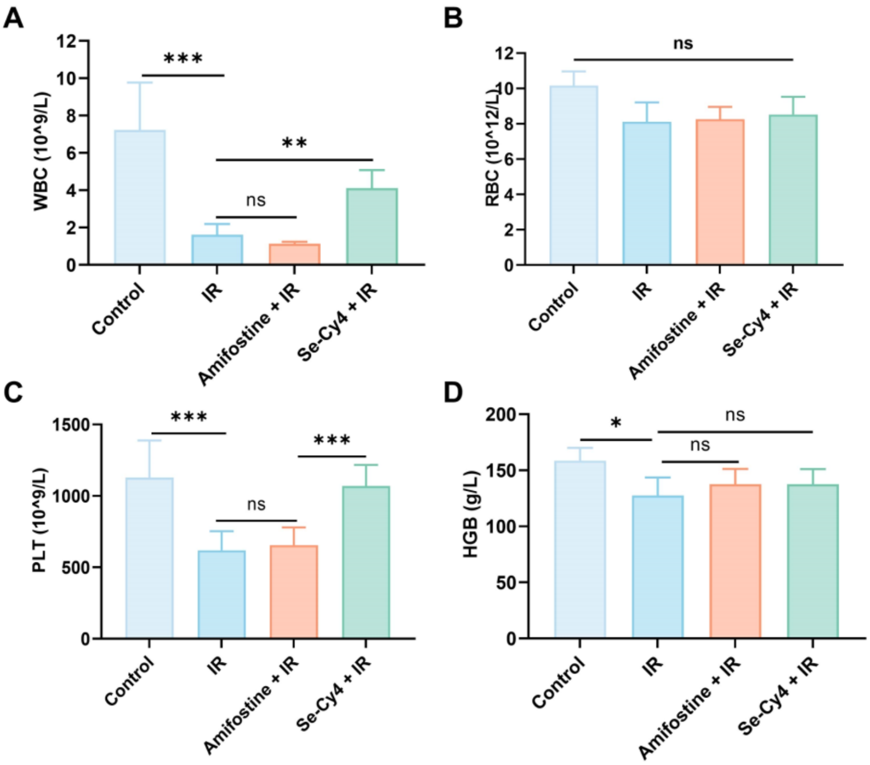

Figure S35. Se-Cy4 mitigated IR-induced hematopoietic injury. (A) White blood cell (WBC) counts, **(B)** red blood cell (RBC) counts, **(C)** platelet (PLT) counts, and **(D)** hemoglobin (HGB) levels in mice after different treatments. Data are presented as the mean ± SD (n = 5). **P* < 0.05, ***P* < 0.01, ****P* < 0.001, ns: No significant difference. Statistical significance was determined by one-way ANOVA followed by post hoc tests.

Supplementary Figure 36

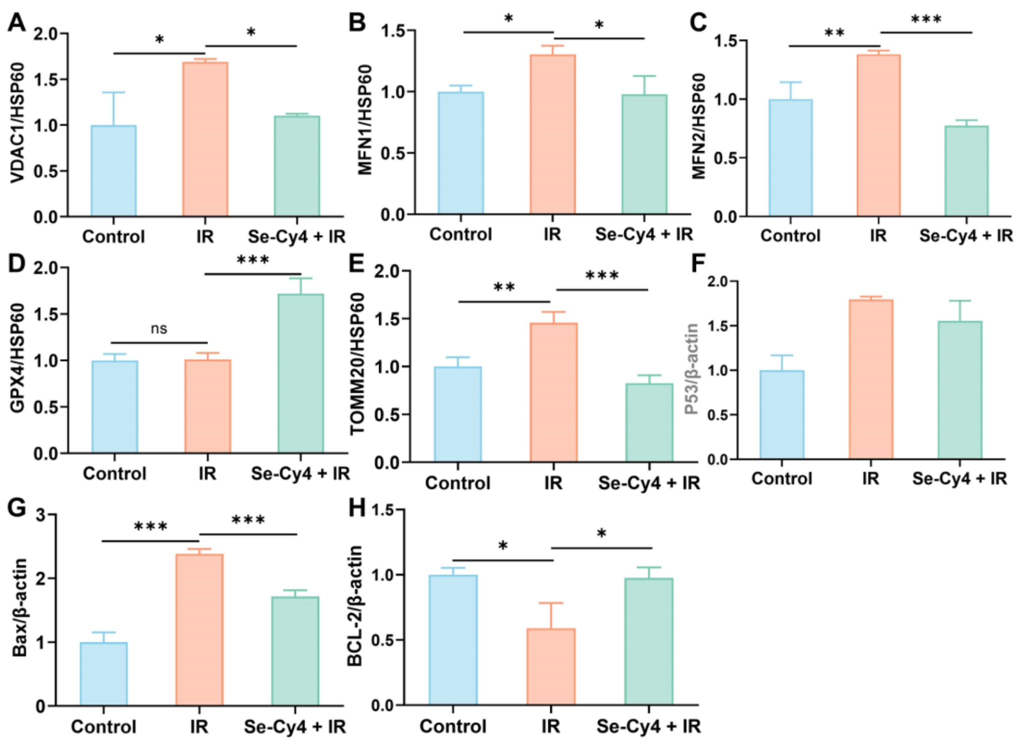

Figure 36. Quantitative analysis of WB. Data are presented as the mean ± SD. (n = 3). *P < 0.05, **P < 0.01, ***P < 0.001, ns: No significant difference. Statistical significance was determined by one-way ANOVA followed by post hoc tests.

Supplementary Figure 37

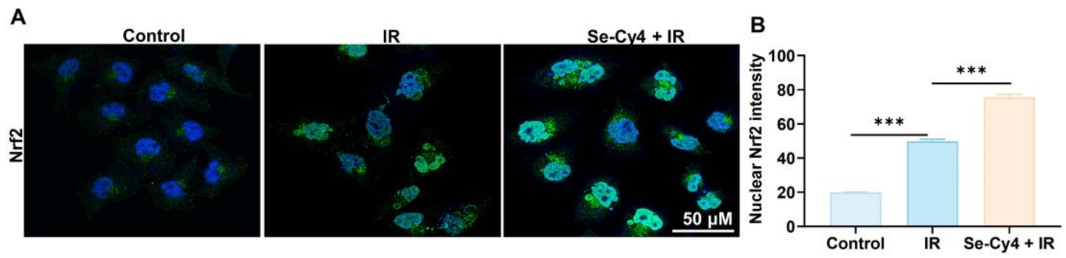

Figure 37. Se-Cy4 activates Keap1–Nrf2 signaling to mitigate irradiation-induced oxidative injury in L-02 cells. (A) Immunofluorescence staining of Nrf2 (green) and in cells from different experimental groups. DAPI staining (blue) was used to visualize the nuclei. Scale bar = 50 μm. **(B)** Quantitative analysis from different experimental groups. Data are presented as the mean ± SD. (n = 3). ****P* < 0.001. Statistical significance was determined by one-way ANOVA followed by post hoc tests.

Supplementary Figure 38

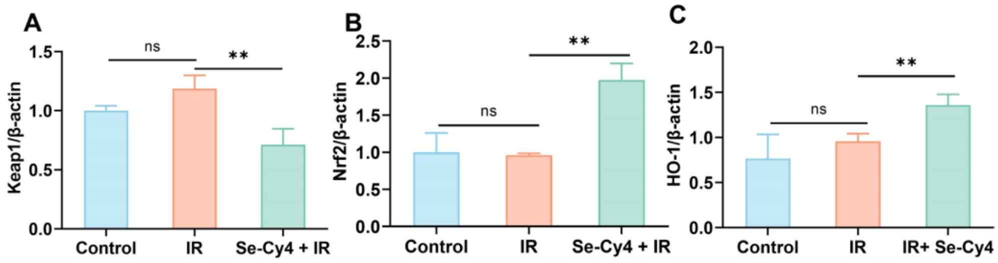

Figure 38. Quantitative densitometric analysis of Keap1 (A), Nrf2 (B), and HO-1 (C). Data are presented as the mean ± SD (n = 3). **P < 0.01, ns: No significant difference. Statistical significance was determined by one-way ANOVA followed by post hoc tests.

Supplementary Figure 39

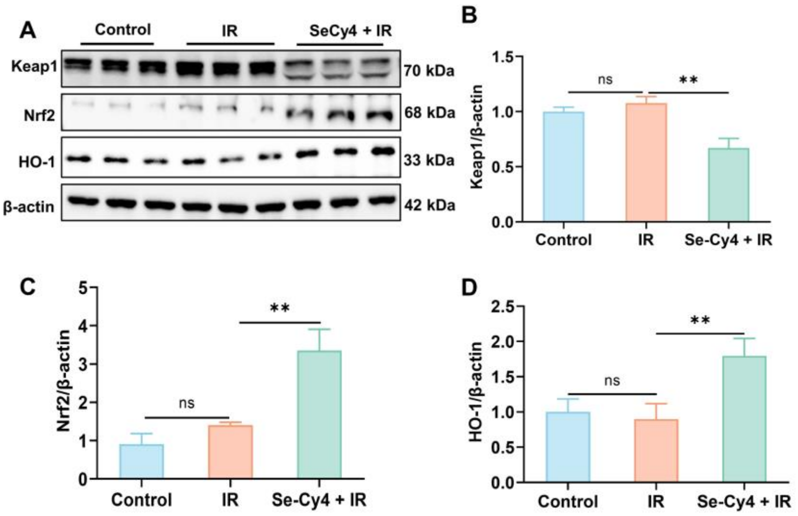

Figure 39. Se-Cy4 activated the Keap1-Nrf2 pathway in irradiated liver. (A) Western blot analysis showing the expression levels of Keap1, Nrf2, and HO-1 in liver tissues from mice exposed to IR with or without Se-Cy4 treatment. β-actin was used as a loading control. **(B-D)** Quantitative densitometric analysis of Keap1 **(B)**, Nrf2 **(C)**, and HO-1 **(D)** protein levels in liver tissues. Data are presented as the mean ± SD (n = 3). $^{**}P < 0.01$, ns: No significant difference. Statistical significance was determined by one-way ANOVA followed by post hoc tests.

Supplementary Figure 40

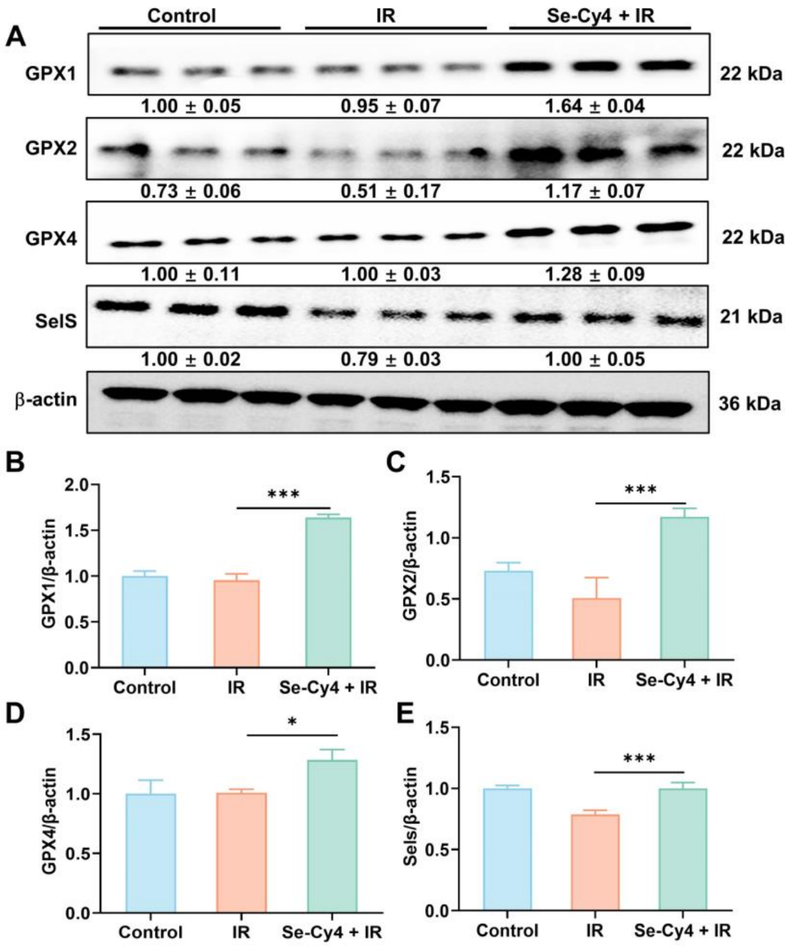

Figure 40. Se-Cy4 restored antioxidant defense in irradiated L-02 by upregulating GPX family proteins. Western blot analysis of antioxidant-related proteins (GPX1, GPX2, GPX4, and SELS) across treatment groups. Data are presented as the mean ± SD (n = 3). **P* < 0.05, ****P* < 0.001, ns: No significant difference. Statistical significance was determined by one-way ANOVA followed by post hoc tests.

Supplementary Figure 41

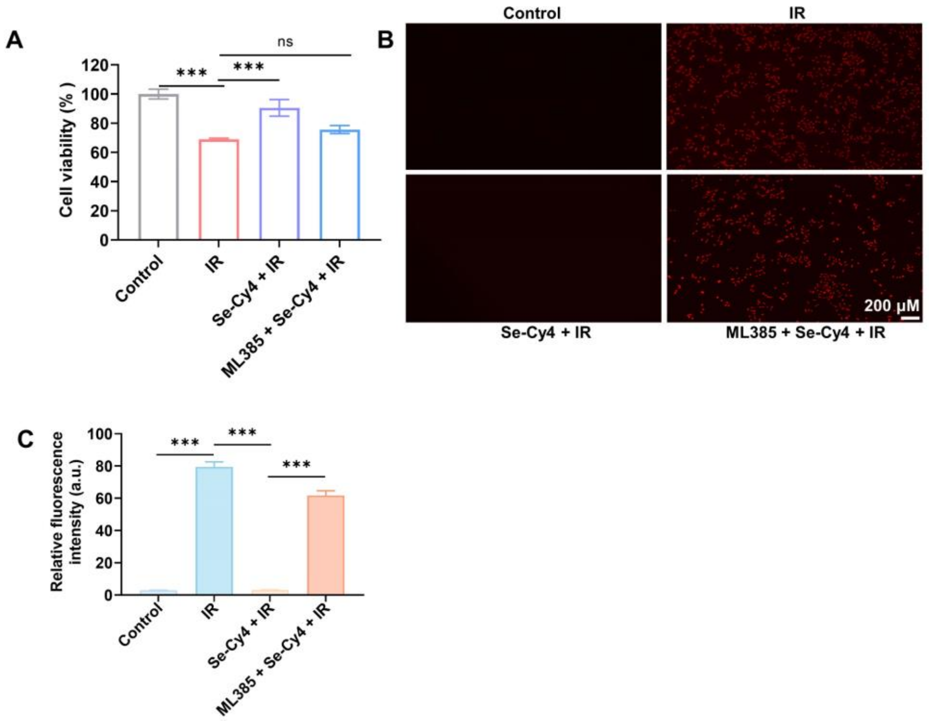

Figure 41. Nrf2 inhibitor ML385 attenuated the radioprotective effect of Se-Cy4. **(A)** Cell viability was assessed in cells exposed to IR and treated with Se-Cy4 alone or in combination with the Nrf2 inhibitor ML385. **(B)** PI dead staining of cells across treatment groups. Scale bars, 200 μm. **(C)** Quantitative analysis from different experimental groups. Data are presented as the mean ±SD (n = 3). ****P* < 0.001, ns: No significant difference. Statistical significance was determined by one-way ANOVA followed by post hoc tests.

Supplementary Figure 42

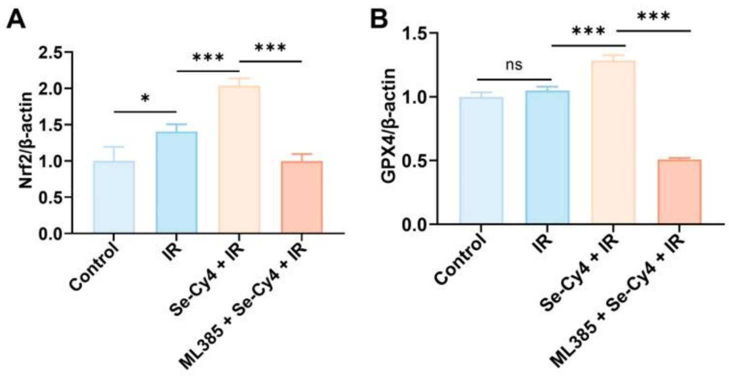

Figure 42. Quantitative analysis of WB. Data are presented as the mean ± SD. (n = 3). **P* < 0.05, ****P* < 0.001, ns: No significant difference. Statistical significance was determined by one-way ANOVA followed by post hoc tests.

Supplementary Figure 43

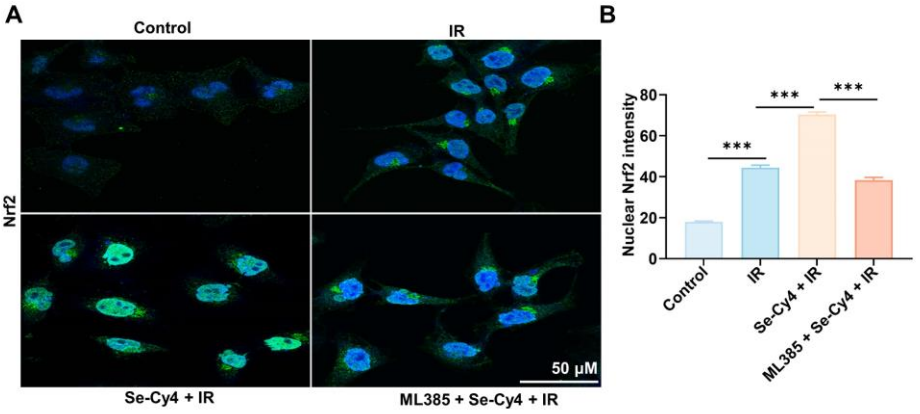

Figure 43. ML385 inhibited Se-Cy4-induced Nrf2 activation under IR conditions. Immunofluorescence staining of Nrf2 in control, IR, Se-Cy4 + IR, and ML385 + Se-Cy4 + IR groups. The inhibitor experiments demonstrated that Nrf2 activation is required for the radioprotective effect of Se-Cy4. Nuclei were counterstained with DAPI. Scale bars, 50 μm.

Supplementary Figure 44

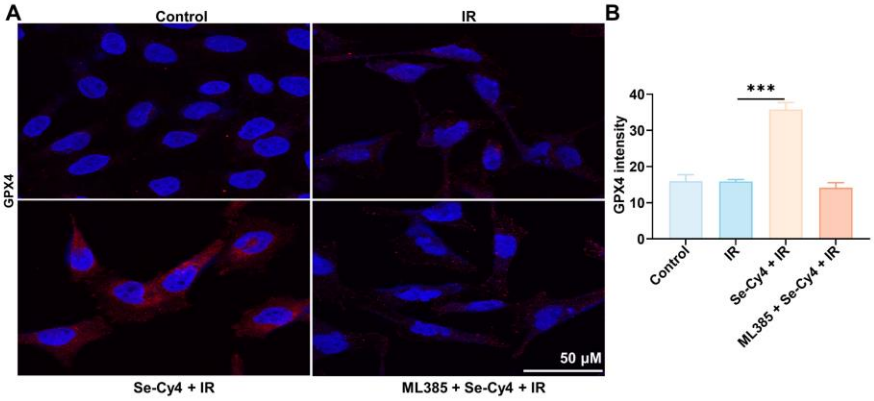

Figure 44. ML385 suppressed Se-Cy4–induced upregulation of GPX4 expression under irradiation. Immunofluorescence staining of GPX4 in control, IR, Se-Cy4 + IR, and ML385 + Se-Cy4 + IR groups. GPX4 was subsequently identified as a downstream effector regulated by Nrf2 under irradiation conditions. Nuclei were counterstained with DAPI. Scale bars, 50 μm.

Supplementary Figure 45

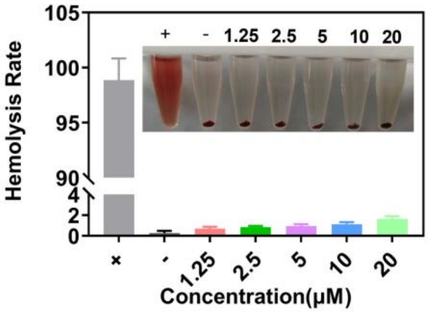

Figure S45. Hemolysis assay of Se-Cy4 at different concentrations (1.25, 2.5, 5, 10, and 20 μM). Data are presented as the mean ± standard deviation (n = 3)

Supplementary Figure 46

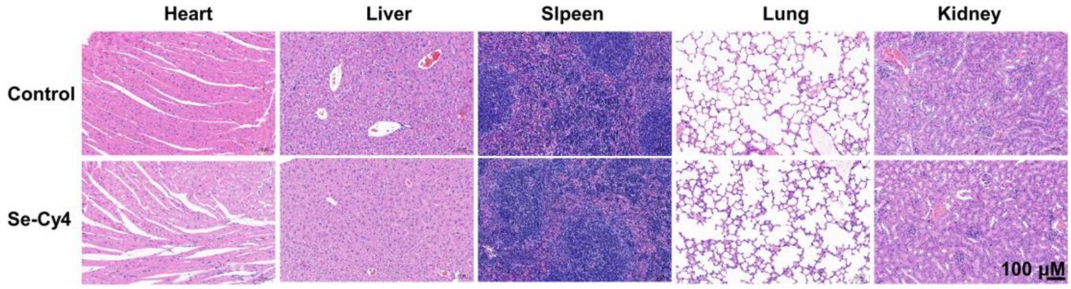

Figure S46. Histopathological analysis of major organs in mice treated with PBS or Se-Cy4 (20 mg/kg, once every three days for a total of five doses) with tissues collected and analyzed after 31 days of observation.

Supplementary Table

Table S1. The quantum chemical parameters of Se-Cy4 and IM-1- IM-3.

**Quantum chemical parameters**	**Se-Cy4**	**IM-1**	**IM-2**	**IM-3**	**IM-4**	**IM-5**	**IM-6**
$\boldsymbol{E}_{\mathbf{LUMO}}$ **(eV)**	-3.026	-3.031	-3.024	-3.016	-3.875	-1.266	-1.974
$\boldsymbol{E}_{\mathbf{HOMO}}$ **(eV)**	-5.052	-5.043	-5.046	-5.062	-6.804	-6.138	-5.681
$\boldsymbol{\Delta E}_{\mathbf{LUMO\text{-}HOMO}}$ **(eV)**	2.026	2.012	2.022	2.046	2.929	4.872	3.707
χ (eV)	4.039	4.037	4.035	4.039	5.340	3.702	3.828
η (eV)	1.013	1.006	1.011	1.023	1.465	2.436	1.854
CP (eV)	-4.039	-4.037	-4.035	-4.039	-5.340	-3.702	-3.828
$\boldsymbol{\omega}$	8.052	8.100	8.052	7.973	9.734	2.813	3.952
N	0.124	0.123	0.124	0.125	0.103	0.355	0.253

Parameters: lowest unoccupied molecular orbital (E_{LUMO}), highest occupied molecular orbital (E_{HOMO}), energy gap between E_{LUMO} and E_{HOMO} ($\Delta E_{LUMO\text{-}HOMO}$), electronegativity ($\chi$), absolute hardness ($\eta$), chemical potential (*CP*), electrophilicity index (ω), nucleophilicity index (*N*).
